# Characterization of a novel root-associated diazotrophic rare PGPR taxa, *Aquabacter pokkalii* sp. nov., isolated from pokkali rice: new insights into the plant-associated lifestyle and brackish adaptation

**DOI:** 10.1186/s12864-024-10332-z

**Published:** 2024-04-29

**Authors:** V. S. Sunithakumari, Rahul R. Menon, Gayathri G. Suresh, Ramya Krishnan, N. Rameshkumar

**Affiliations:** 1 Microbial Processes and Technology Division, National Institute for Interdisciplinary Science and Technology (CSIR), Thiruvananthapuram-695 019, Thiruvananthapuram, Kerala India; 2 Academy of Scientific and Innovative Research (AcSIR), Ghaziabad, 201002 India; 3 Athmic Biotech Solutions Pvt. Ltd. R&D Lab, Thiruvananthapuram, Kerala India

**Keywords:** Pokkali rice, Diazotroph, Seawater, PGPR, Aquabacter, ACC deaminase

## Abstract

Salinity impacts crop growth and productivity and lowers the activities of rhizosphere microbiota. The identification and utilization of habitat-specific salinity-adapted plant growth-promoting rhizobacteria (PGPR) are considered alternative strategies to improve the growth and yields of crops in salinity-affected coastal agricultural fields. In this study, we characterize strain L1I39^T^, the first *Aquabacter* species with PGPR traits isolated from a salt-tolerant pokkali rice cultivated in brackish environments. L1I39^T^ is positive for 1-aminocyclopropane-1-carboxylate deaminase activity and nitrogen fixation and can promote pokkali rice growth by supplying fixed nitrogen under a nitrogen-deficient seawater condition. Importantly, enhanced plant growth and efficient root colonization were evident in L1I39^T^-inoculated plants grown under 20% seawater but not in zero-seawater conditions, identifying brackish conditions as a key local environmental factor critical for L1I39^T^-pokkali rice symbiosis. Detailed physiological studies revealed that L1I39^T^ is well-adapted to brackish environments. In-depth genome analysis of L1I39^T^ identified multiple gene systems contributing to its plant-associated lifestyle and brackish adaptations. The 16S rRNA-based metagenomic study identified L1I39^T^ as an important rare PGPR taxon. Based on the polyphasic taxonomy analysis, we established strain L1I39^T^ as a novel *Aquabacter* species and proposed *Aquabacter pokkalii* sp nov. Overall, this study provides a better understanding of a marine-adapted PGPR strain L1I39^T^ that may perform a substantial role in host growth and health in nitrogen-poor brackish environments.

**Supplementary Information:**

The online version contains supplementary material available at 10.1186/s12864-024-10332-z.

## Background

In nature, the plant rhizosphere hosts a large and phylogenetically diverse group of complex bacterial communities whose interactions and activities are increasingly recognized as important determinants of plant health, fitness, and productivity [1]. The rhizosphere-associated bacteria that confer health benefits to plants are collectively called plant growth-promoting rhizobacteria (PGPR). The well-characterized beneficial functions of PGPR include 1) increasing the availability of essential nutrients inaccessible to the plants, such as nitrogen via fixation of atmospheric nitrogen [2] and phosphate via solubilization of insoluble soil phosphates through organic acid production [3], 2) promote plant growth and development via the production of phytohormones [4], 3) protection against plant pathogens through secreting different classes of antimicrobial substances or by eliciting plant defense hormones [5], and 4) provide enhanced protection against abiotic stress such as salinity and drought through scavenging plant stress ethylene with the help of 1-aminocyclopropane 1-dicarboxylate (ACC) deaminase [6]. Despite having huge potential in sustainable agriculture, the current knowledge about the PGPR and its potential beneficial interactions across a wide range of plants, including crops of brackish-associated environments, an interface between marine and terrestrial ecosystems that are agriculturally important, is very limited.

Pokkali is a unique traditional rice variety cultivated organically in water-logged brackish tidal environments of coastal regions of central Kerala, southern India. Notably, most of the pokkali rice fields are nearer to the sea. As a result, seawater enters these fields during each tidal cycle, leading to rapid fluctuations in salinity levels and creating an unfavorable environment for crop growth. Despite these constantly changing saline conditions, the pokkali rice grows well and yields good productivity. Thanks to its naturally evolved genetic traits enabling them to thrive at higher coastal salinity than any inland terrestrial rice varieties [7]. The other possible reason for their adaptability and better growth performance under such coastal salinity could be their close symbiotic relationships with the beneficial native microbes inhabiting these brackish pokkali rice fields. Till now, relatively little information is available on pokkali rice and its associated beneficial native microbes. Moreover, understanding the PGPR of such ecosystems may offer valuable insights into their physiology, genomic traits, habitat-specific adaptations, and functional role in plant health and productivity. Further, it can provide sustainable solutions for improving rice productivity under coastal saline conditions. For instance, very few studies have shown that microbes from the pokkali rice can promote growth and confer tolerance to salt stress in salt-sensitive rice varieties, *Fusarium* sp., in IR64 rice variety [8], and *Pseudomonas taiwanensis* strain PK7 in Uma rice variety [9].

In an ongoing research study on the isolation and functional characterization of the rhizosphere-associated PGPR of this unique brackish-associated pokkali rice, we specifically targeted habitat-specific indigenous PGPR strains that possess ACC deaminase activity and can promote rice growth under brackish water conditions. To achieve this, we cultured the rhizosphere samples of different pokkali rice varieties collected from various sites using different minimal media compositions that contained ACC as the sole nitrogen source and different sugars or organic acids as the carbon source, and 50% natural or 50% artificial seawater (Himedia, India) to mimic brackish environmental conditions. This investigation resulted in the isolation of several potential ACC deaminase-producing indigenous rhizobacterial strains, including the genera *Burkholderia*, *Herbaspirillum*, and *Rhizobium*, and a few unclassified strains that are characterized as novel bacterial taxa; *Vogesella oryzae* L3B39^T^ [10], *Arthrobacter pokkalii* P3B162^T^ [11], *Novosphingobium pokkalii* L3E4^T^ [12], *Pokkaliibacter plantistimulans* L1E11^T^ [13], *Sphingomonas pokkalii* L3B27^T^ [14], and *Flavobacterium pokkalii* L1I52^T^ [15], and functionally some are shown to possess plant growth-promoting abilities [13, 15]. Additionally, *Rhodoplanes pokkaliisoli* JA415^T^, a novel phototrophic alphaproteobacterium, was reported from a pokkali paddy field by Lakshmi et al. [16].

This study characterizes another novel ACC deaminase-producing diazotrophic strain designated L1I39^T^. Initial 16S rRNA sequence analysis using the EzBiocloud server [17] indicated that L1I39^T^ belongs to the genus *Aquabacter*. This genus belongs to the family *Xanthobacteraceae,* phylum *Pseudomonadota*, and was first described by Irgens et al., 1991 [18] with *Aquabacter spiritensis* SPL-1^ T^ as the type species. *Aquabacter* species are Gram-negative rods, non-motile, and aerobic heterotrophic bacteria that form yellow to white chalk-pigmented colonies. In addition to their taxonomic description, the possible ecological significance and detailed genome characterization in relationships with their growth habitats are lacking. At present, the genus *Aquabacter* contains two validly described species (https://lpsn.dsmz.de/genus/aquabacter), *A. spiritensis* SPL-1^ T^ isolated from Spirit Lake [18] and *A. cavernae* Sn-9-2^ T^ from cave soil [19]. The taxon described in this study would be the third and first marine member of the *Aquabacter* species isolated from a native salt-tolerant pokkali rice traditionally grown in brackish tidal environments of the coastal region of central Kerala, India. Based on the detailed genetic (including multilocus sequence and whole genome analysis), phenotypic, and chemotaxonomic characteristics, we establish that L1I39^T^ represents a novel *Aquabacter* species for which we named *Aquabacter pokkalii* sp nov. In addition, we functionally characterize its potential plant growth-promoting (PGP) properties, including ACC deaminase production, nitrogen fixation, and ability to colonize and promote rice growth under nitrogen-limiting seawater conditions. Through detailed physiological experiments, we demonstrate its eco-physiological adaptations relating to a brackish environment, and by analyzing the 16S rRNA amplicon metagenomic datasets of brackish-associated different native rice varieties of Kerala, we determined the occurrence and abundance of L1I39^T^ in those habitats. Finally, the genome of L1I39^T^ was sequenced and conducted an in-depth analysis to understand the molecular determinants that may contribute to its plant-associated lifestyle, plant-growth functions, and habitat-specific adaptations, including the brackish tidal environment. Further, we interrelate these genome data with results obtained from our phenotypic experiments.

## Materials and methods

### L1I39^T^ isolation, preservation, and seawater collection

The strain L1I39^T^ was isolated in 2014 from the rhizosphere of a salt-tolerant pokkali rice cultivated in water-logged brackish tidal environments of coastal Kerala, Kumbalangi (9°52′06.4’’N, 76°16′58.3’’E) India. For isolation, a traditional serial dilution method was followed [13]. The bacteriological medium A used for isolating L1I39^T^ has the following composition (g/100 ml): 0.1 g of malate, 3 mM of ACC (Sigma), 50 ml of artificial seawater (HiMedia), 50 ml of distilled water, 1.8 g of agar. The Reasoners 2A (R2A) agar (HiMedia catalog no SMEB962D) with 0.5% NaCl (R2A-N) or 20% natural seawater was used for routine subculture and to maintain L1I39^T^ at 4 °C. Cells were suspended in 20% glycerol and kept as glycerol stocks at -80 °C for long-term preservation. The natural seawater used in this study was collected from the Arabian Sea (~ 2 km from the Vizhinjam beach, Thiruvananthapuram, Kerala, India). The collected seawater was filter-sterilized in 8 µm cellulose nitrate filters (Sartorius Stedim Biotech, Germany) to remove particle traces and stored at 4ºC until used. The reference type strains *Aquabacter cavernae* KCTC 62308^ T^, *Aquabacter spiritensis* LMG 8611^ T^, *Azorhizobium caulinodans* LMG 6465^ T^, and *Xanthobacter autotrophicus* LMG 7043^ T^ were used for comparative taxonomic characterization.

### Polyphasic taxonomy

#### Molecular phylogeny

The extraction of genomic DNA, amplification of 16S rRNA gene, bi-directional Sanger sequencing, and sequence and phylogenetic analysis were performed following the described methods [13].

The high-quality DNA of L1I39^T^ for whole genome sequencing was extracted using the QIAamp DNA mini kit (Qiagen, Germany). The genome sequencing of L1I39^T^ was performed using the Illumina HiSeq 4000 sequencer at Bionivid Technology Pvt Ltd., Bengaluru, India. The raw sequencing reads were subjected to quality filtering (Phred score greater than 30) using the IlluQC tools available in the NGSQC toolkit [20]. The de novo assembly of the filtered high-quality reads was performed using the SPAdes v3.0.0 paired-end library pipeline [21]. A reference-based genome finishing was performed using the MeDuSa web server with *Xanthobacter autotrophicus* DSM 432^ T^ as the reference genome. The L1I39^T^ genome completeness, contamination, and heterogeneity were determined using CheckM v1.1.2 [22].

For multilocus sequence analysis (MLSA), six high-resolution phylogenetic taxonomic markers such as ATP synthase β subunit (*atpD*), recombinase A (*recA*), DNA gyrase subunit B (*gyrB*), molecular chaperone (*dnaK*), citrate (Si)-synthase (*gltA*) and RNA polymerase β subunit (*rpo*B) were selected. These protein-coding gene sequences were retrieved from the genomes of LI139^T^ and its related phylogenetic neighbors of the family *Xanthobacteraceae* (Table S1). The individual protein-coding gene sequence and phylogenetic tree analysis were performed as described before [11]. For concatenated sequence analysis, the protein-coding sequences of six housekeeping genes were aligned individually using the MAFFT multiple alignment program [23] and concatenated using the Gblocks tool available within PhyloSuite v1.2.2 [24]. The resultant concatenated protein-coding sequences were used for the construction of phylogenetic trees using different tree-making algorithms available in MEGA v7.0.26 [25]. Likewise, a phylogenomic tree was constructed using 539 single-copy orthologous protein-coding gene sequences obtained using OrthoFinder v2.5.4 [26]. *Escherichia coli* DSM 30083^ T^ served as the outgroup.

The genome-relatedness similarity analysis, including the average nucleotide identity (ANI), average amino acid identity (AAI), and digital DNA-DNA hybridization (dDDH) values, was performed between the genomes of L1I39^T^ and related phylogenetic neighbors of the family *Xanthobacteraceae* as described before [14, 27–29].

#### Chemotaxonomy and phenotype characters

Cells were cultivated in trypticase soy agar (TSA) to analyze cellular fatty acids. After incubation of 48 h at 28 °C, whole-cell fatty acid methyl esters (FAME) were extracted, and analysis was performed as described before [30]. The bacterial identification service available at DSMZ, Germany, was availed for determining the polar lipids and respiratory quinones of L1I39^T^. This analysis was performed using the protocol described by Tindall, 1990; Krishnan et al., 2018 [13, 31].

The phenotypic characteristics such as cell morphology, motility, anaerobic growth, reduction of nitrate, production of indole, Methyl red and Voges Proskauer (MR-VP), hydrolysis of starch, xylan, carboxymethyl cellulose, esculin, casein, urea, and DNA, and the ability to grow in different standard heterotrophic media, including Luria Bertani (LB), nutrient agar, and trypticase soya agar were performed using methods as described by Rameshkumar et al., 2016 [10]. The growth at varying temperatures and pH ranges was assayed using the procedure described by Rameshkumar et al., 2016 [11], with modifications specified by Menon et al., 2020 [15]. The assimilation of different substrates was determined using an API 20NE strip as per the instructions provided by the manufacturer, and the results were noted after 48 h of incubation at 30 °C.

### Genome functional analysis

The genome sequence was annotated using NCBI Prokaryotic Genome Annotation Pipeline (PGAP) and Rapid Annotation using Subsystem Technology (RAST) v.4 [32]. The different genes and gene clusters encoded in the L1I39^T^ genome under different functional gene categories were identified through the subsystem feature analysis in RAST. The rRNAs were predicted using RNAmmer-1.2 [33], and tRNAs were identified using ARAGORN-v1.2.36 [34]. The gene organization of the important operons was constructed according to the size and orientation of the genes using the genoPlotR package in R v2.6.0 [35]. The circular genome was constructed using the blast ring image generator, BRIG v0.95 [36], utilizing various functional gene categories identified using the EGGNOG-mapper v5.0 [37].

### Plant growth-promoting traits

#### ACC deaminase activity

The utilization of ACC as the sole nitrogen source and ACC deaminase quantification was determined by following the methods described by Krishnan et al., 2018 [13].

#### Growth in nitrogen-free medium and nitrogenase (nifH) gene amplification

The ability of L1I39^T^ to grow in a nitrogen-free medium was determined using Jensen’s medium (HiMedia, catalog no M710) with 0.5% NaCl. The presence of the *nifH* gene in L1I39^T^ was determined by polymerase chain reaction (PCR) based amplification of the *nifH* gene using the PolF and PolR universal *nifH* gene primers [38] and PCR cycling conditions: an initial denaturation step at 95 °C for 5 min (min), followed by 30 cycles consisting of denaturation at 95 °C for 45 s, annealing at 54 °C for 45 s, extension at 72 °C for 1 min, and a final extension step lasting 10 min. A single amplicon with a size of 360 bp indicated the presence of *the nifH* gene, and the genomic DNA of *Azoarcus communis* LMG 22127^ T^ was used as the positive control.

#### In-planta detection of L1I39^T^-nifH

Overnight grown L1I39^T^ cells from an R2A-N broth were centrifuged and washed, and the entire cell pellet was suspended in a sterile carbon and nitrogen-free minimal salt (MS) solution (consisting of 0.8 g/L of K_2_HPO_4_, 0.2 g/L of KH_2_PO_4_, 0.2 g/L of MgSO_4_.7H_2_O, 0.1 g/L Na_2._MoO_4_, 5 g/L NaCl, and a trace amount of yeast), making a final OD_600_ of 0.4 (~ 10^–6^ cells). Five days old sterile pokkali rice seedling roots were incubated in this bacterial MS solution for 90 min. The seedlings incubated in MS solution without L1I39^T^ cells were the control. These incubated seedlings (L1I39^T^-treated and control) were transferred aseptically to the respective phytajars containing semi-solid MS media (prepared with 0.2% agar to create a microaerophilic condition). The entire setup was incubated in plant growth racks maintained with light/dark (12 h/12 h) cycles under 28 °C for five days. The same procedure was followed for nitrogen-excess conditions, except the seedlings were incubated in a semi-solid MS media prepared with 20 mM NH_4_Cl. Total RNA was extracted from the incubated root samples using the RNeasy Plant Mini-Kit (Qiagen, Germany). The RevertAid First Strand cDNA Synthesis kit (Thermo Scientific, USA) was used for complementary DNA synthesis. The RNA-converted cDNA samples were subjected to PCR amplification using the universal *nifH* gene primers PolF/PolR [38] and amplification conditions (as mentioned above). A single amplicon at 360 bp was considered positive.

#### NifHDK phylogeny and nif gene cluster comparison

Annotated protein sequences of NifH, NifD, and NifK were obtained from the genomes of LI139^T^ and its related phylogenetic neighbors, including the members of the genera *Aquabacter*, *Azorhizobium*, and *Xanthobacter*. These protein sequences were aligned individually using the MAFFT multiple alignment program [23] and concatenated using the Gblocks tool available within PhyloSuite v1.2.2 [24]. The phylogenetic trees were constructed using the concatenated protein sequences of NifHDK using MEGA v7.0.26 [25]. For the *nif* gene cluster comparison, the software program genoPlotR package was used [35]. The following reference strain genomes were included: *Aquabacter cavernae* Sn-9-2^ T^, *Aquabacter spiritensis* DSM 9035^ T^, *Azorhizobium caulinodans* ORS 571^ T^, *Xanthobacter autotrophicus* Py2 as well as four well-characterized diazotrophs associated with terrestrial rice plants namely, *Azoarcus* sp BH72, *Azospirillum brasilense* Sp7^T^, *Azotobacter vinelandii* CA, and *Pseudomonas stutzeri* DSM 4166.

#### Siderophore production

The production of siderophore was determined by following the protocol described by Krishnan et al., 2017 [12], except Luria Bertani was used as the basal growth medium. The development of a yellow-colored clearance zone surrounding the grown colonies after a week of incubation is considered positive.

#### In planta growth experiment

The ability of L1I39^T^ to promote host growth under nitrogen-poor seawater conditions was investigated using pokkali rice. Briefly, three days old sterile germinated pokkali seeds were incubated with L1I39^T^ (≥ 10^8^ cells) suspensions for three hours, followed by a brief drying, and then aseptically transferred to pots containing sterile soil rite mix: sand (2:1) as growth substrates. Germinated seeds treated without L1I39^T^ cells served as control. In order to maintain a brackish environmental condition, the growing seedlings were irrigated regularly with 20% natural seawater, and the nitrogen-free half-strength Hoagland solution containing 20% natural seawater was supplemented as a plant nutrient solution every 3-days once till the end of the experiment. After 18 days of growth, both L1I39^T^-treated and untreated control plants were uprooted and quantified for various plant growth-related parameters, including the shoot fresh weight, fresh and dry weight of the roots, shoot and root length. The nitrogen content of the shoots was estimated using the KjelTRON™ KDIGB 8 M system according to the manufacturer’s instructions (Tulin Equipments, India). To validate whether 20% natural seawater condition is critical for L1I39^T^ plant growth promotion. A similar plant growth experiment was performed as detailed above, but this time, the germinated seeds, including the L1I39^T^-treated and control, were grown under nitrogen-free zero seawater conditions. The nitrogen-free half-strength Hoagland solution with zero seawater was supplemented as a plant nutrient solution. The plant parameters were recorded after 28 days post-inoculation. The comparative bar graphs were plotted for the quantified plant parameters to represent the differences between the L1I39^T^-treated and untreated control plants. The statistical significance was determined through the student’s t-test, and the significance was indicated in the graphs as follows: *, *p*-value < 0.05; **, *p*-value < 0.01; ***, *p*-value < 0.005.

### Plant-associated traits

#### Hydrogen peroxide tolerance

Active L1I39^T^ cells were inoculated to an LB broth containing 20% seawater and incubated at 30 °C, 180 rpm. When the cells reached an OD_600_ of 0.2 (~ 10^4^ cells), different concentrations of hydrogen peroxide (5 mM and 10 mM) were added to the culture broths, respectively, and allowed to grow further for 24 h. The culture broth with no added hydrogen peroxide served as a control. The hydrogen peroxide tolerance was determined by monitoring the visible growth of the test against the control after 24 h.

#### Biofilm formation

The biofilm formation ability of L1I39^T^ cells was tested in 96-well microtiter plates using the method reported by Krishnan et al., 2016 [11], except R2A-N broth was used as the growth medium. The formation of a crystal violet stained ring on the walls of the microtiter plate was regarded as positive for biofilm formation.

#### Metabolic capacity

The ability to utilize different plant-derived substrates, including various sugars, amino acids, and organic acids, was determined using methods described by Krishnan et al., 2017 [12]. The assimilation of other carbon sources was determined using the Biolog Gen III system, and the results were noted after 48 h of incubation at 30 °C.

#### Cell–cell contact-dependent killing assay

The ability of L1I39^T^ cells to inhibit the neighboring cells via cell–cell contact was performed as described previously [39]. L1I39^T^ was used as an attacker strain, and *E*. *coli* harboring pHC60-GFP plasmid with tetracycline resistance was used as a target strain. *Pokkaliibacter plantistimulans* L1E11^T^, known for its contact-dependent killing ability, was used as the positive control. Active cells of both attacker and target strains were suspended in 0.85% saline, normalized to an OD_600_ of ~ 0.9, and mixed in a volume ratio of 10:1 (attacker vs target). 20 µl this mixture was spotted on a solid LB agar medium and incubated for 36 h at 30 °C. Surviving *E*. *coli* target cells were enumerated by standard serial dilution and counting viable colonies on tetracycline-supplemented LB agar medium. The final results were quantified from the average of three independent experiments.

### Ecophysiological conditions

Growth at different concentrations of NaCl (0.5, 1, 1.5, 2, 3, and 4%, w/v) was tested by inoculating the L1I39^T^ cells in R2A broth (HiMedia catalog no LQ251X) containing respective NaCl concentrations and incubated for a week at 30 °C. The culture flasks showing good turbidity were considered positive.

The ability to grow in different natural seawater concentrations (0, 20, 40, 60, 80, 100%) was determined using R2A broth as the base growth medium. Active L1I39^T^ cells were inoculated in R2A broth with respective seawater concentrations and incubated for a week at 30 °C. The optimal seawater concentration for growth was determined by measuring the OD _600_ of R2A broth containing respective seawater concentration at 12 h intervals for a total period of 60 h.

The ability to grow in ZoBell marine medium was checked by streaking a single colony of L1I39^T^ on ZoBell marine agar medium (HiMedia catalog no M385) and evaluating its growth after seven days of incubation at 30 °C.

### L1I39^T^ GFP tagging, root attachment, and colonization

The L1I39^T^ was tagged with a pLH6000 plasmid harboring green fluorescent protein (GFP) by following the method described [40]. Briefly, the electrocompetent cells were prepared by suspending the late log phase of L1I39^T^ cells in 10% ice-cold glycerol. The plasmid pLH6000 was transformed into electrocompetent L1I39^T^ cells using the electroporation conditions: 2.5 kV, 200Ω, and 25µF. Immediately after electroporation, the L1I39^T^ cells were grown in a fresh SOC broth [41] for 4–6 h. After this, the cells were centrifuged, and the resulting cell pellet was spread-plated on R2A-N agar plates containing 50 µg spectinomycin. After three days of incubation at 30 °C, the spectinomycin-resistant L1I39^T^ colonies were picked, checked for stable GFP transformation, and later used for root colonization and imaging studies.

The ability to attach host roots under a brackish environmental condition was determined by incubating the sterile five days old pokkali rice seedling roots in GFP-tagged L1I39^T^ suspension prepared in 20% seawater. After 3 h of incubation, the roots were excised and washed two to three times in sterile 20% seawater to remove loosely adhered cells. Following this, the roots were placed on a clean glass slide with 40% glycerol, and then a clean glass coverslip was placed over the roots and was used for visualization under the epifluorescent microscope. Imaging was performed using a Zeiss AXIO Imager M2 fluorescent imaging microscope fitted with a Zeiss Axiocam 503 mono camera (Zeiss, Germany), and the images were processed in ZEISS ZEN (version 2.6) software. The green fluorescence signals emitted from the GFP-tagged L1I39^T^ cells attached along the pokkali seedling roots were considered positive for root attachment.

For root colonization*, o*vernight-grown GFP-tagged L1I39^T^ cells were suspended in a nitrogen-free half-strength Hoagland plant nutrient solution. 1 ml of this GFP-tagged L1I39^T^ (~ 10^6^ cells) suspension was inoculated into gnotobiotic phytajars containing five days old pokkali rice seedlings growing in a nitrogen-free half-strength Hoagland nutrient solution prepared with 20% seawater. This entire phytajar setup was incubated in the plant growth racks (12 h of light and 12 h of dark at 28 °C). Roots were collected on the 7th and 14th day post-inoculation and used to estimate the root colonization potential by following the described methods [12]. The roots were processed for imaging purposes, as mentioned above in the root attachment section. To determine the colonization ability of L1I39^T^ under zero seawater conditions, the same experimental setup was carried out as described above, except the pokkali rice seedlings were grown in a nitrogen-free half-strength Hoagland nutrient solution with no seawater added.

### Mapping of operational taxonomic units (OTUs) of L1I39^T^ from brackish-associated rice metagenomic datasets

To identify OTUs of L1I39^T^, we mapped the full-length 16S rRNA gene sequence of L1I39^T^ against the 16S rRNA amplicon metagenomic datasets of brackish-associated different rice varieties of coastal Kerala, India, generated from our earlier study (NCBI Accessions: pokkali rice—PRJNA970257 and kaipad—PRJNA970551, unpublished results). Only those 16S rRNA sequences showing > 99% similarity and > 400 bp length were considered as representative OTUs of L1I39^T^. For this analysis, we used the latest version of the NCBI-BLAST program (v2.7.1 +).

### Statistical analysis

All experiments were performed with at least three technical replicates, and the data were analyzed using Student’s t-test and the software GraphPad Prism version 8. Error bars in the graph represent standard deviation within the replicates, and the statistical significance is represented with an asterisk (*) sign as follows: *, *p*-value < 0.05; **, *p*-value < 0.01; ***, *p*-value < 0.001.

### Availability of data and materials

The 16S rRNA gene sequence of L1I39^T^ was deposited in NCBI under accession number KX533957. The draft genome sequence of L1I39^T^ was deposited in NCBI with accession number CP072392. The genome accession numbers of reference-type strains of the family *Xanthobacteraceae* used in this study are listed in Table S1.

## Results and discussion

### Proposal for *Aquabacter pokkalii* sp. nov.,

#### Phylogenetic analysis

**Fig. 1:**
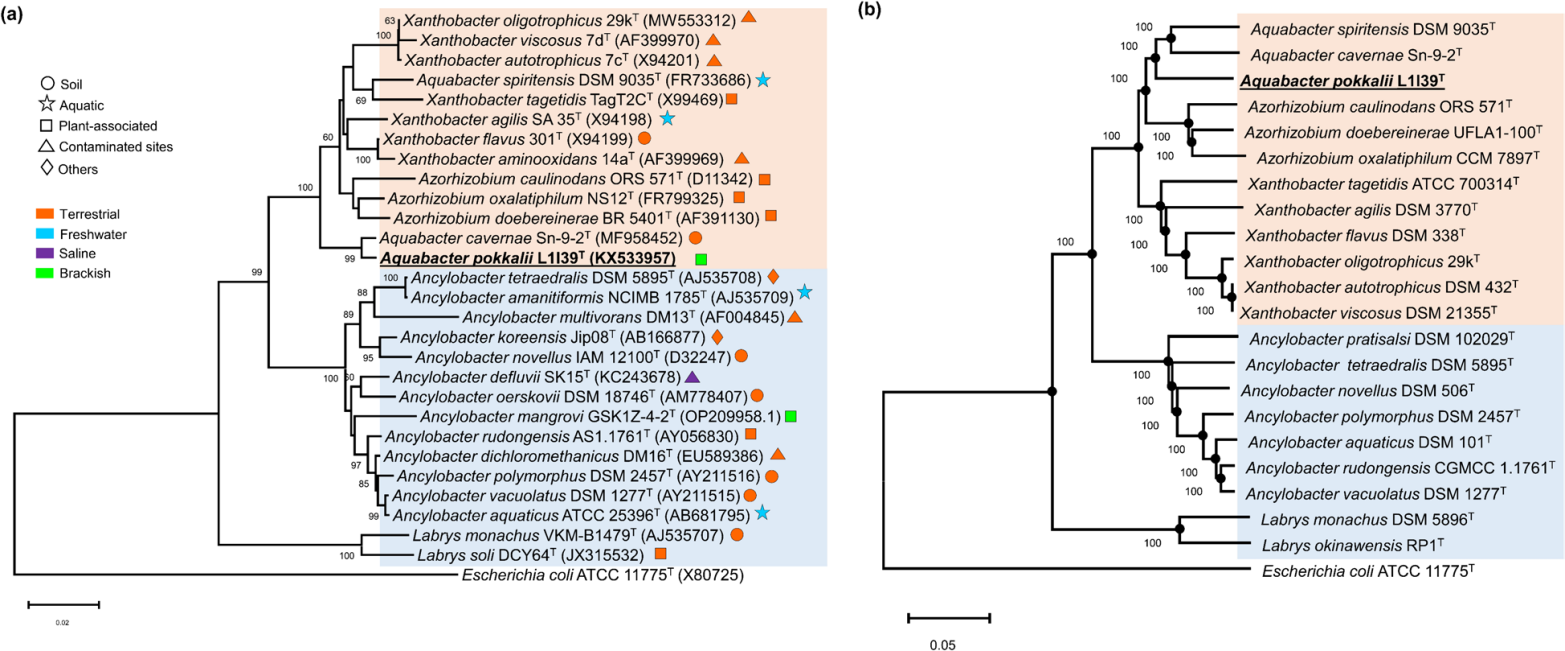
**a** Maximum likelihood phylogenetic tree based on the 16S rRNA gene sequences of L1I39^T^ and related members of the family *Xanthobacteraceae*. The significance of each branch is indicated by the bootstrap value (as a percentage) calculated for 1000 subsets. The bar indicates sequence divergence. Different shapes and color codes indicate the isolation habitats of the respective type strains, (**b**) maximum likelihood phylogenetic tree based on the protein-coding sequences of 539 core genes (overall 170,910 amino-acid positions) of L1I39^T^ and related members of the family *Xanthobacteraceae*. Closed dark circles at each node represent a similar grouping obtained from the neighbor-joining and maximum parsimony algorithms. The significance of each branch is indicated by the bootstrap value (as a percentage) calculated from 1000 subsets. *E. coli* ATCC 11775^ T^ was used as an outgroup. The scale bar indicates nucleotide substitution per site

The 16S rRNA gene analysis identified strain L1I39^T^ as a member of the family *Xanthobacteraceae*, sharing the highest pairwise 16S rRNA gene sequence similarities with *Aquabacter cavernae* Sn-9-2^ T^ (99.00%), followed by *A. spiritensis* DSM 9035^ T^ (97.23%), *Xanthobacter oligotrophicus* 29k^T^ (97.01%) and *Xanthobacter autotrophicus* 7c^T^ (96.94%). Pairwise 16S rRNA sequence similarities to all other recognized members of the family *Xanthobacteraceae* were below 96.94%. Additionally, a 16S rRNA phylogenetic tree reconstructed using the maximum likelihood method showed that strain L1I39^T^ clustered stably with *A. cavernae* Sn-9-2^ T^ as the closest known *Aquabacter* species with a bootstrap support value of 99% (Fig. 1a). Notably, this cluster is well-positioned as an independent branch that was well-separated from a phylogenetic group comprising *A. spiritensis* DSM 9035^ T^ and remaining all other known species of the genus *Xanthobacter* and *Azorhizobium* with a bootstrap support value of 100% (Fig. 1a). Furthermore, these results indicate that strain L1I39^T^ is phylogenetically more closely related to the cluster comprising the members of the genera *Aquabacter*, *Xanthobacter*, and *Azorhizobium* rather than to the phylogenetic cluster containing other described members of the family *Xanthobacteraceae*; *Ancyclobacter* and *Labrys* (Fig. 1a). Similar phylogenetic positioning of L1I39^T^ was obtained in other 16S rRNA tree construction methods, neighbor-joining and maximum parsimony (data not shown). Notably, the 16S rRNA similarity value between the strain L1I39^T^ and its phylogenetic nearest type strain *A. cavernae* Sn-9-2^ T^ was well above the standard boundary 16S rRNA value of 98.65% to differentiate two different bacterial species [42]. Therefore, high-resolution phylogenetic taxonomic markers such as *atpD*, *recA*, *gyrB*, *dnaK*, *gltA,* and *rpo*B were retrieved from the L1I39^T^ genome and used further to clarify its taxonomic positioning in the family *Xanthobacteraceae*. The analysis of individual and concatenated sequences of the six housekeeping genes (*atpD*, *recA*, *gyrB*, *dnaK*, *gltA*, and *rpoB*) revealed that strain L1I39^T^ shared relatively lower gene sequence similarities (< 93%, < 90%, < 86%, < 91%, < 91%, < 92% and < 90%, respectively) with its closest phylogenetic relatives (Table S2). Importantly, these gene sequence similarity values are relatively lower than the commonly proposed threshold cut-off similarity value of 94% for novel bacterial species description [43]. Furthermore, the phylogenetic trees inferred using both individual (data not shown) and concatenated amino acid sequences of the above six housekeeping genes showed that strain L1I39^T^ was placed in a separate branch along with the type strains of *A. cavernae* Sn-9-2^ T^ and *A. spiritensis* DSM 9035^ T^ as its closest phylogenetic neighbors with a bootstrap support value of 91% (Fig. S1). Similar phylogenetic positioning of L1I39^T^ was retrieved when we constructed a protein-based core phylogenomic tree using 539 genes (Fig. 1b). Altogether, distinct phylogenetic position and low levels of housekeeping gene sequence similarities with the described members of the genus *Aquabacter* indicate that strain L1I39^T^ may represent a novel *Aquabacter* species.

#### Genome-based relatedness

Estimating the genome-relatedness similarity values, including ANI, AAI, and dDDH for species-level bacterial classifications, is considered the gold standard [44, 45]. Accordingly, we estimated the genome-relatedness similarity values between the genomes of L1I39^T^ and its phylogenetically closest *Aquabacter* type strains and with other recognized members of the family *Xanthobacteraceae*. This result showed that the calculated values between the genomes of L1I39^T^ and *A. cavernae* Sn-9-2^ T^ and *A. spiritensis* DSM 9035^ T^ and other recognized members of the family *Xanthobacteraceae* (Table S3) are relatively lower than the standard proposed ANI/AAI/dDDH values (< 95%, < 95%, and < 70%) for novel bacterial species delineation [44, 45], suggesting that strain L1I39^T^ represent as a distinct *Aquabacter* species within the family *Xanthobacteraceae*.

#### Chemotaxonomic and phenotypic characterization

The fatty acids of the strain L1I39^T^ were dominated by C18:1*ω*7c (88.79%), which is in accordance with the members of the family *Xanthobacteraceae* [46]. However, the fatty acids profiles that may be useful in differentiating the strain L1I39^T^ from its nearest phylogenetic neighbors are mentioned in (Table S4). The strain L1I39^T^ contained ubiquinone Q-10 as the predominant quinone and major polar lipids as phosphatidylglycerine, phosphatidylethanolamine, phosphatidylglycerol, and phospholipid (Fig. S2). All these chemotaxonomic data are in agreement with earlier published reports on the different species of the family *Xanthobacteraceae* [47].

The phenotypic characters that differentiate strain L1I39^T^ from its closest phylogenetic neighbors, *Aquabacter*, *Xanthobacter*, and *Azorhizobium,* are colony pigmentation, growth in ZoBell marine medium, tolerance to 3% NaCl, and growth in the presence of full-strength seawater (Table 1).

**Table 1:** Major phenotypic characters differentiating strain L1I39^T^ from its nearest phylogenetic neighbors

Characteristic traits	1	2	3	4	5
Colony colour	Cream	Yellow	Chalk white	Cream	Yellow
Oxidase	-	+	+	+	+
Growth at 42° C	-	-	-	+	-
**Growth in:**					
ZoBell marine agar	+	-	-	-	-
3% NaCl	+	-	-	-	-
100% seawater	+	-	-	-	-
ACC as a nitrogen source	+	-	-	ND	ND
ACC deaminase activity	+	-	-	ND	ND
G + C content (%)^a^	66.9	67.5	67.6	67.3	67.5

1, L1I39^T^; 2, *A. cavernae* KCTC 62308^ T^; 3, *A. spiritensis* LMG 8611^ T^; 4, *A. caulinodans* LMG 6465^ T^; 5, *X. autotrophicus* LMG 7043^ T^. All data are from this study; symbols: + , positive; -, negative, ND Not done. ^a^G + C content derived from genome

#### Plant growth-promoting properties

**Fig. 2:**
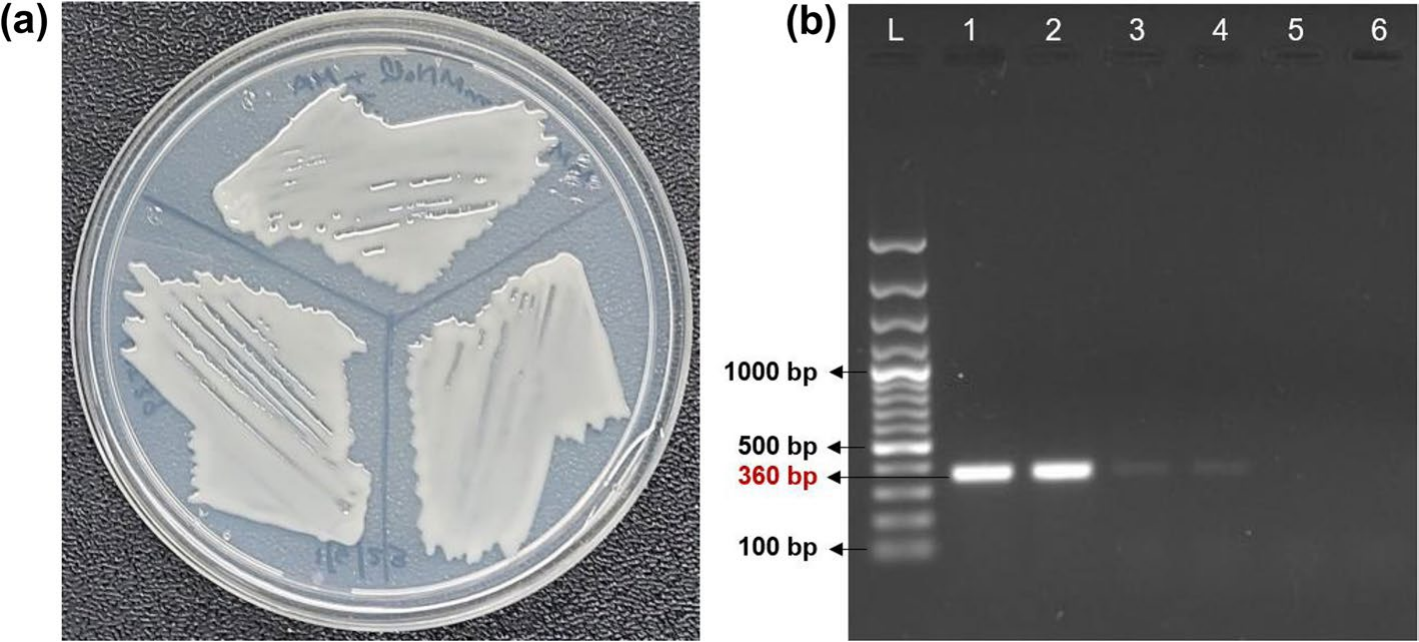
The plant-growth-promoting traits of L1I39^T^: (**a**) positive growth of L1I39^T^ on a defined minimal salts medium containing ACC as the sole nitrogen source, images taken after 4 days of incubation at 30 °C, (**b**) detection of *In-planta* L1I39^T^ *nifH* gene expression, lanes, L, 100 bp plus DNA ladder; 1 & 2, *nifH* gene amplicon from total root RNA converted cDNA from nitrogen-free conditions; 3 & 4, *nifH* gene amplicon from total root RNA converted cDNA from nitrogen-excess conditions; 5, the total root RNA converted cDNA from pokkali seedlings roots incubated without L1I39^T^ cells (negative control); 6, no reverse transcriptase (RT) control. Amplicon size of 360 bp is indicated in the gel image

L1I39^T^ can utilize ACC as the sole nitrogen source when grown in a defined mineral salts media (Fig. 2a) and exhibited ACC deaminase activity of 1.72 ± 0.89 µmol of α-ketobutyrate/h/mg of protein. Furthermore, the L1I39^T^ genome contains a putative gene encoding for ACC deaminase (*acdS*), suggesting that the *acdS* gene is functional. Additional analysis showed that L1I39^T^ tested positive for growth in a nitrogen-free Jensens medium, followed by a PCR amplification confirming the presence of the 360 bp region of the *nifH* gene (data not shown), a universal marker widely used for detecting diazotrophic bacteria [38]. Besides, the L1I39^T^ genome carries a complete set of essential genes required for biological nitrogen fixation (detailed in the genome section), suggesting that *nif* regulon is functional. In support of this view, an in-planta *nifH* gene expression assay showed that the *nifH* gene transcripts of L1I39^T^ were detected in the pokkali rice roots when grown under a nitrogen-deficient condition (Fig. 2b). However, this *nifH* gene expression was repressed by free excess ammonium present in the growth medium (Fig. 2b), indicating that L1I39^T^ regulates its *nifH* gene expression according to the status of nitrogen availability in the growth environment [48]. Furthermore, these findings suggest the significant ability of L1I39^T^ to fix atmospheric nitrogen in the host roots under a nitrogen-deficient growth condition.

**Fig. 3:**
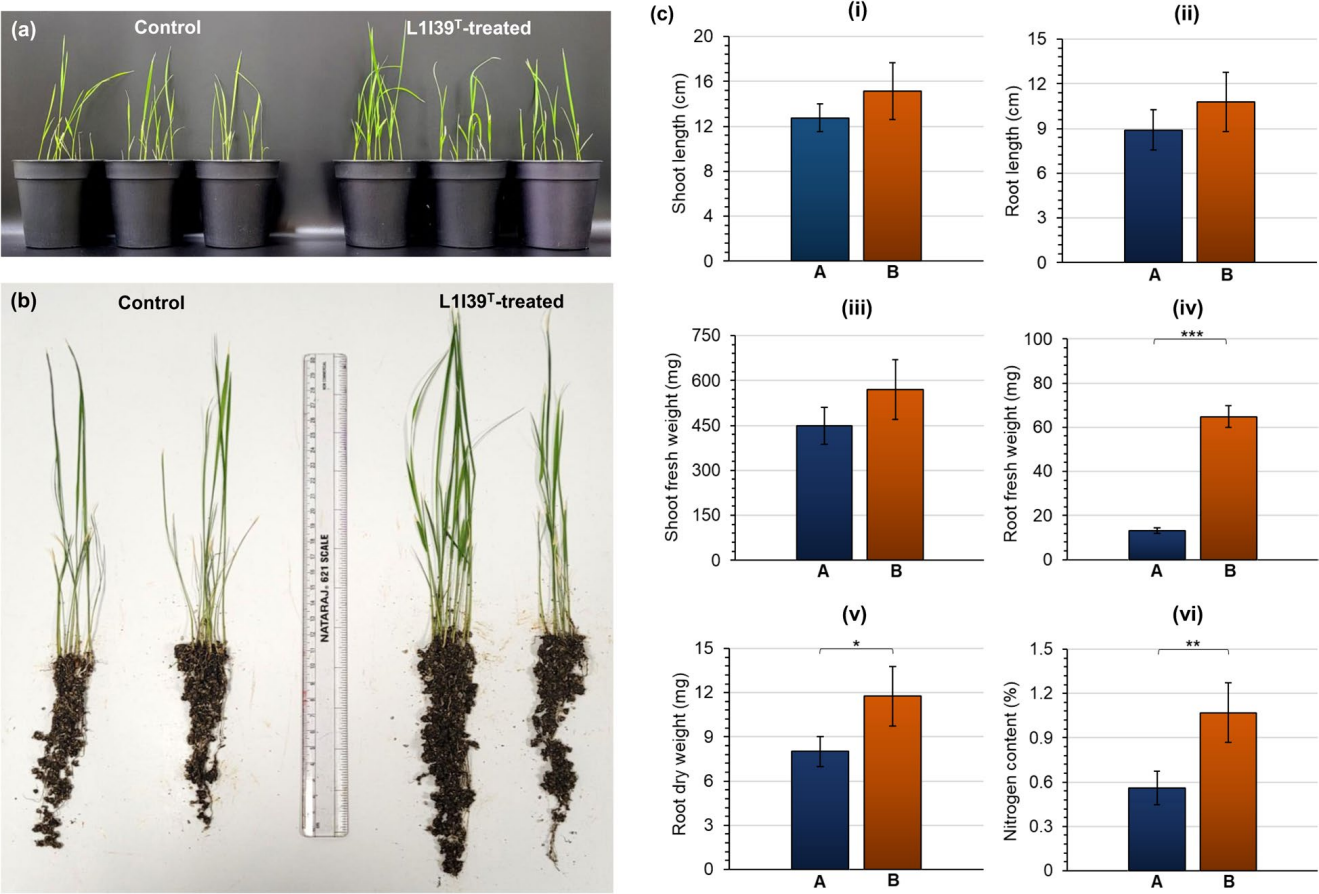
Plant growth-promoting effects of L1I39^T^ under nitrogen-limiting seawater conditions, (**a**) pot images showing the growth of pokkali rice (L1I39^T^-treated and control plants) after 18 days post-inoculation, (**b**) uprooted images showing the growth differences between the L1I39^T^-treated and control plants after 18 days post-inoculation, (**c**) bar graphs represent the significant growth differences observed in plant parameters such as (**i**) shoot length, (**ii**) root length, (**iii**) shoot fresh weight, (**iv**) root fresh weight, (**v**) root dry weight, and (**vi**) nitrogen content (%) in shoots between (**A**) control and (**B**) L1I39.^T^-treated pokkali plants estimated after 18 days post-inoculation. Statistical significance represented by *, *p*-value < 0.05; **, *p*-value < 0.01; ***, *p*-value < 0.001

Following these observations, we investigated the plant growth-promoting ability of L1I39^T^ under a nitrogen-deficient seawater condition that mimics or is nearer to the physiological state of brackish pokkali rice fields. For this purpose, surface-sterilized pokkali rice seeds were treated with L1I39^T^ and transferred to pots containing a sterile soil rite sand mixture (2:1 ratio). Untreated seeds were used as a control. The entire setup was irrigated with a 20% natural seawater concentration, and a nitrogen-free plant nutrient solution containing 20% natural seawater was provided to the rice plants once every three days until the experiment was complete. On the 7th dpi (days post inoculation), no visible growth difference was observed between the L1I39^T^-treated and untreated control plants (data not shown). However, on the 18th dpi, we observed better growth performance of the pokkali rice plants treated with L1I39^T^ than the untreated control plants (Fig. 3a & b). This observation includes a significant increase in the plant parameters, such as the fresh and dry weight of the roots (Fig. 3c). Likewise, we also observed an increase in the root and shoot length and the shoot fresh weight in rice plants treated with L1I39^T^ (Fig. 3c). Nevertheless, this data is not statistically found significant. Notably, a substantial increase in the nitrogen content of the shoot portions was observed in rice plants treated with L1I39^T^ compared to untreated control plants (Fig. 3c), indicating its ability to provide fixed nitrogen inputs to the host pokkali rice when grown under a nitrogen-limiting condition corroborating above in planta *nifH* gene expression assay. Altogether, these results suggest the potential ability of L1I39^T^ to enhance the pokkali rice growth by supplying fixed nitrogen, an essential plant macronutrient, in a nitrogen-limiting brackish environmental condition, thereby identifying strain L1I39^T^ as an important PGPR taxon.

Most members of the family *Xanthobacteriaceae* described are associated with terrestrial plants [49]. However, only very few members have shown to possess PGP abilities, including the well-characterized stem nodulating nitrogen-fixing strain, *Azorhizobium caulinodans* ORS 571^ T^ [50], and a few different strains of *Xanthobacter autotrophicus* [51, 52]. Moreover, none of these studies tested their plant growth promotion ability under a nitrogen-limiting seawater growth condition. Notably, no reports show direct experimental evidence that strains of *Aquabacter* possess PGPR properties. Hence, this study describes strain L1I39^T^ as the first *Aquabacter* species from a brackish-associated pokkali rice shown to possess multiple PGPR traits, including biological nitrogen fixation and ACC deaminase production and can potentially promote pokkali rice growth in nitrogen-limiting seawater conditions.

#### Eco-physiological adaptations

Pokkali rice fields are highly prone to seawater intrusions, and to sustain in these habitats, native microbes need to be well-adapted to higher and constantly changing salinity conditions. Studies have shown that most strains belonging to the genera *Aquabacter*, *Xanthobacter*, and *Azorhizobium* of the family *Xanthobacteraceae* are from terrestrial environments, are more sensitive to NaCl, and can tolerate only up to 1% NaCl [19]. In this regard, a NaCl tolerance test was performed and found that L1I39^T^ could grow in NaCl concentrations ranging from 0 to 3% NaCl. Furthermore, we show that L1I39^T^ can grow in ZoBell marine medium, a synthetic marine medium specifically used for cultivating heterotrophic marine bacteria (Fig. S3). Notably, the nearest phylogenetic neighbors of L1I39^T^ tested negative for growth at 3% NaCl and in ZoBell marine medium (Table 1). Besides, we also tested the ability of L1I39^T^ to grow in R2A broth prepared with varying natural seawater concentrations ranging from 20 to 100%. This result showed that L1I39^T^ could grow very well in 20 and 40% natural seawater but displayed weak and delayed growth at higher seawater concentrations (> 60%), suggesting that higher seawater concentrations had some negative growth effects on L1I39^T^ (Fig. S4). Importantly, L1I39^T^ showed an optimal growth at 20% natural seawater compared to zero-seawater conditions (Fig. S4), signifying that lower seawater concentrations can influence the L1I39^T^ growth, a habitat-specific trait typically observed in bacteria originating from brackish-associated environments.

**Fig. 4:**
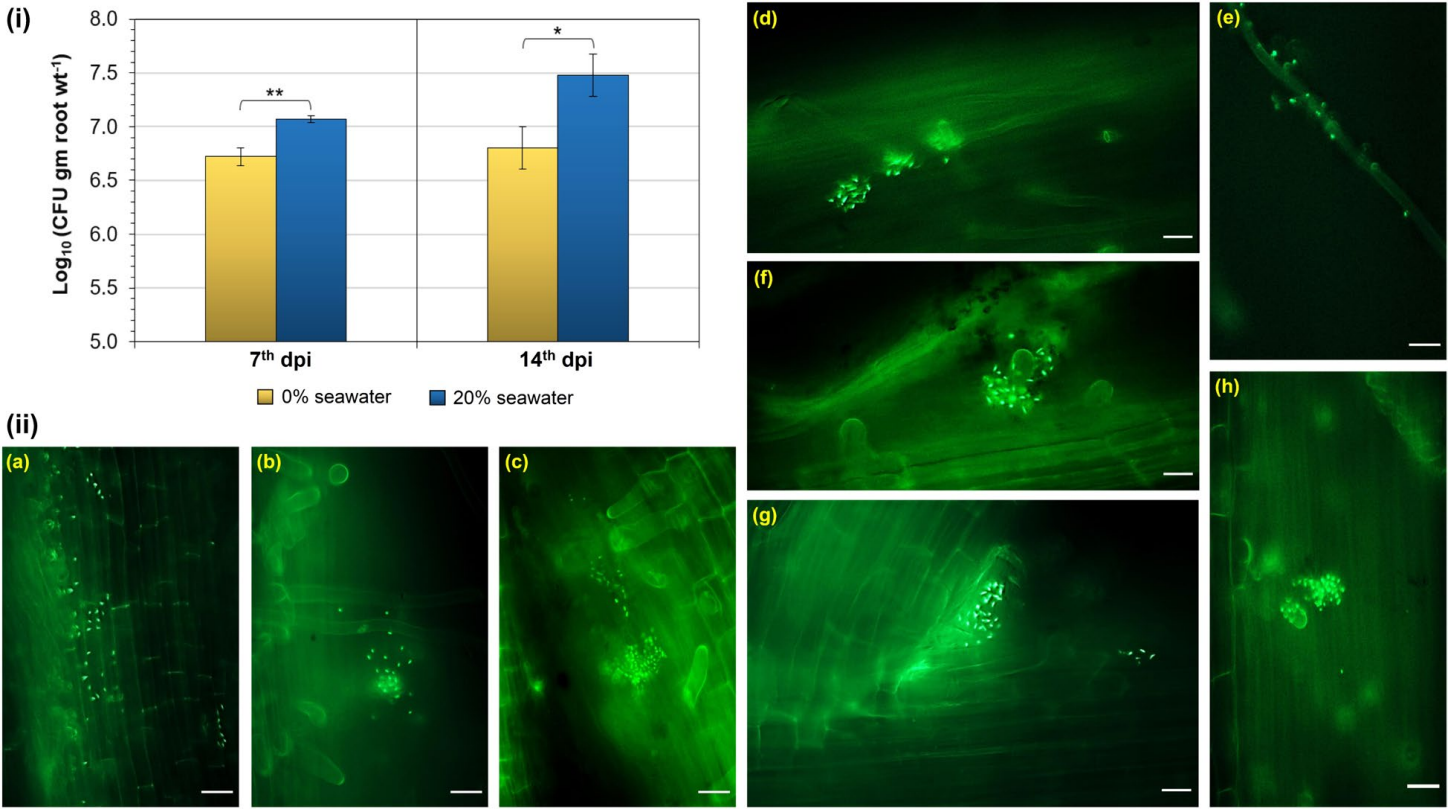
**i** The comparative pokkali rice root colonization potential of GFP-tagged L1I39^T^ cells between 20% seawater and zero-seawater conditions quantified after the 7th and 14th days post-inoculation is shown as a bar graph. Statistical significance is represented by *, *p*-value < 0.05; **, *p*-value < 0.01. **ii** Epi-fluorescent images showing the pokkali rice root colonization of GFP-tagged L1I39^T^ cells under 20% seawater conditions, (**a** & **d**) the primary roots (7th dpi), (**b** & **c**) the root hairs of primary root (7th dpi), (**e**) the lateral root (7th dpi), (**f** & **h**) the root hairs of primary root (14th dpi), and (**g**) the primary root-lateral root junction (14th dpi). Scale bars (20 µm) are indicated in all the microscopic images

Next, we investigated the colonization ability of the L1I39^T^ in the presence and absence of a seawater condition that was used earlier in our plant growth experiments. For this purpose, five days old sterile pokkali rice seedlings were grown in separate gnotobiotic phytajars containing respective nitrogen-free plant nutrient solutions containing 20% seawater and zero seawater. To this setup, 10^5^ cells (CFUs/ml) of GFP-tagged L1I39^T^ were inoculated and incubated in a plant growth chamber for 14 days. On 7th dpi, we recovered L1I39^T^-GFP cells from the roots of zero seawater (5.47 (± 1.10) X 10^6^ CFUs/gm of roots) and 20% seawater (1.20 (± 0.08) X 10^7^ CFUs/gm of roots) conditions. Further, at 14th dpi, L1I39^T^-GFP cells reached 7.10 (± 2.68) X 10^6^ and 3.37 (± 1.54) X 10^7^ CFUs/gm of roots in zero seawater and 20% seawater conditions, respectively (Fig. 4i). Notably, we observed that 20% seawater conditions enhanced the colonization ability of L1I39^T^-GFP cells by one log fold compared to zero seawater conditions, indicating its ability to interact with pokkali rice roots more efficiently in a brackish environmental condition. This view is in agreement with the above in planta growth experiments, where L1I39^T^-treated plants showed enhanced growth when grown under a 20% seawater condition (Fig. 3a, b & c). In contrast, no significant difference in the plant growth parameters was observed between the L1I39^T^-treated and untreated control plants when grown under zero seawater conditions (Fig. S5). These results potentially indicate that a brackish environment plays a vital role in L1I39^T^-pokkali rice symbiosis. Also, it suggests that L1I39^T^ is well-adapted physiologically to its brackish growth habitat, which is critical for pokkali rice interactions.

Through epifluorescence microscopy, we show that L1I39^T^-tagged GFP cells colonize the pokkali rice seedlings primarily on the root surfaces, including the primary roots, lateral root junctions, and root hairs (Fig. 4ii). However, the colonization pattern of L1I39^T^ cells on the root surfaces is not uniform but occurs as patches and clusters, and in certain portions, we found cells anchoring and foraging the root hairs. Importantly, we did not observe cells colonizing inside parts of the roots under our tested seawater growth conditions. A similar finding was reported in a terrestrial halotolerant PGPR *Azospirillum halopraeferens*, where the colonization was observed more on the root surfaces of black mangrove seedlings under seawater growth conditions [53].

#### Metagenomic survey of L1I39^T^-specific OTUs in brackish-associated rice varieties

Our attempts to re-isolate L1I39^T^-like strains from the pokkali rice using different cultivation methods were unsuccessful. Therefore, we determined the L1I39^T^ prevalence by mapping its full-length 16S rRNA gene sequence against the 16S rRNA amplicon metagenomic datasets of two native salt-tolerant rice cultivars collected from brackish environments of central and northern regions of Kerala. This analysis revealed limited operational taxonomic units (OTUs) having a read length of 400 bp sharing > 99% 16S rRNA sequence similarities with L1I39^T^ from both rice cultivars (Table 2), suggesting its occurrence is very low abundance in those ecosystems. Importantly, we noticed that L1I39^T^-specific OTUs represented more in the roots when compared to the rhizosphere samples where we found, except in one rhizosphere sample, all other rhizosphere samples were negative (Table 2). Moreover, these findings provided us with essential information on L1I39^T^ that 1) it has a preferential colonization of the roots over the rhizosphere region, thereby signifying its close relationship with the plant host, 2) it is an integral part of the root-associated indigenous microbiota of brackish-associated rice varieties and 3) it occurs with a low abundance. Studies have shown that bacterial taxa of low-abundance populations in a habitat can form the most physiologically significant symbiotic interactions [54] and, in some instances, fulfill essential environmental functions, for example, nitrogen fixation by diazotrophic bacteria in the ocean [55]. Based on the findings of in-vitro PGPR traits and in-planta *nifH* expression and growth experiments, we hypothesize that L1I39^T^ might play a similar significant functional role in pokkali rice growth and health in nitrogen-poor brackish environments despite their low abundance, thus, conferring L1I39^T^ as a rare important PGPR taxon.

**Table 2:** List of L1I39^T^ OTUs recovered from 16S rRNA amplicon metagenomics datasets of brackish-associated rice varieties of central and northern coastal regions of Kerala

Samples	BioProject No	BioSample No	Rhizosphere	Root
Pokkali	PRJNA970257	SAMN35343998	0	2
		SAMN35344334	0	1
		SAMN35344587	0	1
Kaipad	PRJNA970551	SAMN36510157	1	0
		SAMN36766283	0	9
		SAMN36766609	0	2
		SAMN36766632	0	3

#### General genome features

**Fig. 5:**
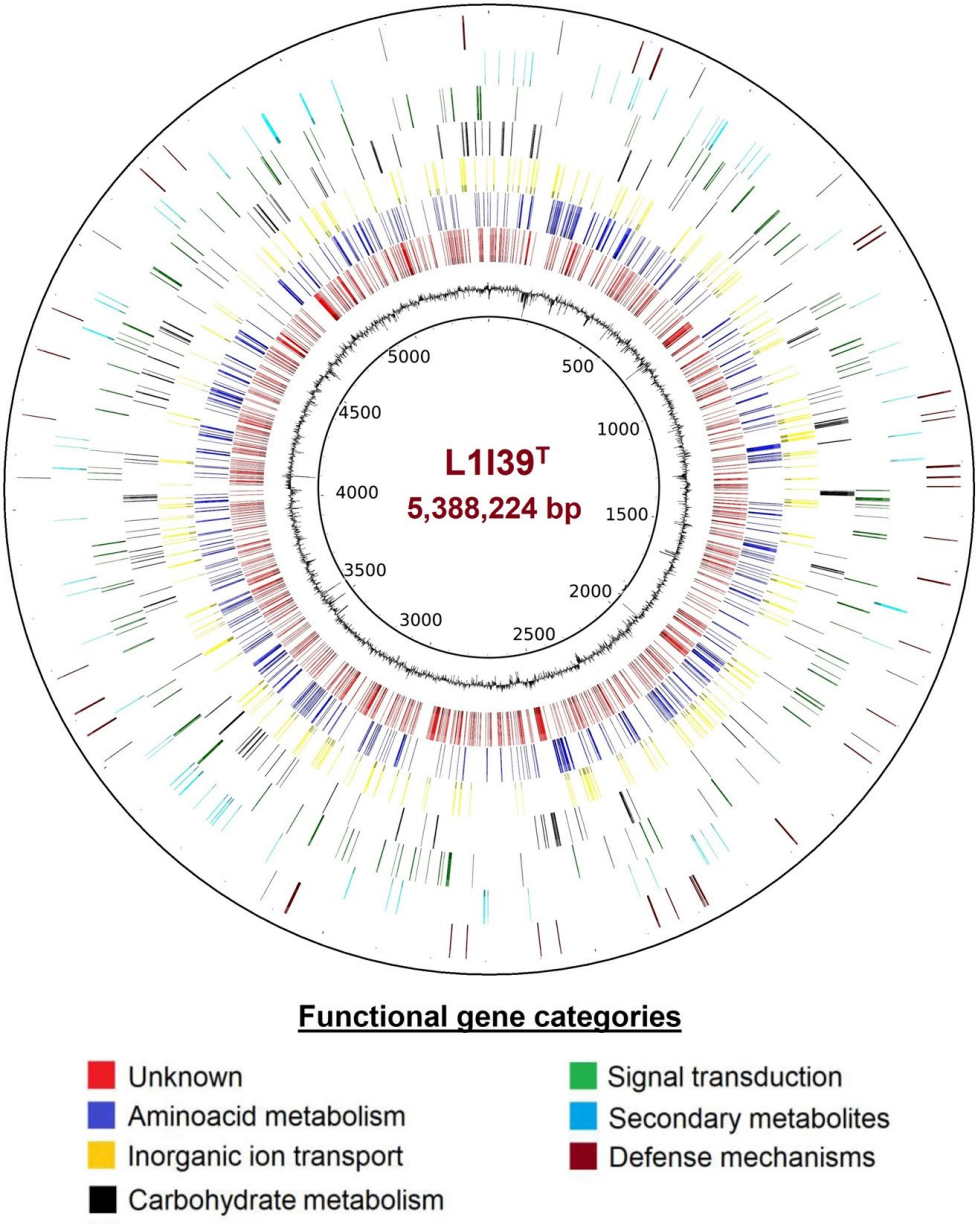
A single circular genome representation of L1I39^T^. The inner circle represents the genome size, the second circle represents the GC content, and the following circles represent coding sequences of different functional gene categories, as indicated by different colors

The L1I39^T^ genome comprises a single circular chromosome of 5,388,224 bp with no extra plasmids and a GC content of 66.9% (Fig. 5). Genome quality estimation determined using checkM showed that the assembled genome is of high quality with 99.65% completeness with no contamination and strain heterogeneity. The general genome features of the L1I39^T^ and its closest phylogenetic neighbors, *A. cavernae* Sn-9-2^ T^ and *A. spiritensis* DSM 9035^ T^, were summarized in Table S5.

Next, we investigated the genomic composition of L1I39^T^ to relate its specific adaptations to plant-associated lifestyles and brackish tidal environments. For this purpose, we analyzed the SEED subsystem features available in the RAST server [32]. This analysis revealed that a high proportion of protein-coding genes were assigned to the functional category amino acids and derivatives (631), followed by carbohydrates (506). In contrast, genes listed under the protein metabolism (255) were less abundant, which points to a specialized adaptation of L1I39^T^ to various plant-derived substrate metabolism. Notably, several protein-coding genes were categorized under the membrane transport (336), indicating the ability of L1I39^T^ to sense and respond to various environmental stimuli, including host-derived nutrients and tolerance to different abiotic stress, including salinity. An overview of the SEED subsystem categories identified in the genomes of L1I39^T^, *A. cavernae* Sn-9-2^ T^, and *A. spiritensis* DSM 9035^ T^ were listed in Table S6.

#### Functions related to plant-associated lifestyle

##### Motility, chemotaxis, and biofilm

The plant colonization traits such as motility, chemotaxis, and biofilm formation are essential prerequisites for potential PGPR to interact with their hosts efficiently. The L1I39^T^ genome encodes a complete set of genes related to flagellar motility and chemotaxis organized into a single large operon of 42.15 kb size comprising 44 genes (Table S7). Importantly, L1I39^T^ is negative for motility under our tested phenotypic conditions. This disagreement between the presence of genes encoding for flagellar motility and the non-motile phenotype indicates tight regulation of motility in L1I39^T^. Similar discrepancies in non-motile phenotype and genome information were observed in other plant-associated endophytic bacteria [56–58].

Further genome analysis identified 11 methyl-accepting chemotaxis proteins (MCP) with one MCP (WP_209103394.1) positioned within the flagellar-chemotaxis gene cluster. This MCP gene shared 68.04% amino acid identity with the transmembrane chemoreceptor *tlpA1* gene (AZC_0660) of *Azorhizobium caulinodans* ORS 571^ T^. This chemoreceptor gene was identified as critical for *A. caulinodans* ORS 571^ T^ host colonization, where the *tlpA1* mutant strain was severely impaired with chemotaxis and competitive root colonization [59]. Likewise, the *tlpA1* gene homolog in the L1I39^T^ genome might indicate a similar role in host colonization. However, the number of MCP genes identified in the L1I39^T^ genome is less compared to the *A. caulinodans* ORS 571^ T^ genome, which contains 43 MCP genes [60]. The non-motile phenotype and a low number of MCP genes in L1I39^T^ indicate a lifestyle confined to a specific environmental niche.

The strain LI139^T^ is positive for biofilm formation (Fig. S6a) and a strong attachment of pokkali rice root seedlings under 20% seawater conditions was observed (Fig. S6b). Hence, we searched for putative genes and gene clusters critical for host tissue attachment and establishment. The L1I39^T^ genome carries four genes organized as a single cluster participating in lipopolysaccharide-o-antigen biosynthesis. These genes include dTDP-4-dehydrorhamnose 3,5-epimerase (*rfbC*, WP_209104488.1), dTDP-glucose 4,6-dehydratase (*rfbB*, WP_209104489.1), dTDP-dehydrorhamonase reductase (*rfbD*, WP_209105319.1) and glucose-1-phosphate thymidylyltransferase (*rfbA*, WP_209104490.1). Also, genes coding for lipopolysaccharide (LPS) biosynthesis, including LPS heposyltransferase II (*waaF*, WP_209100376.1), LPS heposyltransferase I (*waaC*, WP_209100378.1), ADP-glyceromanno-heptose 6-epimerase (*rfaD*, WP_209100380.1) and D-glycero-beta-D-manno-heptose-7-phosphate kinase (*rfaE1*, WP_247657679.1) were present. Additionally, we identified two genes coding for prepilin peptidase (*tadV*, WP_209101036.1) and type IVb pilin (*flp,* WP_209101034.1) as part of the tight adherence (tad) locus. Also, genes for exopolysaccharide biosynthesis (*exoD*, WP_247657663.1), LPS assembly protein (*lapA*, WP_247657749.1), and putative surface layer protein (*sapC*, WP_247658490.1) were present. Further analysis revealed a symbiosis polysaccharide locus (*syp*) consisting of 18 genes spanning around 16.55 kb in size (Fig. S6c). Studies have shown that this *syp* locus is essential for bacteria host attachment and colonization [61]. Therefore, L1I39^T^ might use these genes, including the *syp* locus, to efficiently attach and colonize the host.

##### Rhizosphere oxidative stress (ROS) detoxification

To establish an efficient colonization event, L1I39^T^ must overcome the rhizosphere oxidative stress (reactive oxygen species, ROS) induced by the host plant [62]. Experimentally, we show that L1I39^T^ can tolerate hydrogen peroxide up to 5 mM (Fig. S7). Accordingly, the L1I39^T^ genome encodes multiple genes, including superoxide dismutase, catalase/peroxidase HPI (*katG*), catalase, alkyl hydroxy peroxide reductase, thioredoxin reductase, thiol peroxidase, non-heme chloroperoxidase, glutathione S-transferase, glutathione reductase (*gorA*), glutaredoxin, OsmC family protein, organic hydroperoxide resistance protein, and peroxiredoxin (Table S8), responsible for detoxifying the exogenous ROS resulting from plant interactions [63]. Also, genes that regulate ROS detoxification, including two copies of hydrogen peroxide-inducible activator, were identified. These results indicate the significant ability of L1I39^T^ to detoxify exogenous ROS, which may affect several aspects of plant–microbe interactions, including plant growth and health.

##### Energy metabolism

The strain L1I39^T^ is capable of aerobic respiration and encodes a complete set of genes required for oxidative phosphorylation (Table S9). These include NADH-quinone oxidoreductase (*nuo* genes, complex I), succinate dehydrogenase (*sdh* genes, complex II), and three-terminal respiratory oxidases belonging to a) cbb3-type cytochrome c oxidases (*ccoNOQPGS* operon), b) aa3-type cytochrome c oxidase (*ctaD*, *coxB*, *coxC*, and *coxIV*), and c) bd-type quinol oxidases; cytochrome o ubiquinol oxidase (*cyoA*, *cyoBCD*), and cytochrome d ubiquinol oxidase (bd I complex; *cydABX* operon and two copies of bd II complex; *appC*, *cydB*). Notably, these aerobic terminal oxidases have different oxygen affinities and are differentially regulated depending on the availability of varying oxygen concentrations in the growth habitat. For instance, cbb3-type cytochrome c oxidases and bd-type quinol oxidases have a high oxygen affinity critical for microaerobic respiration and nitrogen fixation [64, 65]. In contrast, aa3-type cytochrome c oxidases have a low oxygen affinity, predominantly expressed under aerobic growth conditions [66].

In addition to multiple terminal oxidases, the L1I39^T^ genome encodes a putative dissimilatory nitrate reductase operon (*narGHJI*), class I fumarate hydratase (*fumA*), class II fumarate hydratase (*fumC*, two copies), formate dehydrogenase with four subunits, L-lactate utilization protein (*lutB*), LUD domain protein, L-lactate dehydrogenase (*lldD*) and its regulator (*lldR*) (Table S10). These genes may confer a metabolic growth advantage under low-oxygen conditions, with formate, lactate, and fumarate as electron donors and nitrate as an electron acceptor. Together, these findings suggest the significant genetic ability of L1I39^T^ to adapt and thrive in water-logged brackish pokkali rice fields.

##### Central carbon metabolism

The L1I39^T^ genome encodes a complete set of genes required for a functional pentose phosphate pathway and the Entner-Doudorff pathway (Table S11). However, it lacks a key regulatory enzyme of the glycolysis pathway (6-phosphofructokinase) and two rate-limiting enzymes of the gluconeogenesis pathway (phosphoglucoisomerase and glucose-6-phosphatase), indicating that glycolysis and gluconeogenesis pathways are incomplete. Although enzymes catalyzing the remaining steps of the glycolysis and gluconeogenesis pathways were present (Table S11), suggesting that carbohydrate metabolism is carried out through the Entner-Doudorff and pentose phosphate pathways, indicating that L1I39^T^ may depend on the host for carbohydrate precursors. For oxidation of pyruvate to acetyl-coenzyme, the L1I39^T^ genome encodes a three-component pyruvate dehydrogenase complex (two E1 component subunits, alpha and beta, and a dihydrolipoamide S-acetyltransferase). Further, we identified genes coding for enzymes catalyzing all eight essential steps of the complete citric acid cycle (Table S11). Also, the presence of citrate lyase (three copies) and a malate synthase indicate an active glyoxylate shunt pathway. Together, these genomic data suggest that L1I39^T^ is likely to prefer dicarboxylates (metabolized via a critic acid cycle) as the source of energy for its growth and other metabolic functions rather than carbohydrates. In support, we show experimentally through phenotypic growth assays that L1I39^T^ could grow well on dicarboxylates instead of carbohydrates (Fig. S8 & Table S12). Similar findings were reported in several well-characterized diazotrophic PGPR strains, notably of the genera *Azospirillum*, *Azoarcus*, and *Herbaspirillum,* which are often unable to utilize carbohydrates [67].

In addition, the L1I39^T^ genome encodes > 600 transporter-linked genes, comprising about 15.59% of all the genes. The highest number of genes annotated to ATP binding cassette (ABC) type transporter (365 genes), which corresponds to 46% of the total transporter genes, 93 genes (12%) belong to Tripartite ATP-independent periplasmic (TRAP) transporters, including dicarboxylate and tricarboxylate, 63 genes (8%) belong to branched-chain amino acids transporter genes, and 46 genes (6%) belong to MFS transporters genes (Table S13). Moreover, the presence of a higher number of transporters than its related phylogenetic neighbors (Table S13), indicates its potential genetic ability to uptake and utilize diverse substrates. We also found genes involved in the degradation of various aromatic compounds through 3-oxoadipate and phenylacetate degradation pathways (Table S14).

##### Vitamins biosynthesis

Vitamins are essential growth cofactors required for several enzymatic processes in central metabolism. In addition, vitamins can also play a crucial role in maintaining the symbiotic status of two different organisms. For instance, bacterial symbionts capable of synthesizing vitamins can become key symbiotic partners with eukaryotic hosts, including legume-rhizobia symbiosis [68]. The L1I39^T^ genome encodes all essential genes involved in the biosynthesis of multiple vitamins (Fig. S9 & Table S15), suggesting its genetic capability to synthesize necessary growth factors for growth. In support, we experimentally confirm that L1I39^T^ can grow in a defined vitamin-free mineral salts medium without dependence on the external supply of vitamins under our tested conditions even after repeated subcultures (data not shown).

Several studies have shown that vitamin auxotrophs of diverse PGPR failed to establish an effective symbiosis with their respective plant hosts. For example, thiamine auxotrophic mutants a) PCL1079 of *Pseudomonas fluorescens* WCS 365 lost their ability to colonize the tomato roots [69] and b) Ao5-E01 and Ao55-D02 of *A*. *caulinodans* ORS 571^ T^ formed defective nodules with reduced nitrogen fixation [70]. Likewise, riboflavin auxotrophic mutant of *Rhizobium leguminosarum* bv. *trifolii* strain T1 formed ineffective root nodules in clover [71], and the biotin auxotrophic mutant of *Sinorhizobium meliloti* strain 1021 showed poor alfalfa root colonization [72]. Based on these findings, we hypothesize that L1I39^T^ would utilize these vitamin biosynthetic gene clusters for effective host interactions rather than supply vitamins to its host, which is unlikely.

##### Sulfur, phosphate, and nitrogen-assimilation

Plant-associated bacteria can assimilate sulfonates and sulfur esters as organic sulfur and sulfates as inorganic sulfur from the environments [73]. In the L1I39^T^ genome, genes primarily involved in the metabolism of sulfonates were absent. However, a single-copy gene encoding aliphatic sulfonate ABC transporter substrate-binding protein (*ssuA*) was present. We also identified genes coding for reducing sulfur esters, including a sulfatase-like hydrolase and two copies of arylsulfatases. Additional analysis identified genes for the assimilation and transport of sulfates (Table S16). However, the homolog of *cysB*, the primary regulator of sulfate metabolism, was absent. Further, genes that participate in the assimilation and export of sulfite, including the three copies of sulfite reductase and seven copies of sulfite exporter TauE/SafE family proteins (Table S16), were identified. The putative genes that catalyze the oxidation of sulfide to sulfate were absent, but a gene coding for sulfite oxidase was present.

Phosphonate is considered one of the rich sources of phosphates in the soil. Microbes use a phosphonate-related gene system (*phn*) to break down phosphonates in the environments to release phosphates efficiently. Accordingly, the L1I39^T^ genome encodes a complete gene cluster (*phnNLKAFBJIHGFM* genes) responsible for phosphonate degradation into phosphate (Table S17). In addition to this, we also identified two potential high-affinity phosphate transport systems crucial for phosphate mobilization, including *pstBACS* (transport) *and phoUB* (regulator) gene clusters for inorganic phosphates and *phnE1E2DC* for phosphonates (Table S17). These results suggest L1I39^T^ might depend on these systems to mobilize inaccessible phosphorous during phosphate limitations, as observed in other plant-associated bacteria [74]. Further analysis identified potential enzymes, such as polyphosphate kinase and exopolyphosphatase, indicating that L1I39^T^ likely stores energy and phosphorus in the form of polyphosphate.

In addition to examining the genome for sulfur and phosphate assimilation, we also identified several potential genes involved in the assimilation of nitrogen, including genes coding for ammonium transporters, reductases for nitrate and nitrite, regulators that sense nitrogen status, glutamate dehydrogenase, and a functional glutamine synthetase/glutamate synthase (GS/GOGAT) system as listed in (Table S18). In addition, the L1I39^T^ genome possesses genes coding for the uptake and hydrolysis of urea, including a urease operon (*ureABCEFGD*) and the urea transport systems (*urtEDC* and *urtBA*) and a functional urea carboxylase/allophanate hydrolyase pathway, suggesting its ability to degrade urea into ammonia (Table S19).

##### Iron acquisition

The L1I39^T^ genome lacks genes that participate in the biosynthesis and secretion of siderophores. In support, we experimentally prove that L1I39^T^ is negative for siderophore production (data not shown). However, the L1I39^T^ contains dedicated transport systems to capture and uptake external iron, including TonB-dependent siderophore receptor proteins, siderophore-interacting proteins, iron ABC transport substrate-binding proteins, iron ABC transport permease, iron ABC transport ATP-binding proteins, ferric hydroxamate ABC transport permease (*fhuB*), and siderophore-iron reductase (*fhuF*). Importantly, these genes are organized into different gene clusters (Table S20).

Further investigations revealed genes encoding for ferrochelatase (*hemH*), TonB-dependent hemoglobin/transferrin/lactoferrin family proteins, biliverdin-producing heme oxygenase, heme ABC transporter-ATP binding protein, and hemin uptake protein (*hemP*), responsible for acquiring host iron. Also, genes coding for iron storage proteins that include ferritin, bacterioferritin (bfr), and two copies of ferritin-like domain-containing proteins and fur-type iron response transcriptional regulator (three copies) were present. Based on these genome findings, we hypothesize that L1I39^T^ cells may depend on siderophores produced by other root-inhabiting rhizobacteria and host heme to satisfy their need for iron, a similar feature documented in well-characterized diazotrophic PGPR strains such as *Pseudomonas stutzeri* A1051 [75] and *Azoarcus* sp. BH72 [76].

#### Plant growth functions

##### Nitrogen fixation and hydrogen metabolism

**Fig. 6:**
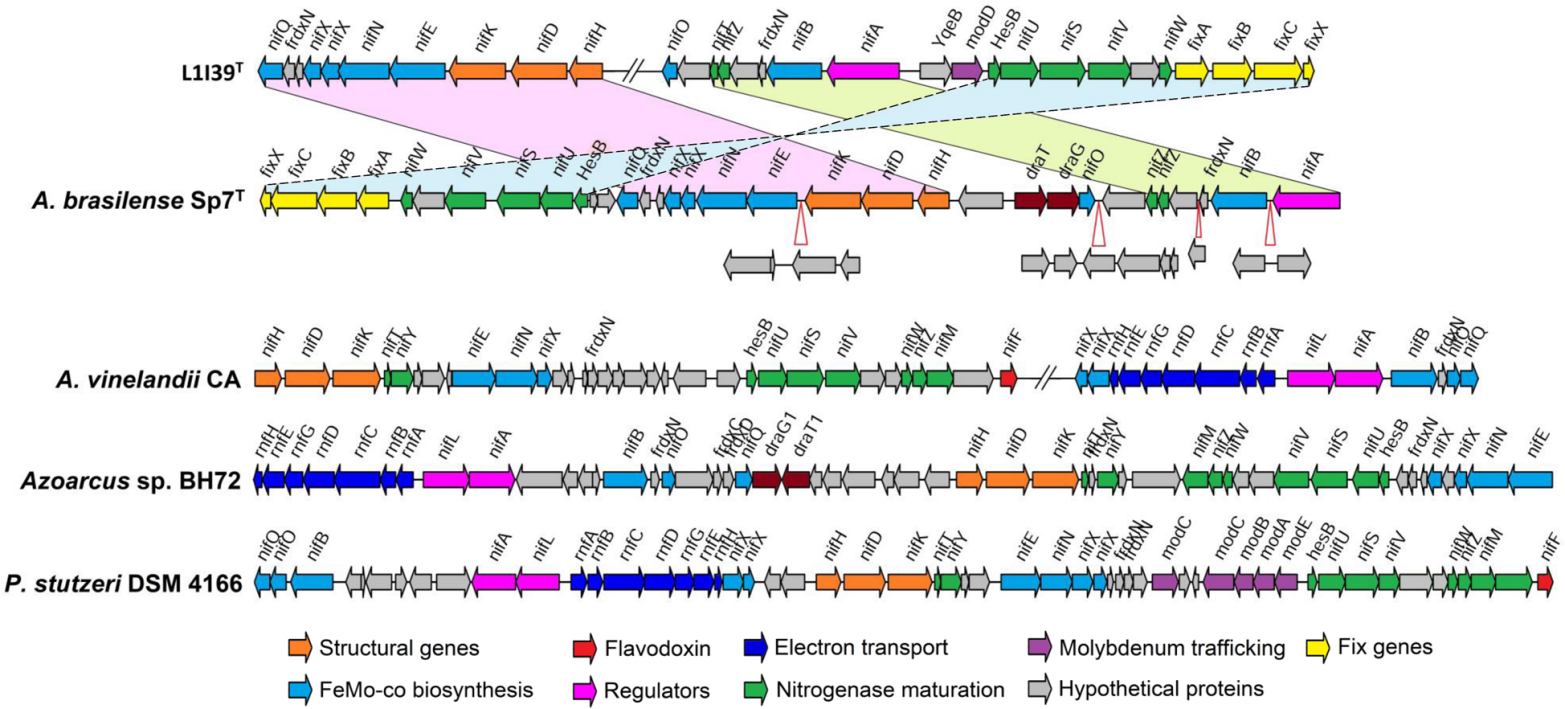
The nitrogen fixation (nif) gene cluster comparison between L1I39^T^ and four well-characterized rice-associated diazotrophs: *A*. *brasilense* Sp7^T^, *A*. *vinelandii* CA, and *Azoarcus* sp. BH72 and *P. stutzeri* DSM 4166. Red open sharp triangles indicate the location of additional genes. Highlighted regions with continuous border lines indicate gene synteny oriented in the same direction, whereas highlighted regions with dotted lines indicate the gene synteny oriented in the opposite direction

The L1I39^T^ genome encodes all necessary genes required for nitrogen fixation (*nif*), organized in a 29.2 kb large *nif* region consisting of 32 genes distributed non-contiguously across two different loci (Fig. 6). The GC content of this *nif* region is similar to the average GC content of the whole genome (66.3% vs. 66.9%), suggesting that this *nif* region is not part of a genomic island that has been acquired recently through horizontal gene transfer, as it was observed in *Pseudomonas stutzeri* [75]. The nitrogenase system of L1I39^T^ constitutes the molybdenum-iron (MoFe) type nitrogenase encoded by *nifDK* and an iron (Fe) protein encoded by *nifH*. Genes necessary for FeMo-co biosynthesis (*nifB*, *nifQ*, *nifENX*, and *nifUSV*), nitrogenase maturation (*nifM*, *nifZ*), electron transfer flavoproteins (*fixABCX*), and transcriptional regulation of *nif* operon (*nifA*) were present. Other essential genes likely to be involved in the regulatory cascade of nitrogen fixation are shown in Table S18. No genes coding for alternative vanadium (VFe) or iron only (FeFe)-dependent nitrogenase systems were present.

A NifHDK phylogenetic analysis showed that the nitrogenase system of L1I39^T^ is closely related to *A. cavernae* Sn-9-2^ T^ (Fig. S10). However, it is distantly related to the nitrogenase system of *A. spiritensis* DSM 9035^ T^ and other nitrogen-fixing members of the genera *Xanthobacter* and *Azorhizobium* (Fig. S10). Moreover, the *nif* gene cluster of L1I39^T^ and *A. cavernae* Sn-9-2^ T^ shared high gene synteny compared to *A. spiritensis* DSM 9035^ T^, *A*. *caulinodans* ORS 571^ T^, and *Xanthobacter autotrophicus* Py2 (Fig. S11). These results suggest that the nitrogenase system of L1I39^T^ and *A. cavernae* Sn-9-2^ T^ might have been acquired from a common ancestor but are evolutionary different from that of other members of the family *Xanthobacteraceae*.

We then compared the *nif* gene organization of L1I39^T^ against the four other well-studied diazotrophs associated with rice plants: *Azospirillum brasilense* Sp7^T^, *Azotobacter vinelandii* CA, *Azoarcus* sp. BH72, and *P. stutzeri* 4166. This analysis revealed that the *nif* region of L1I39^T^ shared a relatively close similarity to *A. brasilense* Sp7^T^ than with other diazotrophs (Fig. 6). For instance, In *A. brasilense* Sp7^T^, the *nif* region that contained a set of genes (*nifQ frdxN nifXNEKDH*) were contiguous and arranged in the same order as observed in L1I39^T^, except a few additional protein-coding sequences were found in between the genes of *nifQ frdxN nifXNE and nifKDH* (Fig. 6). Similarly, the region encoding genes for *nifUSVW* and *fixABCX* were organized very similar to L1I39^T^, except this region is flipped (Fig. 6). In addition to this, an electron transport system (*rnfABCDGEF* operon) and NifL, a negative regulator of the nitrogenase system, were absent in both L1I39^T^ and *A. brasilense* Sp7^T^ but were present in *A*. *vinelandii* CA, *Azoarcus* sp. BH72, and *P*. *stutzeri* 4166. Importantly, genes encoding for DraT/DraG-mediated posttranslational regulation of nitrogenase by ADP-ribosylation were absent in L1I39^T^, suggesting a nitrogenase regulation mechanism different from that reported in *A*. *brasilense* Sp7^T^ and *Azoarcus sp*. BH72.

During the nitrogen fixation process, hydrogen (H_2_) gas is released as a by-product of the nitrogenase reaction, making the nitrogen fixation process less efficient. However, the oxidation of H_2_ by the hydrogenase enzyme improves the efficiency of the nitrogen fixation process. Hence, to fix nitrogen more efficiently, the L1I39^T^ genome encodes a complete set of genes required for H_2_ metabolism distributed along a 19.7 kb cluster comprising 19 potential genes (Fig. S12 & Table S21), arranged in the order of *hupTUV*, *hypF*, *hupSLCDFGHJK*, *hypAB*, *hupR,* and *hypCDE*. This cluster closely resembles the H_2_ uptake genetic system of *A*. *caulinodans* ORS 571^ T^, except it does not contain the *hupE* gene coding for nickel transporter. Notably, the H_2_ uptake genetic system is absent in the genomes of *A*. *cavernae* Sn-9-2^ T^ and *A*. *spiritensis* DSM 9035^ T^.

##### ACC deaminase production

The L1I39^T^ genome encodes a copy of the ACC deaminase (*acdS*) gene (WP_209104892.1) and a leucine-responsive regulatory protein (Lrp, WP_209104891.1) known to regulate the *acdS* gene expression were identified. The AcdS and Lrp proteins shared relatively low similarity (71.73 and 49.66%, respectively) with well-characterized ACC deaminase-producing PGPR, *Paraburkholderia phytofirmans* PsJN^T^. No transposable or mobile genetic elements nearer or to the flanking regions of the *acdS* gene and its regulatory protein were present, indicating that this gene is stably present in the L1I39^T^ genome. Further, this data potentially suggests that this gene could have been acquired positively through a long-term evolution while interacting with its plant hosts in a brackish environment, where the expression of the *acdS* gene plays a valuable and positive plant function. In support of this view, the *acdS* gene was absent in the genomes of *A*. *cavernae* Sn-9-2^ T^ and *A. spiritensis* DSM 9035^ T^ (Table S13). Studies have shown that PGPR expressing ACC deaminase promotes plant growth under various stressful conditions, including salinity [77]. Similar to these findings, we show that L1I39^T^ promotes the pokkali rice growth under a tested nitrogen-deficient seawater condition, indicating that the *acds* gene could be functional and can play a significant contributory role in pokkali rice growth and fitness in those stressful nitrogen-poor brackish growth environments.

##### Other PGP traits

The L1I39^T^ genome also encodes genes for acetoin and 2,3-butanediol production (Table S22), well-characterized volatile compounds known to promote plant growth [78].

#### Plant interactions

##### Secretion systems

Protein secretion systems play a significant role in plant–microbe and environmental interactions. In the L1I39^T^ genome, genes coding for different membrane proteins of the general secretion (Sec)-mediated pathway, including SecA (ATPase), SecY, SecE, SecG, SecDF-YajC (forming a putative protein complex), YidC, and SecB (export chaperone) were identified. Likewise, we also found two putative signal recognition particle (SRP)-binding proteins (FtsY and FfH). These findings suggest that LI139^T^ may use the SRP and the Sec-mediated pathways for optimal translocation of proteins [79]. Additional analysis revealed the presence of a twin-arginine protein export (TatABC) complete system that delivers folded proteins across the cytoplasmic cell membrane, unlike the Sec-mediated pathway that translocates unfolded proteins. In addition to Sec and Tat-mediated protein translocating systems, the L1I39^T^ genome also contains dedicated secretion systems, including the Types I (T1SS), IV (T4SS), and VI (T6SS) as well as a specific subset of effector molecules, suggesting that L1I39^T^ can modulate host cellular functions as well as mediate host-specific interactions.

T1SS is a simple protein secretory apparatus that translocates a variety of proteins, including toxins, lipases, proteases, exopolysaccharides, and siderophores across the bacterial cell envelope into the external environment in a single step and plays a vital role in host interactions [80]. The L1I39^T^ genome encodes all necessary genes as part of a functional T1SS (Table S23). Also, several membrane fusion proteins were found as part of the multidrug efflux resistance transport systems, suggesting a possible functional role in stress tolerance. Notably, we identified a single gene cluster (from WP_209105009.1 to WP_209105014.1) consisting of four genes, namely a type I secretion membrane fusion protein, a T1SS permease/ATPase, a putative glycosyltransferase and a putative surface layer protein (SapC), is probably involved in the secretion of exopolysaccharide necessary for the host attachment and biofilm formation.

T4SS is a versatile multiprotein translocation system involved in genetic exchange and the delivery of host-specific effector proteins or DNA–protein complexes directly into target prokaryotic or eukaryotic cells that may aid in establishing host interactions, including symbiosis or pathogenesis [81]. Most T4SSs described comprised a minimum of core 12 proteins (VirB1 to VirB11 and VirD4). The L1I39^T^ genome encodes a complete set of VirB1 to VirB10 proteins along with VirD4 and VirD2 (Fig. S13 & Table S24), indicating that this T4SS is part of a conjugal transfer system. However, OriT and VirE were absent, suggesting that this system might not transfer DNA into the eukaryotic hosts [82]. Also, the homologs of T4SS effector proteins, VirJ, VirF, VirE1, VirE2, and VirD5, were absent. These findings suggest that T4SS might participate in host interactions rather than a conjugal transfer of DNA or any effector proteins.

Further genome investigations identified two different gene clusters comprising a minimum of 13 core subunits required to form a functional type 6 secretory apparatus (Fig. S14a & Table S25). Studies have shown that T6SS plays a central role in interbacterial interactions, where Gram-negative bacterial cells use this delivery system to kill other competing bacteria to gain fitness in their growth habitat, and this inhibition of cells requires cell–cell contact [83]. We checked whether this was true for L1I39^T^, and an in vitro contact-dependent growth inhibition assay was performed. This experiment showed a negative result for L1I39^T^ (Fig. S14b), indicating that this system may not be involved in interbacterial competition but can mediate host-specific interactions. A similar finding was reported in *A. caulinodans* ORS 571^ T^*,* where T6SS is not engaged in interbacterial competition but is identified as important for its symbiotic interactions with the host [84].

In addition to the secretion systems, the L1I39^T^ genome carries two copies of N-acyl homoserine lactonase (WP_209105105.1 and WP_209102178.1), a metalloenzyme that degrades bacterial quorum sensing (QS) signals. The genes involved in the N-acyl homoserine lactone (AHL)-mediated QS signaling, LuxI/LuxS-type autoinducer synthetase, and LuxR-type autoinducer-response regulator were absent. Therefore, the presence of AHL lactonase indicates that L1I39^T^ can inactivate the AHL-based QS signaling molecules produced by other competing rhizobacteria, thereby disrupting their coordinated cellular functions, including biofilm formation, and thus can have a competitive advantage during the host root colonization. A similar finding has been reported in AHL lactonase-producing *Azospirillum brasilense* Az39, where degradation of AHL signaling compounds improves the root colonization competitiveness of Az39 [85]. Notably, this enzyme was absent in the genome of *A*. *cavernae* Sn-9-2^ T^.

Further, we identify several potential eukaryotic-like interactive proteins encoded in the L1I39^T^ genome (Table S26), which may participate in the host interactions, including the plant defense system evasion [86].

### Brackish adaptation

Ecophysiological data suggests optimal cell growth, root colonization, and plant growth promotion of L1I39^T^ are greatly enhanced by the 20% seawater condition, implying that L1I39^T^ might rely on sodium ions for energy generation to perform cellular functions, as observed in many marine bacteria [87]. However, our genome analysis indicated that the L1I39^T^ genome has an incomplete sodium-dependent energy cycle. Since the genes encoding for Na^+^ translocating NADH: ubiquinone oxidoreductase (Na^+^NQR) and Na^+^ transporting ATPase were absent. Nevertheless, the L1I39^T^ genome encodes several sodium transporters (Table S27), including a Na^+^ dependent transporter, multiple Na^+^/H^+^ antiporter subunits, Na^+^/Pi cotransporter family, Na^+^/bile acid symporter, Na^+^/alanine symporter, Na^+^/glutamate symporter, Na^+^/Pi symporter, Na^+^/dicarboxylate symporter and Na^+^ translocating pyrophosphatase. It is well known that Na^+^ pumps, especially Na^+^/H^+^ antiporters, play an essential role in sodium homeostasis [88]. Therefore, L1I39^T^ might depend on these Na^+^ pumps to export excess toxic Na^+^ and import H^+^ when the external sodium levels are too high, thereby maintaining cellular osmotic homeostasis and tolerating hyperosmotic stress. Notably, fewer genes coding for sodium transporters were identified in the genomes of *A. cavernae* Sn-9-2^ T^ and *A. spiritensis* DSM 9035^ T^ compared to the L1I39^T^ genome (Table S13).

Bacterial cells also accumulate more intracellular cations of K^+^ as an immediate transient response to high salinity stress [89]. The presence of genes encoding for three putative potassium uptake systems, including TrK, Kdp, and Kup (Table S27), could indicate the potential genetic capability of L1I39^T^ to use these systems for hyperosmotic adaptation. This view agrees with the earlier observations that *Sinorhizobium meliloti* carrying single or double mutations in TrK and Kup systems lost their ability to grow at high salinity [90]. Additional analysis identified genes coding for magnesium transporters, two copies of *mgtE,* and a *CorA* responsible for maintaining ionic homeostasis under salt stress [91].

In addition to the Na^+^ extrusion and inorganic solute uptake mechanisms, bacteria can synthesize and accumulate diverse organic compatible solutes, which improves their ability to cope with high salinity stress [92, 93]. The L1I39^T^ genome encodes genes for trehalose biosynthesis, including maltose alpha-D-glucosyltransferase (*treS*), trehalose-6-phosphate synthase (*otsA*), trehalose-phosphatase (*otsB*), malto-oligosyltrehalose synthase (*treY*), and malto-oligosyltrehalose trehalohydrolase (*treZ*). In addition, genes coding for glutamate synthesis, including glutamate dehydrogenase (*gdh*), glutamine synthetase (*glnA*), and glutamate synthase alpha and beta subunits (*gtlBD*), were present. Further analysis identified genes coding for enzymes that convert glutamate to proline, an important osmoprotectant. These enzymes include glutamate-5-semialdehyde dehydrogenase (ProA), glutamate-5-kinase (ProB), and pyrroline-5-carboxylate reductase (ProC). Also, one trifunctional transcriptional regulator/proline dehydrogenase/ pyrroline-5-carboxylate dehydrogenase (*putA*) was present. Putative genes coding for synthesizing betaine, choline, ecotine, hydroxyectoine, and dipeptide N-acetylglutaminylglutamine amide (NAGGN) were absent. However, a betaine/carnitine/choline (BCCT) transporter and N-acetylglutaminylglutamine amidotransferase (*asnO*) was present. These genome findings suggest that L1I39^T^ may depend on these genes (Table S27) for survival and growth in brackish environments.

## Conclusions

**Fig. 7:**
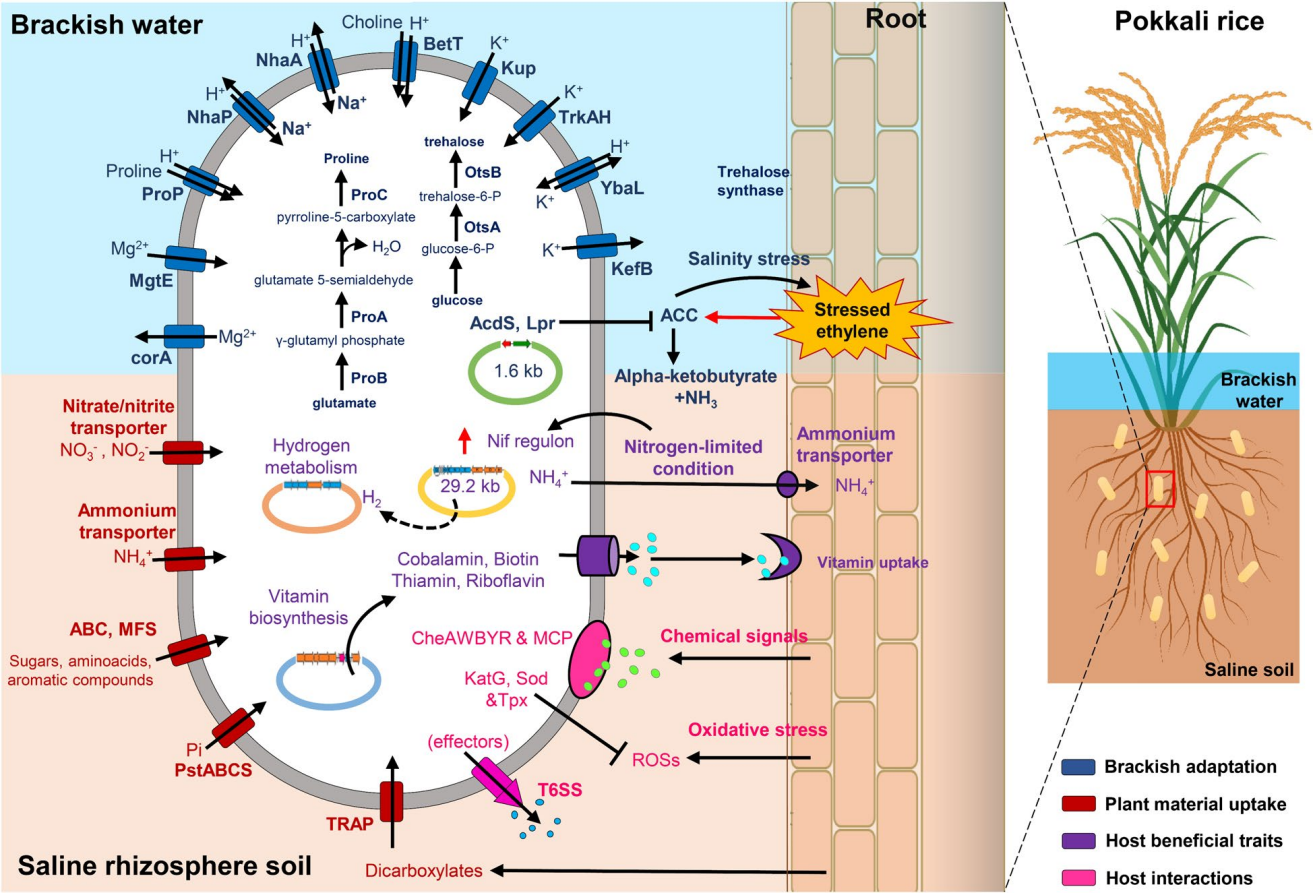
Schematic Figure representing the genetic determinants of L1I39^T^ critical for plant-associated lifestyle, plant growth functions, and brackish environmental adaptations

We isolated and functionally characterized a novel *Aquabacter* strain L1I39^T^ from the rhizosphere region of a native salt-tolerant pokkali rice cultivated in water-logged brackish tidal environments of central Kerala, India. The strain L1I39^T^ possesses multiple PGP properties, including biological nitrogen fixation and ACC deaminase production, and has the ability to promote pokkali rice growth in nitrogen-limiting seawater conditions. More importantly, optimal cell growth, rice root colonization, and plant growth promotion of L1I39^T^ are greatly influenced by the presence of a 20% seawater condition compared to a zero-seawater condition, suggesting that a brackish environmental condition is essential for L1I39^T^-pokkali rice symbiosis. In addition, the ability to tolerate 3% NaCl, positive growth in ZoBell marine agar, and optimal growth at 20% seawater concentration indicates that L1I39^T^ is physiologically well-adapted to its brackish growth habitat. Detailed genome characterization of L1I39^T^ revealed an array of genetic traits as specific adaptations to plant-associated lifestyles and brackish environments (Fig. 7). The 16S rRNA amplicon sequence analysis identified L1I39^T^ as part of the root-associated indigenous microbiota of brackish-associated native rice varieties but with a low abundance. Overall, the findings of this study show that L1I39^T^ is an important rare PGPR taxon that would play a significant functional role in pokkali rice growth and health in nitrogen-limiting brackish environments, which needs further investigation. Finally, we establish L1I39^T^ as a novel *Aquabacter* species by following a polyphasic taxonomy approach and named *Aquabacter pokkalii* sp.nov., (LMG 28413^ T^ = MCC 4526^ T^).

### Description of *Aquabacter pokkalii* sp. nov

#### *Aquabacter pokkalii* (pok.ka'li.i. N.L. gen. n. *pokkalii* of pokkali (a variety of rice))

Cells are Gram-negative, non-motile, aerobic, and rod-shaped. Colonies are cream in color, smooth, and circular after 4 days on R2A-N agar medium at 30 °C. The strain is able to tolerate up to 3% NaCl and 37 °C. Able to grow in ZoBell marine agar and 100% seawater. Negative for hydrolysis of starch, pectin, CMC, and casein. Positive utilization for alanine, proline, glutamine, and methionine. Weak for tryptophan and putrescine. Negative for lysine, histidine, and arginine. Based on API 20NE strips, positive results were obtained for urease, hydrolysis of esculin, β-galactosidase, and assimilation for glucose, arabinose mannose, mannitol, maltose, potassium gluconate, malate, and trisodium citrate utilization; negative results were obtained for reduction of nitrates and nitrites, indole production, fermentation of glucose, arginine dihydrolase, hydrolysis of gelatin, N-acetyl-glucosamine, capric acid, adipic acid, phenylacetic acid and oxidase. According to Biolog GEN III MicroPlate, positive results were obtained for assimilation for L-galactonic acid lactone, bromo-succinic acid, β-hydroxy-D,L-butyric acid, L-glutamic acid, L-pyroglutamic acid, D-galacturonic acid, methylpyruvate, L-lactic acid, citric acid, D-malic acid, propionic acid, L-malic acid, sodium lactate and acetic acid, tetrazolium violet, sodium butyrate, and tetrazolium blue and growth at 1% NaCl, and pH 6, whereas negative results were obtained for assimilation for D-fucose, L-fucose, D-raffinose, α-D-glucose, α-D-lactose, D-mannose, D-maltose, D-melibiose, D-fructose, D-trehalose, D-galactose, D-cellobiose, gentiobiose, sucrose, D-turanose, L-rhamnose, stachyose, D-sorbitol, D-mannitol, D-arabitol, L-alanine, L-aspartic acid, L-arginine, D-aspartic acid, L-histidine, D-serine, L-serine, gelatin, glycyl-L-proline, glycerol, glucuronamide, α-ketoglutaric acid, pectin, tween 40, dextrin, D-lactic acid methylester, β-methyl-D-glucoside, myo-inositol, D-salicin, 3-methylglucose, N-acetyl-D glucosamine, D-glucose-6-PO_4_, N-acetyl-b-D-mannosamine, D-fructose-6-PO_4_, N-acetyl-D-galactosamine, inosine, D-saccharic acid, γ-aminobutyric acid, α-ketobutyric acid, α-hydroxybutyric acid, p-hydroxyphenylacetic acid, formic acid, D-gluconic acid, D-glucuronic acid, acetoacetic acid, mucic acid, quinic acid, N-acetyl neuraminic acid, guanidine HCl, lithium chloride, potassium tellurite, D-serine, and sodium bromate and growth at pH 5, 4% and 8% NaCl. Susceptible to antibiotics such as lincomycin, vancomycin, aztreonam, fusidic acid, and rifamycin SV. Resistant to troleandomycin, nalidixic acid, and minocycline. The major fatty acid is summed feature 8 (C18:1ω7c/C18:1ω6c), and the complete cellular fatty acid profile is given in Table S4. Other chemotaxonomy properties, including polar lipids and respiratory quinone, are given in the main text.

L1I39^T^ is positive for ACC deaminase production, in planta *nifH* gene expression and can promote pokkali rice growth by supplying fixed nitrogen under a nitrogen-deficient seawater condition. Its genome possesses multiple gene systems contributing to its plant growth promotion ability, host association, and brackish adaptations. The 16S rRNA metagenomic analysis identified L1I39^T^ as an integral part of the root-associated native microbiota of brackish-associated rice varieties but occurs with a lower abundance, conferring L1I39^T^ as a rare PGPR taxa.

The type strain L1I39^T^ (= LMG 28413^ T^ = MCC 4526^ T^) was isolated from the rhizosphere region of a salt-tolerant pokkali rice grown in Ernakulam, Kerala, India. The DNA G + C content is 66.9 mol% (derived from genome).

### Supplementary Information

**Additional file 1:** **Fig. S1**. Maximum Likelihood phylogenetic tree based on the concatenated alignments of six highly conserved housekeeping genes recA, gyrB, rpoB, dnaK, atpD, and gltA (overall 3885 amino acid positions) of L1I39T and related members of the family Xanthobacteraceae. Closed dark circles at each node represent a similar grouping obtained from the neighbor-joining and maximum parsimony algorithms. The significance of each branch is indicated by the bootstrap value (as a percentage) calculated for 1000 subsets. Escherichia coli ATCC 11775T was used as an outgroup. The bar indicates sequence divergence. **Fig. S2**. Total polar lipid profile of strain L1I39T obtained after two-dimensional thin layer chromatography method. The total lipid spots were detected by spraying with 5% ethanolic molybdatophosphoric acid. Abbreviations: L, Lipid; AL, Aminolipid; GL, Glycolipid; PL, Phospholipid; PC, Phosphatidylglycerine; PE, Phosphatidylethanolamine; PG, Phosphatidylglycerol. **Fig. S3**. The positive growth of L1I39T on ZoBell Marine agar plate after 10 days of incubation at 30°C. **Fig. S4**. (a) Imaging showing the growth curve of L1I39T in R2A broth prepared with different seawater concentrations. (b) Phenotypic growth of L1I39T in R2A broth containing different seawater concentrations (A) zero, (B) 20%, and (C) 60% after 24 hours of incubation at 30°C, as represented by (i) visual culture broth turbidity, (ii) recovery of L1I39T cells on respective R2A agar and (iii) a comparative bar graph. Statistical significance represented by *, *p*-value < 0.05; **, *p*-value < 0.01; ***, *p*-value < 0.005. **Fig. S5**. Plant growth-promoting effects of L1I39T under nitrogen-limiting zero seawater conditions, (a) pot images showing the growth of pokkali rice (L1I39T-treated and control plants) after 28 days post-inoculation, (b) uprooted images showing the no growth differences between the L1I39T-treated and control plants after 28 days post-inoculation, (c) bar graphs represent no significant growth differences observed in plant parameters such as (i) shoot length, (ii) root length, (iii) root fresh weight, and (vi) root dry weight, between (A) control and (B) L1I39T-treated pokkali plants after 28 days post-inoculation. **Fig. S6**. (a) biofilm formation of L1I39T on a microtiter plate; (i) and (ii) represent replicates, (b) epi-fluorescent image showing a firm pokkali rice root attachment of GFP-tagged L1I39T cells after 3 hrs of incubation under 20% seawater conditions; (i) and (ii) represent different regions of the primary root, scale bar 20µm, (c) The symbiosis polysaccharide (syp) gene cluster of L1I39T. **Fig. S7**. Image showing the visual growth turbidity of L1I39T under different concentrations of hydrogen peroxide after 24 hours at 30°C. ++, good growth; +, weak growth. **Fig. S8**. (a) Image showing the visual growth of L1I39T in minimal media containing glucose or DL-malic acid as the sole carbon sources, (b) Bar graph representing the comparative growth differences of L1I39T in minimal media containing respective carbon sources. Images and data taken after 60 h time point. **Fig. S9**. Identified vitamin biosynthesis gene clusters in L1I39T (a) cobalamine, (b) biotin, and (c) thiamine biosynthesis. **Fig. S10**. Maximum likelihood phylogenetic tree based on the concatenated NifHDK protein-coding sequences of L1I39T and related members of the family Xanthobacteraceae. The significance of each branch is indicated by the bootstrap value (as a percentage) calculated for 1000 subsets. The scale bar indicates nucleotide substitution per site. **Fig. S11**. The nitrogen fixation (nif) gene cluster comparison between L1I39T and related members of the family Xanthobacteraceae; A. cavernae Sn-9-2T, A. spiritensis DSM 9035T, A. caulinodans ORS 571T, and X. autotrophicus Py2. **Fig. S12**. Putative hydrogenase gene cluster identified in (a) L1I39T and (b) A. caulinodans ORS 571T. **Fig. S13**. Putative T4SS gene cluster identified in L1I39T genome. **Fig. S14**. (a) Genetic organization of T6SS-1 and T6SS-2 gene clusters of L1I39T (b) Cell-cell contact-dependent assay. (i) Image showing the recovery of E. coli cells (target strain) after co-culturing with (A) E. coli (self-negative control), (B) L1I39T (attacker), and (C) P. plantistimulans L1E11T (attacker-positive control) on a solid LB agar plate with tetracycline. (ii) Box plot representing the recovery of E. coli cells (target strain) after co-culturing with (A) E. coli (self-negative control), (B) L1I39T (attacker), and (C) P. plantistimulans L1E11T (attacker-positive control).

**Additional file 2**.

## Data Availability

The 16S rRNA gene sequence of L1I39T was deposited in NCBI under accession number KX533957. The draft genome sequence of L1I39T was deposited in NCBI with accession number CP072392.
