# Supplementary material for: Characterization of a novel root-associated diazotrophic rare PGPR taxa, Aquabacter pokkalii sp. nov., isolated from pokkali rice: new insights into the plant-associated lifestyle and brackish adaptation
Source: BMC Genomics. 2024 Apr 29;25:424. doi: 10.1186/s12864-024-10332-z (PMC11059613; doi:10.1186/s12864-024-10332-z)
Supplement: Supplementary file 1 — Additional file 1: Fig. S1. Maximum Likelihood phylogenetic tree based on the concatenated alignments of six highly conserved housekeeping genes recA, gyrB, rpoB, dnaK, atpD, and gltA (overall 3885 amino acid positions) of L1I39T and related members of the family Xanthobacteraceae. Closed dark circles at each node represent a similar grouping obtained from the neighbor-joining and maximum parsimony algorithms. The significance of each branch is indicated by the bootstrap value (as a percentage) calculated for 1000 subsets. Escherichia coli ATCC 11775T was used as an outgroup. The bar indicates sequence divergence. Fig. S2. Total polar lipid profile of strain L1I39T obtained after two-dimensional thin layer chromatography method. The total lipid spots were detected by spraying with 5% ethanolic molybdatophosphoric acid. Abbreviations: L, Lipid; AL, Aminolipid; GL, Glycolipid; PL, Phospholipid; PC, Phosphatidylglycerine; PE, Phosphatidylethanolamine; PG, Phosphatidylglycerol. Fig. S3. The positive growth of L1I39T on ZoBell Marine agar plate after 10 days of incubation at 30°C. Fig. S4. (a) Imaging showing the growth curve of L1I39T in R2A broth prepared with different seawater concentrations. (b) Phenotypic growth of L1I39T in R2A broth containing different seawater concentrations (A) zero, (B) 20%, and (C) 60% after 24 hours of incubation at 30°C, as represented by (i) visual culture broth turbidity, (ii) recovery of L1I39T cells on respective R2A agar and (iii) a comparative bar graph. Statistical significance represented by *, p-value < 0.05; **, p-value < 0.01; ***, p-value < 0.005. Fig. S5. Plant growth-promoting effects of L1I39T under nitrogen-limiting zero seawater conditions, (a) pot images showing the growth of pokkali rice (L1I39T-treated and control plants) after 28 days post-inoculation, (b) uprooted images showing the no growth differences between the L1I39T-treated and control plants after 28 days post-inoculation, (c) bar graphs represent no significa [file 12864_2024_10332_MOESM1_ESM.docx]

**
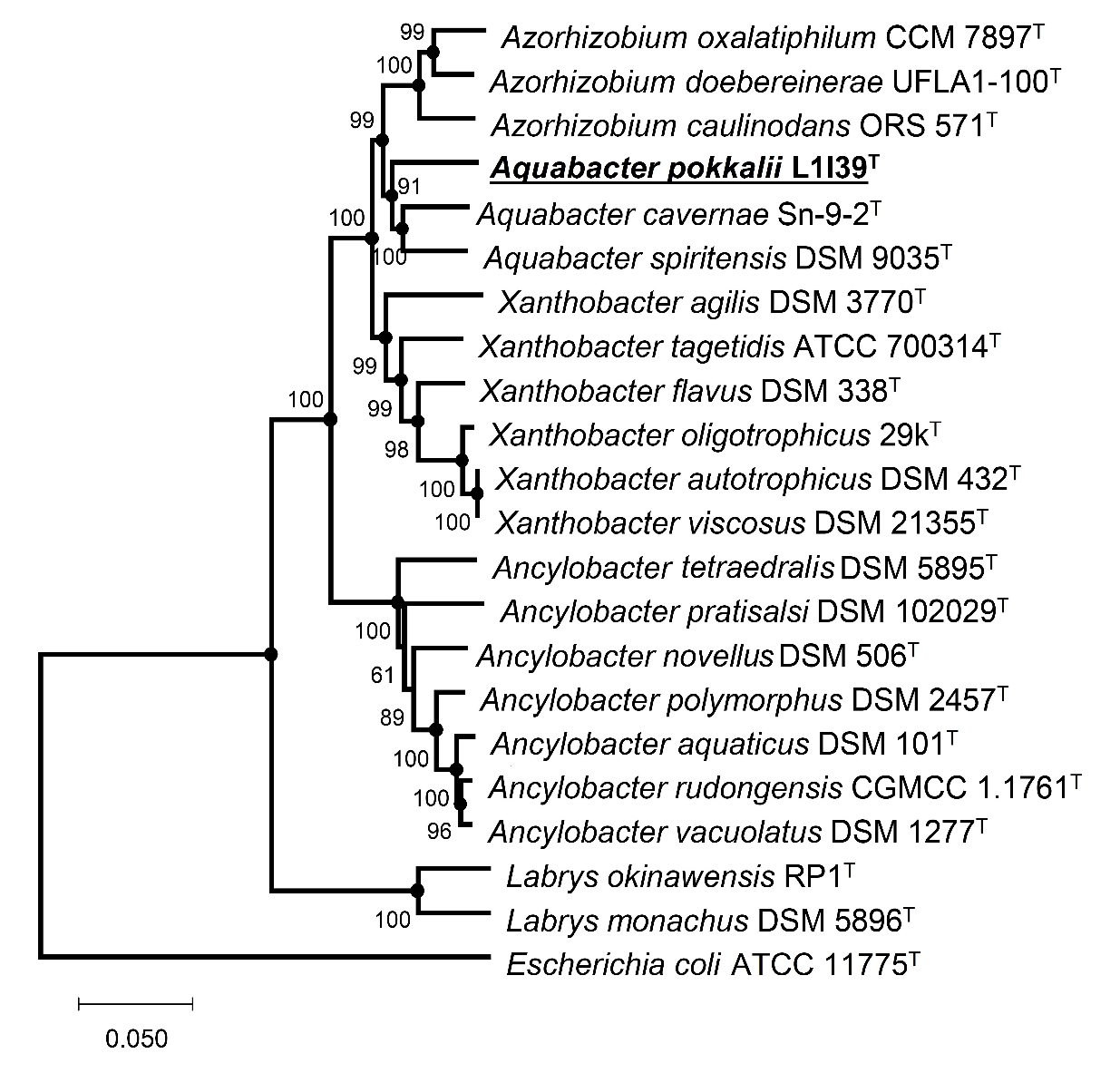
Fig. S1.** Maximum Likelihood phylogenetic tree based on the concatenated alignments of six highly conserved housekeeping genes *recA, gyrB, rpoB, dnaK, atpD,* and *gltA* (overall 3885 amino acid positions) of L1I39^T^ and related members of the family *Xanthobacteraceae*. Closed dark circles at each node represent a similar grouping obtained from the neighbor-joining and maximum parsimony algorithms. The significance of each branch is indicated by the bootstrap value (as a percentage) calculated for 1000 subsets. *Escherichia coli* ATCC 11775^T^ was used as an outgroup. The bar indicates sequence divergence.

**Fig. S2.** Total polar lipid profile of strain L1I39^T^ obtained after two-dimensional thin layer chromatography method. The total lipid spots were detected by spraying with 5% ethanolic molybdatophosphoric acid. Abbreviations: L, Lipid; AL, Aminolipid; GL, Glycolipid; PL, Phospholipid; PC, Phosphatidylglycerine; PE, Phosphatidylethanolamine; PG, Phosphatidylglycerol.

**
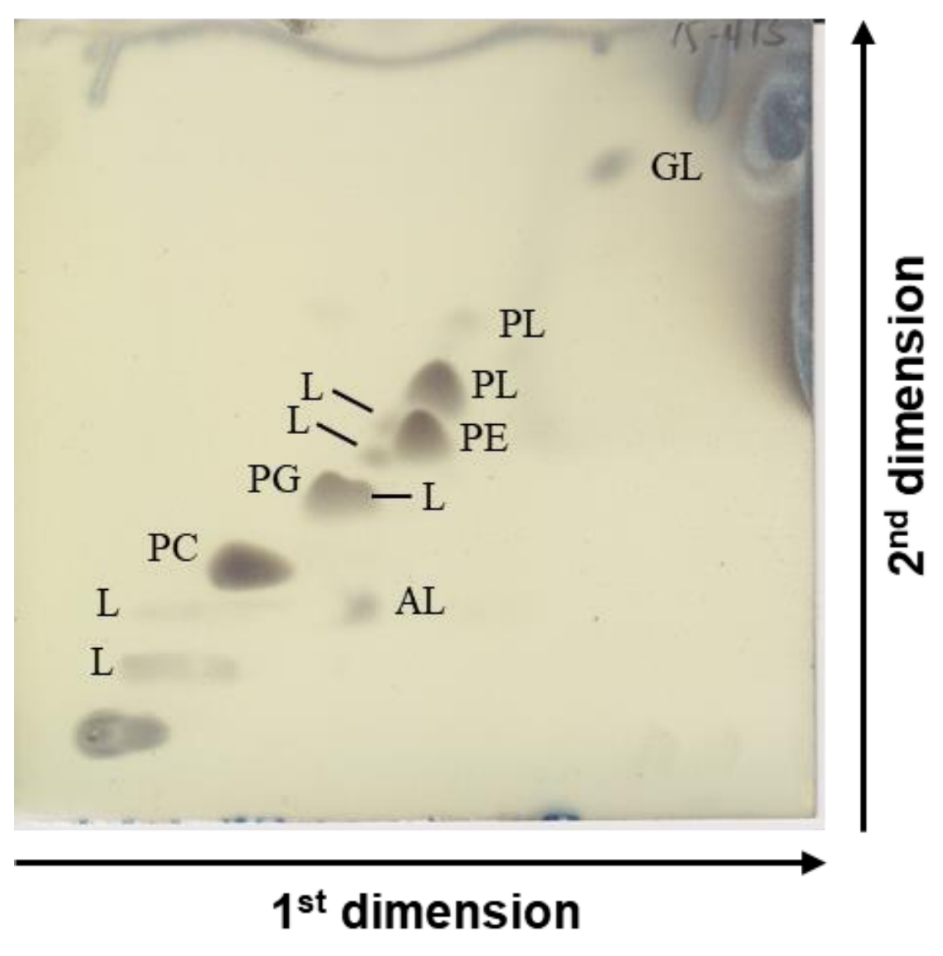
**

**Fig. S3.** The positive growth of L1I39^T^ on ZoBell Marine agar plate after 10 days of incubation at 30°C.


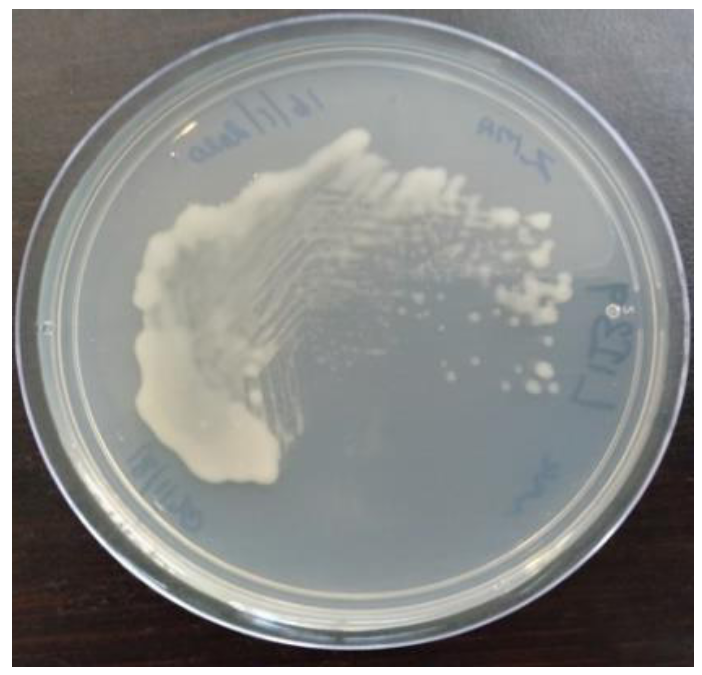


**Fig. S4. (a)** Imaging showing the growth curve of L1I39^T^ in R2A broth prepared with different seawater concentrations. **(b)** Phenotypic growth of L1I39^T^ in R2A broth containing different seawater concentrations (A) zero, (B) 20%, and (C) 60% after 24 hours of incubation at 30°C, as represented by **(i)** visual culture broth turbidity, **(ii)** recovery of L1I39^T^ cells on respective R2A agar and **(iii)** a comparative bar graph. Statistical significance represented by *, p-value < 0.05; **, p-value < 0.01; ***, p-value < 0.005.


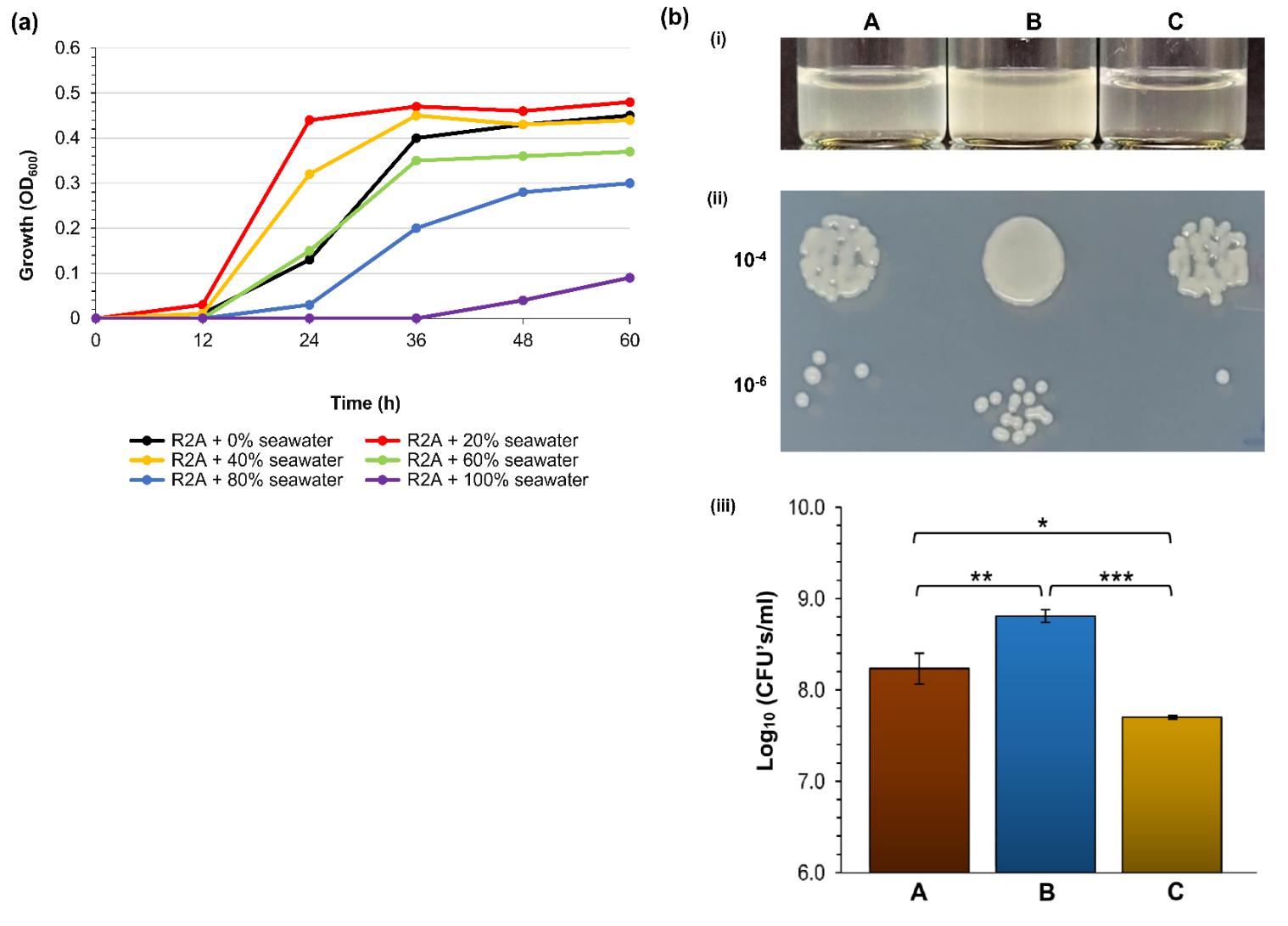


**Fig. S5.** Plant growth-promoting effects of L1I39^T^ under nitrogen-limiting zero seawater conditions, **(a)** pot images showing the growth of pokkali rice (L1I39^T^-treated and control plants) after 28 days post-inoculation, **(b)** uprooted images showing the no growth differences between the L1I39^T^-treated and control plants after 28 days post-inoculation**, (c)** bar graphs represent no significant growth differences observed in plant parameters such as **(i)** shoot length, **(ii)** root length, **(iii)** root fresh weight, and **(vi)** root dry weight, between (A) control and (B) L1I39^T^-treated pokkali plants after 28 days post-inoculation.

**
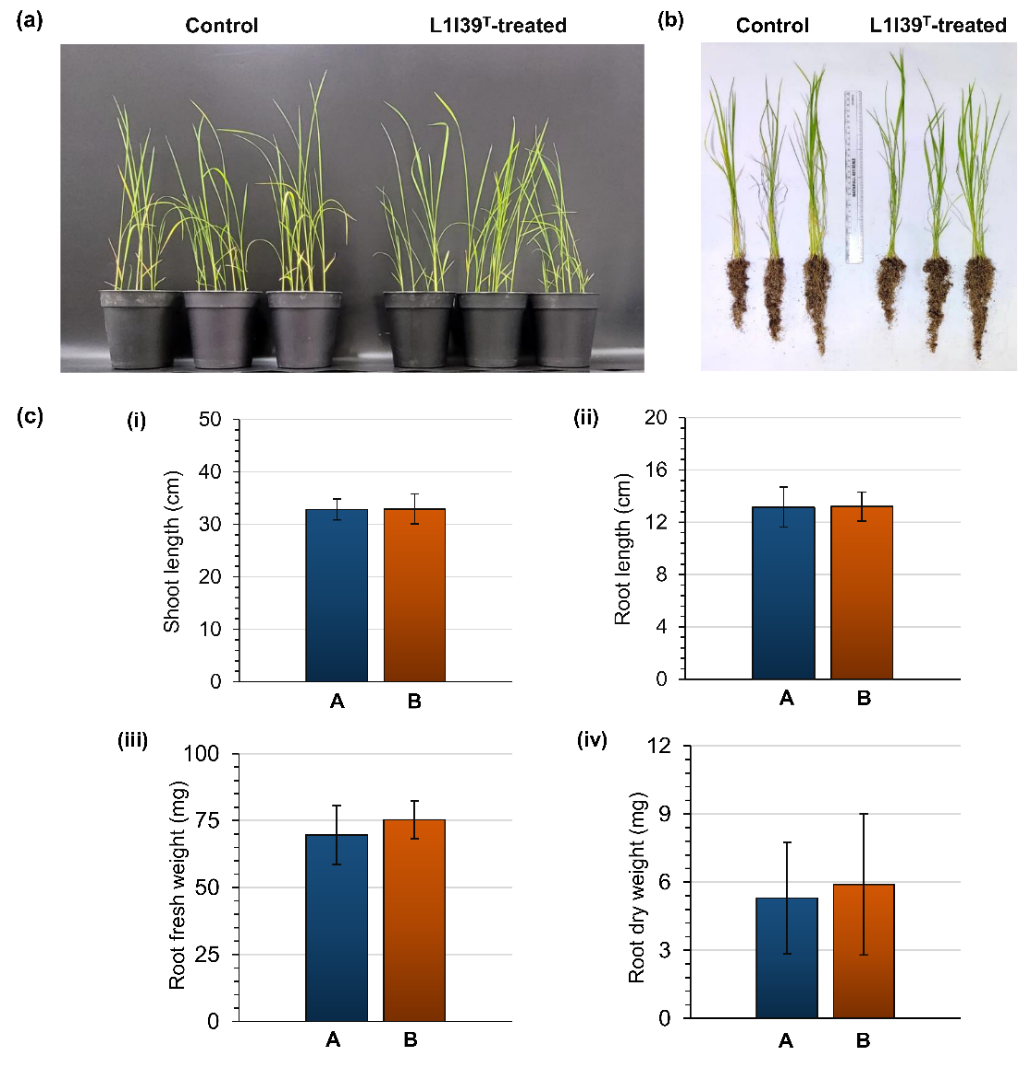
**

**Fig. S6. (a)** Biofilm formation of L1I39^T^ on a microtiter plate**; (i)** and **(ii)** represent replicates, **(b)** epi-fluorescent image showing a firm pokkali rice root attachment of GFP-tagged L1I39^T^ cells after 3 hrs of incubation under 20% seawater conditions; **(i)** and **(ii)** represent different regions of the primary root. Scale bars 20µm. **(c)** The symbiosis polysaccharide (*syp*) gene cluster of L1I39^T^,


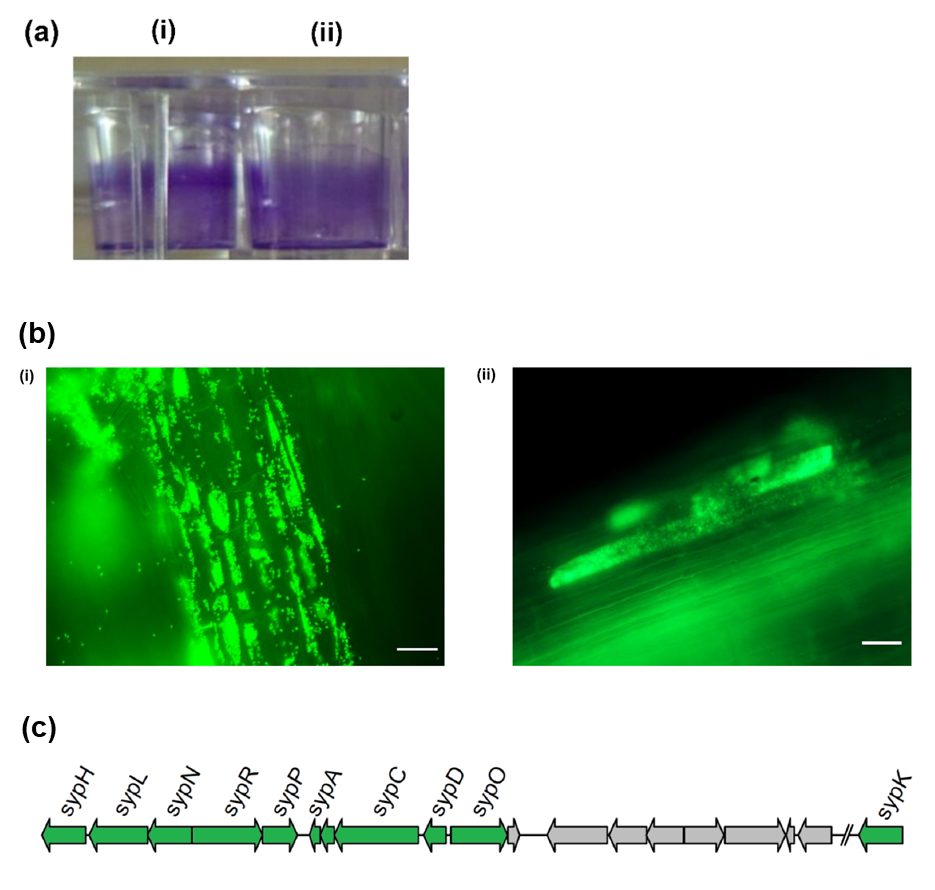


**Fig. S7.** Image showing the visual growth turbidity of L1I39^T^ under different concentrations of hydrogen peroxide after 24 hours at 30°C. ++, good growth; +, weak growth.

**
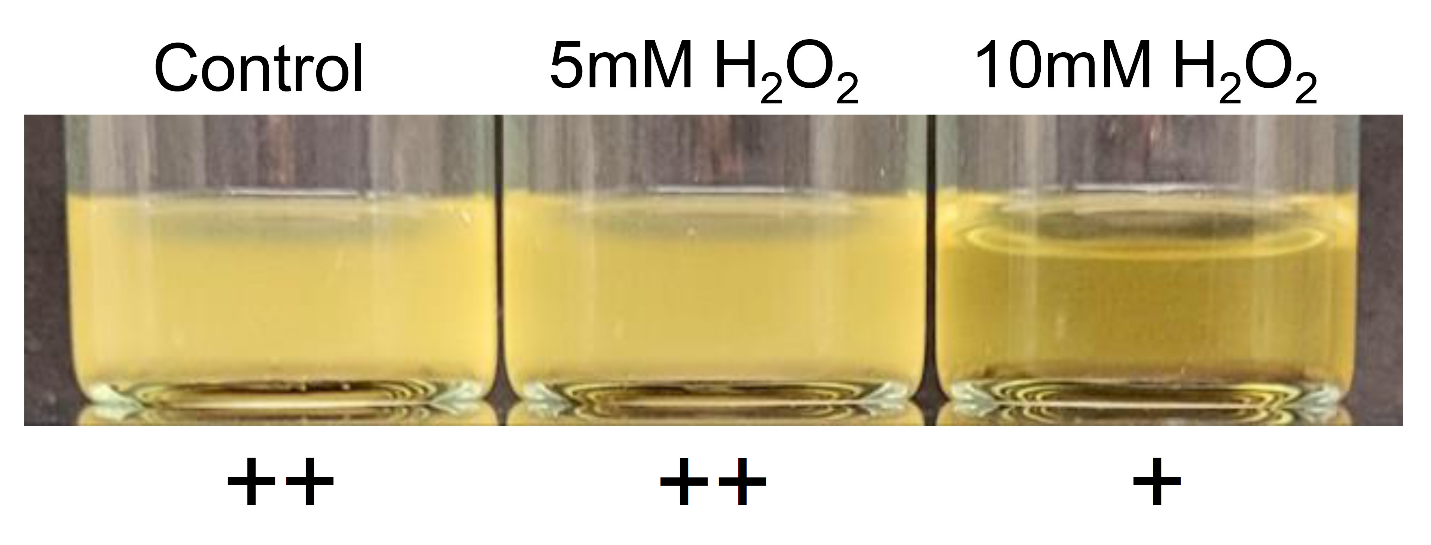
**

**Fig. S8. (a)** Image showing the visual growth of L1I39^T^ in minimal media containing glucose or DL-malic acid as the sole carbon sources, **(b)** Bar graph representing the comparative growth differences of L1I39^T^ in minimal media containing respective carbon sources. Images and data taken after 60 h time point.

**
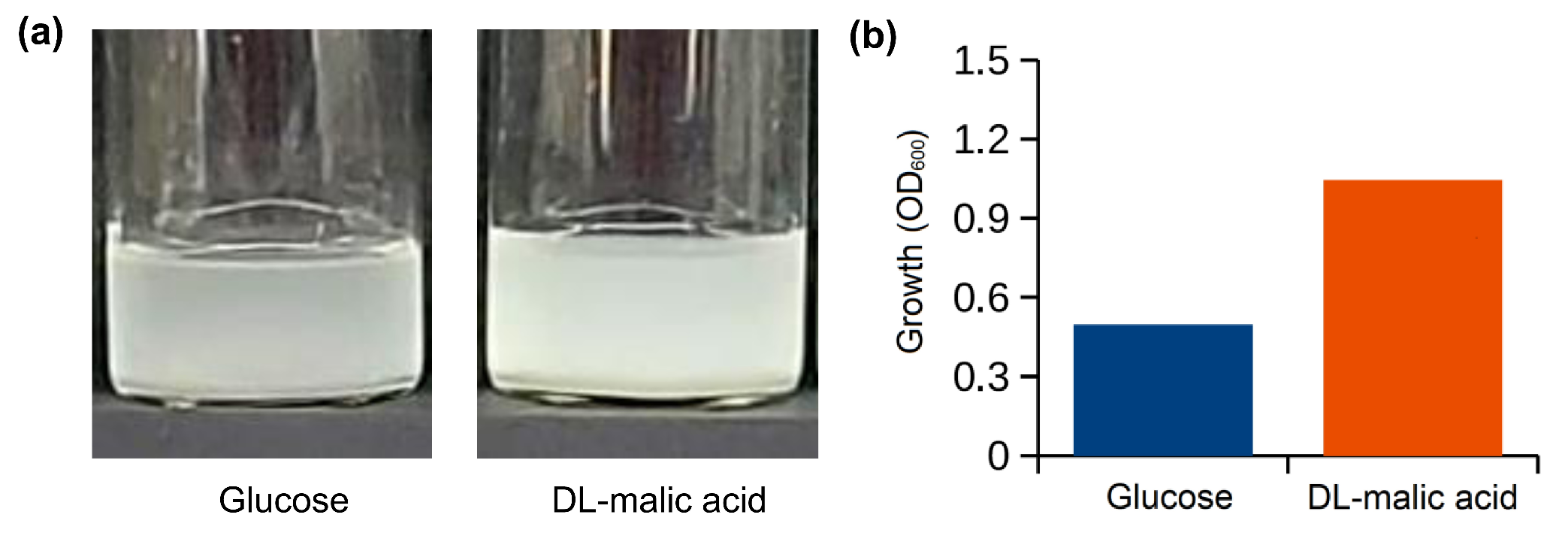
**

**Fig. S9**. Identified vitamin biosynthesis gene clusters in L1I39^T^ **(a)** cobalamine, **(b)** biotin, and **(c)** thiamine biosynthesis.


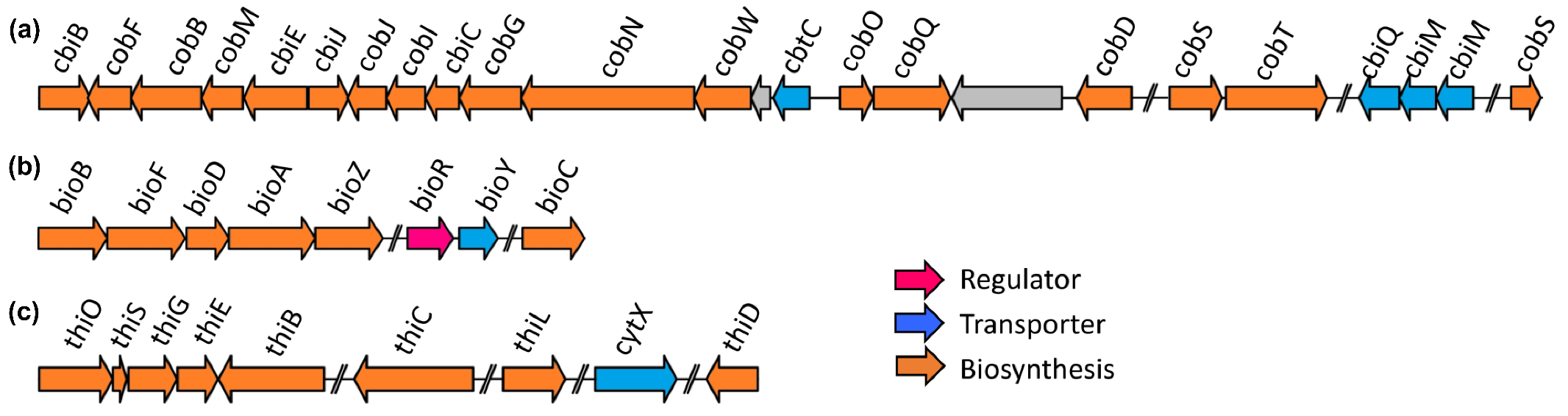


**Fig. S10.** Maximum likelihood phylogenetic tree based on the concatenated NifHDK protein-coding sequences of L1I39^T^ and related members of the family *Xanthobacteraceae*. The significance of each branch is indicated by the bootstrap value (as a percentage) calculated for 1000 subsets. The scale bar indicates nucleotide substitution per site.


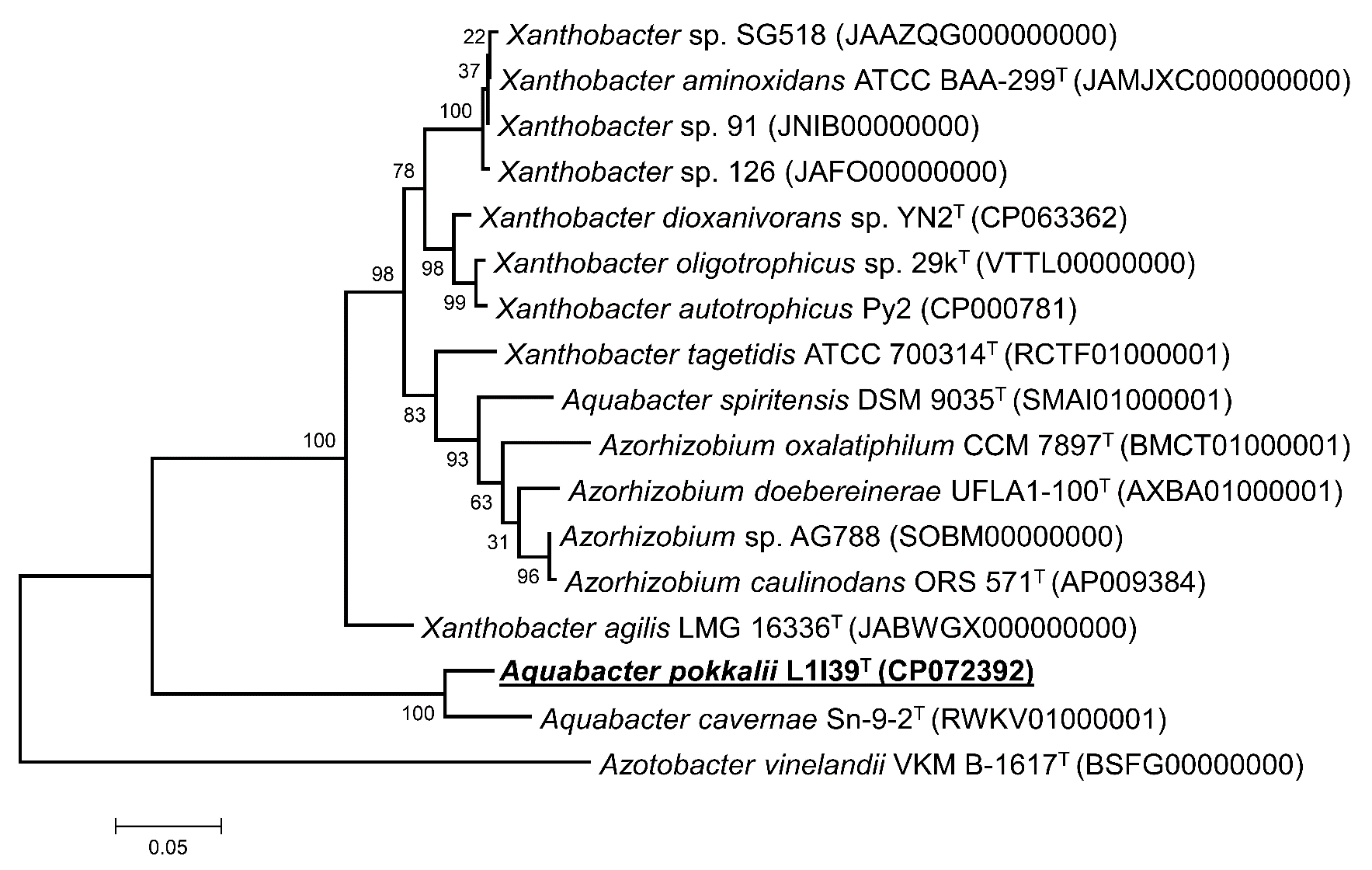


**Fig. S11.** The nitrogen fixation (*nif*) gene cluster comparison between L1I39^T^ and related members of the family *Xanthobacteraceae*; *A. cavernae* Sn-9-2^T^, *A. spiritensis* DSM 9035^T^, *A. caulinodans* ORS 571^T^, and *X. autotrophicus* Py2.

*
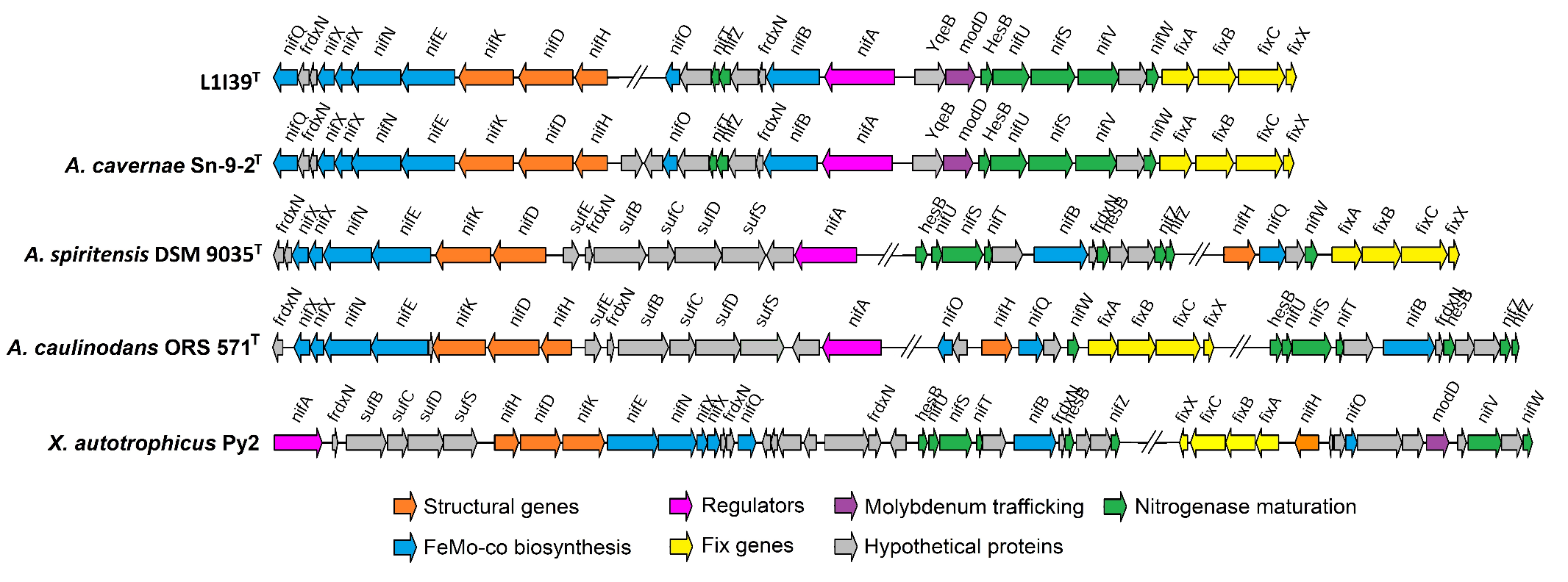
*

**Fig. S12.** Putative hydrogenase gene cluster identified in **(a)** L1I39^T^ and **(b)** *A. caulinodans* ORS 571^T^.


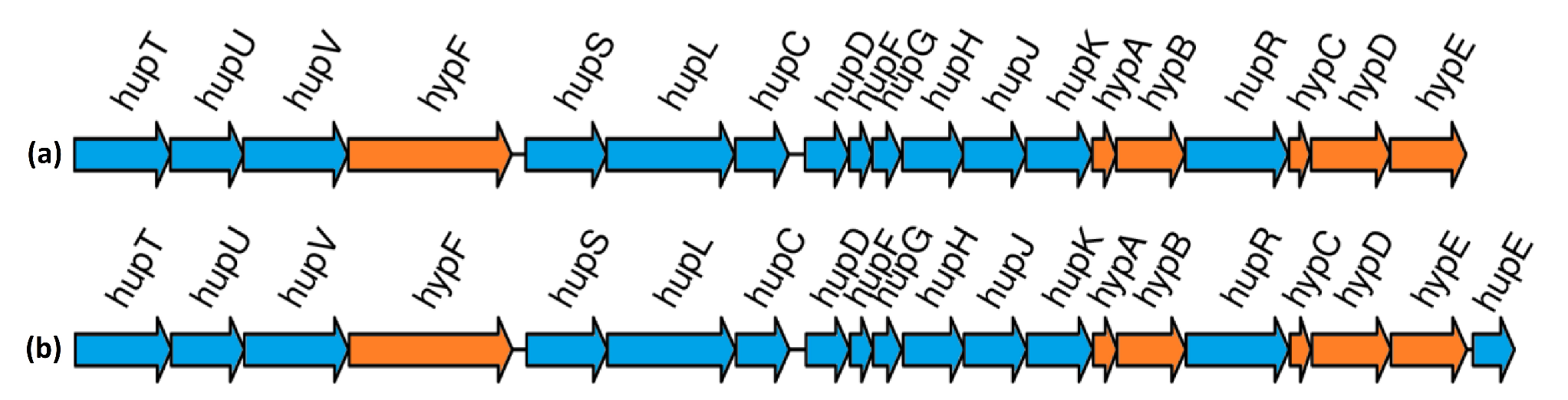


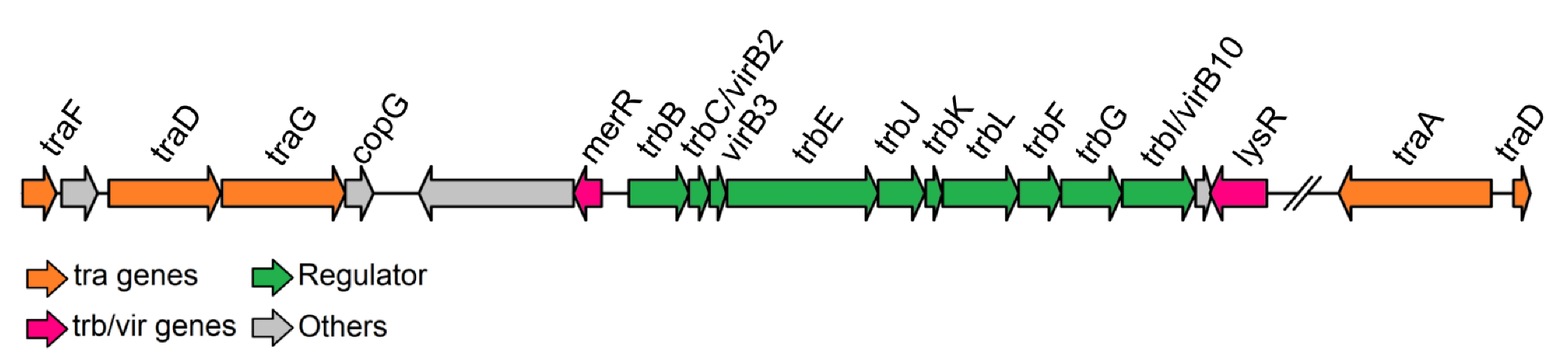
 **Fig. S13.** Putative T4SS gene cluster identified in L1I39^T^ genome.

**Fig. S14. (a)** Genetic organization of T6SS-1 and T6SS-2 gene clusters of L1I39^T^ **(b)** Cell-cell contact-dependent assay**. (i)** Image showing the recovery of *E. coli* cells (target strain) after co-culturing with (A) *E*. *coli* (self-negative control), (B) L1I39^T^ (attacker), and (C) *P. plantistimulans* L1E11^T^ (attacker-positive control) on a solid LB agar plate with tetracycline. **(ii)** Box plot representing the recovery of *E. coli* cells (target strain) after co-culturing with (A) *E*. *coli* (self-negative control), (B) L1I39^T^ (attacker), and (C) *P. plantistimulans* L1E11^T^ (attacker-positive control).

**
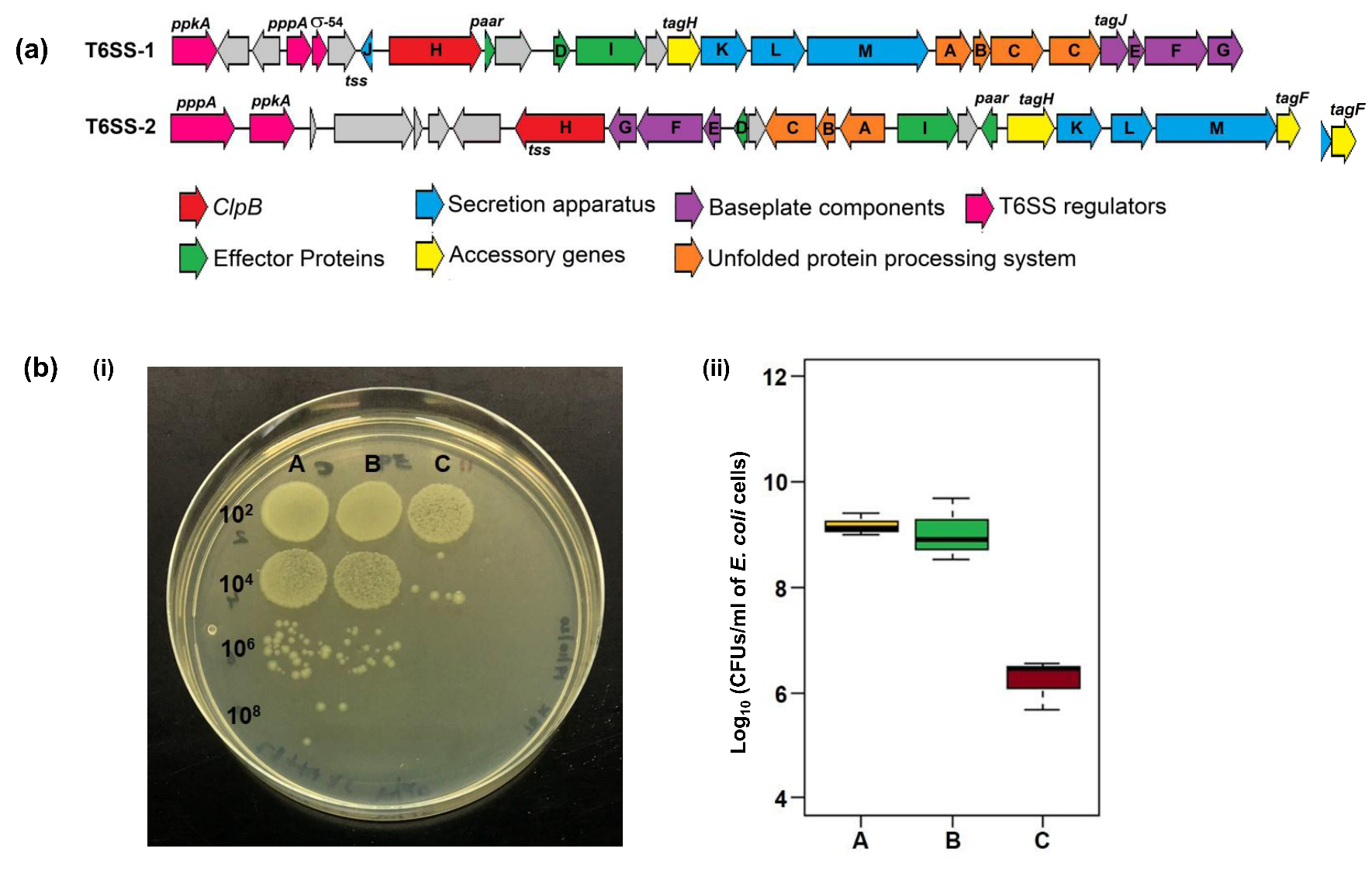
**
