# Supplementary material for: Characterization of a novel root-associated diazotrophic rare PGPR taxa, Aquabacter pokkalii sp. nov., isolated from pokkali rice: new insights into the plant-associated lifestyle and brackish adaptation
Source: BMC Genomics. 2024 Apr 29;25:424. doi: 10.1186/s12864-024-10332-z (PMC11059613; doi:10.1186/s12864-024-10332-z)
Supplement: Supplementary file 2 — Additional file 2. [file 12864_2024_10332_MOESM2_ESM.docx]

**Table S1.** The genome accession numbers of different strains used in multilocus sequence analysis and genome-based phylogenetic tree constructions.

| **Sl no.** | **Strain name** | **Genome accession number** |
| --- | --- | --- |
| 1 | *Ancylobacter aquaticus* DSM 101^T^ | SMFY01000001 |
| 2 | *Ancylobacter mangrovi* GSK1Z-4-2^T^ | JANTHY000000000 |
| 3 | *Ancylobacter novellus* DSM 506^T^ | CP002026 |
| 4 | *Ancylobacter polymorphus* DSM 2457^T^ | JAUSUI000000000 |
| 5 | *Ancylobacter pratisalsi* DSM 102029^T^ | CP048630 |
| 6 | *Ancylobacter rudongensis* CGMCC 1.1761^T^ | FMTP01000001 |
| 7 | *Ancylobacter tetraedralis* DSM 5895^T^ | JACICD010000001 |
| 8 | *Ancylobacter vacuolatus* DSM 1277^T^ | JAUSUH000000000 |
| 9 | *Aquabacter cavernae* Sn-9-2^T^ | RWKV01000001 |
| 10 | *Aquabacter spiritensis* DSM 9035^T^ | SMAI01000001 |
| 11 | *Azorhizobium caulinodans* ORS 571^T^ | AP009384 |
| 12 | *Azorhizobium doebereinerae* UFLA1-100^T^ | AXBA01000001 |
| 13 | *Azorhizobium oxalatiphilum* CCM 7897^T^ | BMCT01000001 |
| 14 | *Escherichia coli* ATCC 11775^T^ | CP033091 |
| 15 | *Labrys monachus* DSM 5896^T^ | JAUSVK000000000 |
| 16 | *Labrys okinawensis* RP1^T^ | PUEJ01000001 |
| 17 | *Xanthobacter agilis* DSM 3770^T^ | JABWGX000000000 |
| 18 | *Xanthobacter autotrophicus* DSM 432^T^ | VAUP01000001 |
| 19 | *Xanthobacter flavus* DSM 338^T^ | BSDO00000000 |
| 20 | *Xanthobacter oligotrophicus* 29k^T^ | VTTL00000000 |
| 21 | *Xanthobacter tagetidis* ATCC 700314^T^ | RCTF01000001 |
| 22 | *Xanthobacter* *viscosus* DSM 21355^T^ | 1281864 |

**Table S2.** Multilocus sequence analysis of strain L1I39^T^ and related phylogenetic members of the family *Xanthobacteraceae* based on the individual and concatenated sequences of six housekeeping genes (*atpD, recA, gyrB, dnaK, gltA, rpoB*).

| **Strain name** | ***atpD*** | ***recA*** | ***gyrB*** | ***dnaK*** | ***gltA*** | ***rpoB*** | ***Concat-enated*** |
| --- | --- | --- | --- | --- | --- | --- | --- |
| *Aquabacter cavernae* Sn-9-2^T^ | 92.49 | 86.43 | 85.76 | 89.71 | 90.09 | 91.52 | 89.75 |
| *Aquabacter spiritensis* DSM 9035^T^ | 90.26 | 85.34 | 84.57 | 88.44 | 84.24 | 90.02 | 87.94 |
| *Azorhizobium caulinodans* ORS 571^T^ | 91.58 | 88.98 | 84.56 | 89.85 | 87.67 | 89.87 | 88.88 |
| *Azorhizobium doebereinerae* UFLA1-100^T^ | 91.58 | 88.36 | 85.23 | 90.17 | 88.46 | 89.82 | 89.02 |
| *Azorhizobium oxalatiphilum* CCM 7897^T^ | 91.52 | 87.40 | 83.67 | 90.08 | 87.62 | 88.73 | 88.39 |
| *Xanthobacter tagetidis* ATCC 700314^T^ | 90.68 | 87.12 | 83.88 | 89.17 | 87.56 | 90.31 | 88.60 |
| *Xanthobacter flavus* DSM 338^T^ | 91.30 | 86.22 | 83.20 | 89.78 | 87.05 | 89.95 | 88.26 |
| *Xanthobacter agilis* DSM 3770^T^ | 87.02 | 87.48 | 82.43 | 90.33 | 87.76 | 87.52 | 87.17 |
| *Xanthobacter oligotrophicus* 29k^T^ | 90.47 | 85.98 | 82.49 | 89.18 | 87.53 | 89.97 | 88.10 |
| *Xanthobacter autotrophicus* DSM 432^T^ | 89.81 | 86.08 | 83.08 | 88.83 | 86.53 | 89.76 | 87.94 |
| *Xanthobacter viscosus* DSM 21355^T^ | 89.81 | 86.08 | 83.08 | 88.83 | 86.53 | 89.76 | 87.94 |
| *Ancylobacter novellus* DSM 506^T^ | 89.97 | 85.19 | 82.81 | 86.72 | 84.08 | 86.63 | 86.00 |
| *Ancylobacter tetraedralis* DSM 5895^T^ | 89.78 | 84.50 | 80.75 | 87.15 | 84.73 | 85.87 | 85.42 |
| *Ancylobacter polymorphus* DSM 2457^T^ | 88.93 | 84.20 | 81.07 | 87.00 | 83.58 | 85.41 | 85.10 |
| *Ancylobacter rudongensis* CGMCC 1.1761^T^ | 88.44 | 84.48 | 79.18 | 86.37 | 84.74 | 85.66 | 84.80 |
| *Ancylobacter vacuolatus* DSM 1277^T^ | 88.86 | 83.70 | 79.08 | 85.95 | 84.17 | 85.88 | 84.67 |
| *Ancylobacter aquaticus* DSM 101^T^ | 88.03 | 83.80 | 83.24 | 85.92 | 85.49 | 86.12 | 84.92 |
| *Ancylobacter mangrovi* GSK1Z-4-2^T^ | 89.75 | 83.98 | 81.70 | 86.86 | 84.46 | 86.59 | 85.20 |
| *Ancylobacter pratisalsi* DSM 102029^T^ | 87.68 | 80.72 | 81.79 | 86.72 | 83.05 | 85.08 | 84.52 |
| *Labrys okinawensis* RP1^T^ | 82.82 | 81.52 | 81.75 | 83.92 | 82.90 | 82.58 | 81.32 |
| *Labrys monachus* DSM 5896^T^ | 84.12 | 81.44 | 77.84 | 84.44 | 83.13 | 81.98 | 80.83 |

**Table S3.** Genome-relatedness similarity (ANI, AAI, and dDDH) calculations between L1I39^T^ and related phylogenetic members of the family *Xanthobacteraceae*.

| Strains | Reference genome L1I39^T^ | | |
| --- | --- | --- | --- |
|  | ANIb (%) | AAI (%) | dDDH (%) |
| *Aquabacter cavernae* sp. Sn-9-2^T^ | 76.79 | 73.19 | 22.5 |
| *Aquabacter spiritensis* DSM 9035^T^ | 75.25 | 72.60 | 22.1 |
| *Azorhizobium caulinodans* ORS 571^T^ | 75.40 | 70.67 | 22.3 |
| *Xanthobacter autotrophicus* DSM 432^T^ | 75.43 | 69.43 | 22.2 |
| *Xanthobacter oligotrophicus* 29k^T^ | 75.75 | 65.29 | 22.7 |
| *Azorhizobium doebereinerae* UFLA1-100^T^ | 75.90 | 70.63 | 22.2 |
| *Azorhizobium oxalatiphilum* CCM 7897^T^ | 74.68 | 68.79 | 21.8 |
| *Xanthobacter flavus* DSM 338^T^ | 75.48 | 69.92 | 22.5 |
| *Xanthobacter viscosus* DSM 21355^T^ | 75.46 | 69.53 | 22.2 |
| *Xanthobacter autotrophicus* DSM 432^T^ | 75.43 | 69.35 | 22.2 |
| *Xanthobacter tagetidis* ATCC 700314^T^ | 75.08 | 68.55 | 21.9 |
| *Xanthobacter agilis* DSM 3770^T^ | 74.43 | 68.20 | 21.6 |
| *Ancylobacter polymorphus* DSM 2457^T^ | 72.26 | 61.89 | 20.6 |
| *Ancylobacter novellus* DSM 506^T^ | 72.24 | 61.05 | 21.0 |
| *Ancylobacter tetraedralis* DSM 5895^T^ | 72.14 | 61.44 | 21.6 |
| *Ancylobacter rudongensis* CGMCC 1.1761^T^ | 71.95 | 61.60 | 20.1 |
| *Ancylobacter vacuolatus* DSM 1277^T^ | 71.73 | 61.36 | 20.4 |
| *Ancylobacter aquaticus* DSM 101^T^ | 71.46 | 60.82 | 20.2 |
| *Ancylobacter mangrovi* GSK1Z-4-2^T^ | 71.69 | 60.58 | 20.7 |
| *Ancylobacter pratisalsi* DSM 102029^T^ | 71.41 | 61.26 | 20.9 |
| *Labrys okinawensis* RP1^T^ | 69.07 | 54.37 | 19.2 |
| *Labrys monachus* DSM 5896^T^ | 69.02 | 54.95 | 19.7 |

**Table S4.** Whole-cell fatty acid composition of L1I39^T^ and its nearest phylogenetic neighbors. 1, L1I39^T^; 2, *A. cavernae* Sn-9-2^T^; 3, *A. spiritensis* LMG 8611^T^; 4, *A. caulinodans* DSM 5975^T^; 5, *X. autotrophicus* DSM 432^T^. Data for L1I39^T^ and *A. spiritensis* LMG 8611^T^ is from this study, data for *A. cavernae* Sn-9-2^T^ were taken from Duo et al., 2019 (Duo et al., 2019) and data for *A. caulinodans* DSM 5975^T^ and *X.* *autotrophicus* DSM 432^T^ were taken from Lang et al., 2013 (Lang et al., 2013). Only fatty acid percentages amounting to 0.8% or higher are shown—symbols: tr, (<0.5%); -, not detected.

| Fatty acid (%) | 1 | 2 | 3 | 4 | 5 |
| --- | --- | --- | --- | --- | --- |
| Saturated fatty acids | | | | | |
| C_16:0_ | 0.95 | 5.8 | 5.71 | 2.3 | 2.7 |
| C_16:0_ 3-OH | 0.64 | 1.3 | - | tr | 1.1 |
| C_18:0_ | 3.99 | 4.00 | 4.07 | 2.6 | 1.6 |
| C_19:0_ cyclo ω8c | - | - | 4.18 | 4.1 | tr |
| Unsaturated fatty acids | | | | | |
| C_18:1_ ω7c 11-methyl | 3.19 | 3.70 | 4.05 | 5.6 | tr |
| C_20:1_ ω7c | - | - |  | tr | 2.3 |
| Summed feature 2* | - | - |  | 1.7 | tr |
| Summed feature 3* | - | tr |  | 1.1 | 5.7 |
| Summed feature 8* | 88.79 | 83.9 | 81.99 | 80.00 | 86.3 |

Summed features represent groups of two fatty acids that could not be separated by GLC and the MIDI system. Summed feature 2 contained C_14:0_ 3-OH and/or iso-C_16:1_, feature 3 contained C_16:1_ω6c and/or C_16:1_ω7c, and feature 8 contained C_18:1_ω6c and/or C_18:1_ω7c

**Table S5.** General genome features of L1I39^T^ and its nearest phylogenetic neighbors. 1, L1I39^T^; 2, *A. cavernae* Sn-9-2^T^; 3, *A. spiritensis* LMG 8611^T^.

| **Genome features** | **1** | **2** | **3** |
| --- | --- | --- | --- |
| Genome size (bp) | 5388224 | 4516483 | 5162879 |
| GC content (%) | 66.9 | 67.5 | 67.6 |
| Total genes | 4942 | 4040 | 4784 |
| Protein coding genes | 4890 | 3987 | 4735 |
| rRNA genes | 3 | 6 | 3 |
| tRNA genes | 49 | 47 | 46 |
| Hypothetical CDS | 691 | 659 | 670 |
| Transposases | 3 | 3 | 29 |
| Regulatory genes | 309 | 254 | 290 |
| Sigma subunits | 20 | 13 | 17 |
| Transport genes | 759 | 581 | 692 |

**Table S6.** SEED subsystem-based functional gene categories comparison between L1I39^T^ and its closely related strains from genus *Aquabacter*. The number of genes assigned in each subsystem feature is annotated using the RAST server. Strains: 1, L1I39^T^; 2, *A. cavernae* Sn-9-2^T^; and 3, *A. spiritensis* DSM 9035^T^

| **Subsystem features** | **1** | **2** | **3** |
| --- | --- | --- | --- |
| Cofactors, Vitamins, Prosthetic Groups, Pigments | 301 | 268 | 339 |
| Cell Wall and Capsule | 104 | 92 | 106 |
| Virulence, Disease and Defense | 86 | 85 | 108 |
| Potassium metabolism | 23 | 22 | 21 |
| Miscellaneous | 64 | 56 | 70 |
| Phages, Prophages, Transposable elements, Plasmid | 29 | 23 | 26 |
| Membrane Transport | 336 | 234 | 345 |
| Iron acquisition and metabolism | 26 | 15 | 7 |
| RNA Metabolism | 145 | 142 | 146 |
| Nucleosides and Nucleotides | 120 | 89 | 103 |
| Protein Metabolism | 255 | 252 | 264 |
| Cell Division and Cell Cycle | 26 | 23 | 27 |
| Motility and Chemotaxis | 79 | 75 | 79 |
| Regulation and Cell signaling | 81 | 69 | 80 |
| Secondary Metabolism | 5 | 5 | 6 |
| DNA Metabolism | 97 | 67 | 91 |
| Fatty Acids, Lipids, and Isoprenoids | 246 | 166 | 195 |
| Nitrogen Metabolism | 83 | 62 | 68 |
| Dormancy and Sporulation | 2 | 2 | 2 |
| Respiration | 189 | 134 | 181 |
| Stress Response | 144 | 119 | 139 |
| Metabolism of Aromatic Compounds | 94 | 46 | 104 |
| Amino Acids and Derivatives | 631 | 472 | 470 |
| Sulfur Metabolism | 67 | 55 | 82 |
| Phosphorus Metabolism | 59 | 58 | 66 |
| Carbohydrates | 506 | 355 | 424 |

**Table S7.** Genes coding for flagellar motility, chemotaxis, and methyl-accepting chemotaxis proteins (MCP) identified in the genome of the L1I39^T^

| **Accession no.** | **Gene name** | **Gene** |
| --- | --- | --- |
| **Flagellar-based motility** | | |
| WP_247657912.1 | DUF1217 domain protein/ Flagellar basal-body rod protein | *flgF* |
| WP_209103397.1 | rod-binding protein/ Flagellar protein | *flgJ* |
| WP_209103398.1 | hypothetical protein |  |
| WP_209103399.1 | flagellar biosynthesis protein | *fliR* |
| WP_209103400.1 | flagellar biosynthesis protein | *flhA* |
| WP_209103401.1 | flagellar biosynthetic protein | *fliQ* |
| WP_209103402.1 | flagellar hook assembly protein | *flgD* |
| WP_209105208.1 | flagellar biosynthesis repressor | *flgD* |
| WP_209103403.1 | flagellar biosynthesis regulator | *flaF* |
| WP_209103404.1 | flagellar hook-associated family protein | *flgL* |
| WP_209103405.1 | flagellar hook-associated protein | *flgK* |
| WP_209103406.1 | flagellar hook protein | *flgE* |
| WP_209103407.1 | DUF4214 domain-containing protein |  |
| WP_209103408.1 | flagellar protein export ATPase | *fliI* |
| WP_209103409.1 | flagellar basal-body rod protein | *flgF* |
| WP_209103410.1 | flagellar motor stator protein | *motA* |
| WP_209103411.1 | FliM/FliN family flagellar motor switch protein | *fliM* |
| WP_209103412.1 | hypothetical protein |  |
| WP_209103413.1 | flagellar motor switch protein | *fliN* |
| WP_209105209.1 | flagellar motor switch protein | *fliG* |
| WP_209103414.1 | flagellar biosynthesis protein | *flhB* |
| WP_209103415.1 | hypothetical protein |  |
| WP_209103416.1 | flagellar basal body rod protein | *flgB* |
| WP_209105210.1 | flagellar basal body rod protein | *flgC* |
| WP_209103417.1 | flagellar hook-basal body complex protein | *fliE* |
| WP_209103418.1 | flagellar basal-body rod protein | *flgG* |
| WP_209103419.1 | flagellar basal body P-ring formation chaperone | *flgA* |
| WP_209103420.1 | flagellar basal body P-ring protein | *flgI* |
| WP_209105211.1 | MotE family protein | *motE* |
| WP_209103421.1 | flagellar basal body L-ring protein | *flgH* |
| WP_209103422.1 | flagellar basal body-associated FliL family protein | *fliL* |
| WP_209103423.1 | flagellar type III secretion system pore protein | *fliP* |
| WP_209103424.1 | flagellin | *flaA* |
| WP_209103425.1 | flagellar M-ring protein | *fliF* |
| WP_209103426.1 | hypothetical protein |  |
| WP_247657965.1 | MotB family protein | *motB* |
| WP_209103427.1 | hypothetical protein/ Chemotaxis protein | *motC* |
| WP_209104055.1 | peptidoglycan -binding protein | *motB* |
| WP_209104056.1 | flagellar motor protein | *motA* |
| WP_209104539.1 | flagellar export protein | *fliJ* |
| **Chemotaxis** | | |
| WP_209103390.1 | protein-glutamate O-methyltransferase | *cheR* |
| WP_209105207.1 | chemotaxis response regulator protein-glutamate methylesterase | *cheB* |
| WP_209103391.1 | response regulator | *cheY* |
| WP_209103392.1 | chemotaxis protein | *cheW* |
| WP_209103393.1 | chemotaxis protein | *cheA* |
| WP_209103394.1 | methyl-accepting chemotaxis protein | MCP |
| WP_209103961.1 | response regulator | *cheY* |
| WP_209104601.1 | response regulator | *cheY* |
| WP_209100294.1 | response regulator | *cheV* |
| WP_209099605.1 | chemotaxis protein | *cheB* |
| WP_209099607.1 | protein-glutamate O-methyltransferase | *cheR* |
| WP_209103457.1 | HAMP domain-containing histidine kinase | *cheY* |
| WP_209102343.1 | PAS domain-containing sensor histidine kinase | *cheR* |
| **Methyl accepting chemotaxis proteins (MCP)** | | |
| WP_209103232.1 | methyl-accepting chemotaxis protein | MCP |
| WP_209103959.1 | methyl-accepting chemotaxis protein | MCP |
| WP_209104094.1 | methyl-accepting chemotaxis protein | MCP |
| WP_209104106.1 | PAS domain-containing methyl-accepting chemotaxis protein | MCP |
| WP_209104107.1 | PAS domain-containing methyl-accepting chemotaxis protein | MCP |
| WP_247658215.1 | HAMP domain-containing methyl-accepting chemotaxis protein | MCP |
| WP_209104442.1 | TIGR02302 family protein | MCP |
| WP_209104780.1 | methyl-accepting chemotaxis protein | MCP |
| WP_209104966.1 | methyl-accepting chemotaxis protein | MCP |
| WP_209101563.1 | hypothetical protein | MCP |
| WP_209101748.1 | CHASE3 domain-containing protein | MCP |

**Table S8.** Genes coding for ROS detoxification found in the L1I39^T^ genome

| **Accession no.** | **Gene name** |
| --- | --- |
| WP_209098707.1 | Glutathione S-transferase |
| WP_209104371.1 | Glutathione S-transferase |
| WP_209098668.1 | Glutathione S-transferase |
| WP_209098817.1 | Glutathione S-transferase |
| WP_209099173.1 | Glutathione S-transferase |
| WP_209099748.1 | Glutathione S-transferase |
| WP_209100185.1 | Glutathione S-transferase |
| WP_209100339.1 | Glutathione S-transferase |
| WP_209100968.1 | Glutathione S-transferase |
| WP_209102718.1 | Glutathione S-transferase |
| WP_209102731.1 | Glutathione S-transferase |
| WP_209103122.1 | Glutathione S-transferase |
| WP_209100967.1 | Glutathione S-transferase |
| WP_209101396.1 | OsmC family protein |
| WP_209098234.1 | Grx4 family monothiol glutaredoxin |
| WP_209102894.1 | glutaredoxin 3 |
| WP_209099074.1 | catalase |
| WP_209104859.1 | catalase/peroxidase |
| WP_209099076.1 | hydrogen peroxide-inducible genes activator |
| WP_209102794.1 | hydrogen peroxide-inducible genes activator |
| WP_209099862.1 | superoxide dismutase |
| WP_209098604.1 | organic hydroperoxide resistance protein |
| WP_247658531.1 | thioredoxin |
| WP_209104618.1 | thioredoxin-disulfide reductase |
| WP_209100803.1 | peroxiredoxin (Alkyl hydroperoxide reductase subunit C-like protein) |
| WP_209102406.1 | peroxiredoxin (Thiol peroxidase, Bcp-type) |
| WP_209102641.1 | peroxiredoxin (Alkyl hydroperoxide reductase subunit C-like protein) |
| WP_209104018.1 | peroxiredoxin |
| WP_209103105.1 | glutathione transferase |
| WP_209102652.1 | glutathione-disulfide reductase |
| WP_209102879.1 | Arsenate reductase (glutaredoxin) |

**Table S9.** Genes coding for energy metabolism identified in the L1I39^T^ genome

| **Accession no.** | **Gene name** | **Gene** |
| --- | --- | --- |
| **NADH/ubiquinone oxidoreductase (respiratory complex I)** | |  |
| WP_209102593.1 | NADH-quinone oxidoreductase subunit | *nuoN* |
| WP_209102595.1 | NADH-quinone oxidoreductase subunit | *nuoM* |
| WP_209102596.1 | NADH-quinone oxidoreductase subunit | *nuoL* |
| WP_209102597.1 | NADH-quinone oxidoreductase subunit | *nuoK* |
| WP_209102598.1 | NADH-quinone oxidoreductase subunit | *nuoJ* |
| WP_209102599.1 | NADH-quinone oxidoreductase subunit | *nuoI* |
| WP_209102600.1 | NADH-quinone oxidoreductase subunit | *nuoH* |
| WP_209102601.1 | NADH-quinone oxidoreductase subunit | *nuoG* |
| WP_209102604.1 | NADH-quinone oxidoreductase subunit | *nuoF* |
| WP_209102606.1 | NADH-quinone oxidoreductase subunit | *nuoE* |
| WP_209102609.1 | NADH-quinone oxidoreductase subunit | *nuoD* |
| WP_209102611.1 | NADH-quinone oxidoreductase subunit | *nuoC* |
| WP_209102613.1 | NADH-quinone oxidoreductase subunit | *nuoB* |
| WP_209102615.1 | NADH-quinone oxidoreductase subunit | *nuoA* |
| WP_209104532.1 | NADH:ubiquinone oxidoreductase subunit NDUFA12 |  |
| **Succinate dehydrogenase** (**respiratory complex II)** | | |
| WP_209105374.1 | ETC complex I subunit |  |
| WP_209101760.1 | succinate dehydrogenase iron-sulfur subunit | *sdhB* |
| WP_209101762.1 | succinate dehydrogenase flavoprotein subunit | *sdhA* |
| WP_247657747.1 | succinate dehydrogenase, hydrophobic membrane anchor protein | *sdhD* |
| WP_209101764.1 | succinate dehydrogenase, cytochrome b556 subunit | *sdhC* |
| **Cbb3 type cytochrome C oxidase** | |  |
| WP_209104398.1 | cytochrome-c oxidase, cbb3-type subunit I | *ccoN* |
| WP_209104399.1 | cytochrome-c oxidase, cbb3-type subunit II | *ccoO* |
| WP_209104400.1 | cbb3-type cytochrome c oxidase subunit 3 |  |
| WP_209104401.1 | cytochrome-c oxidase, cbb3-type subunit III | *ccoP* |
| WP_209104402.1 | cytochrome c oxidase accessory protein | *ccoG* |
| WP_209104403.1 | FixH family protein | *fixH* |
| WP_209104404.1 | cadmium-translocating P-type ATPase | *cadA* |
| WP_209104405.1 | cbb3-type cytochrome oxidase assembly protein | *ccoS* |
| **aa3 type cytochrome C oxidase** | |  |
| WP_209098639.1 | cytochrome c oxidase subunit II | *coxB* |
| WP_209098641.1 | cytochrome c oxidase subunit I | *ctaD* |
| WP_209098643.1 | heme o synthase | *cyoE* |
| WP_209098646.1 | cytochrome c oxidase assembly protein | *cox* |
| WP_209098648.1 | cytochrome c oxidase subunit 3 | *coxIII* |
| WP_209104008.1 | aa3-type cytochrome c oxidase subunit IV | *coxIV* |
| WP_247658189.1 | heme ABC exporter ATP-binding protein | *ccmA* |
| WP_247658191.1 | heme exporter protein | *ccmB* |
| WP_209104198.1 | DsbE family thiol:disulfide interchange protein | *dsbE* |
| WP_209104201.1 | heme ABC transporter permease |  |
| WP_209098666.1 | cytochrome c biogenesis protein | *ccdA* |
| **bd-type quinol oxidases** | | |
| WP_209105426.1 | ubiquinol oxidase subunit II | *cyoA* |
| WP_209100189.1 | cytochrome o ubiquinol oxidase subunit I | *cyoB* |
| WP_209100191.1 | cytochrome o ubiquinol oxidase subunit III | *cyoC* |
| WP_209100193.1 | cytochrome o ubiquinol oxidase subunit IV | *cyoD* |
| WP_247657669.1 | SURF1 family protein |  |
| WP_209103472.1 | cytochrome d ubiquinol oxidase subunit II | *cydB* |
| WP_209103473.1 | cytochrome ubiquinol oxidase subunit I | *cydA* |
| WP_209103474.1 | ubiquinone-dependent pyruvate dehydrogenase | *poxB* |

**Table S10.** Genes coding for nitrate reduction and utilization of fumarate, formate, and lactate in L1I39^T^ genome

| **Accession no.** | **Gene name** | **Gene** |
| --- | --- | --- |
| **Nitrate reduction** |  |  |
| WP_209103488.1 | MFS transporter | *narK* |
| WP_209103489.1 | nitrate reductase subunit alpha | *narG* |
| WP_209103490.1 | nitrate reductase subunit beta | *narH* |
| WP_209103491.1 | nitrate reductase molybdenum cofactor assembly chaperone | *narJ* |
| WP_209103492.1 | respiratory nitrate reductase subunit gamma | *narI* |
| WP_209103586.1 | ABC transporter substrate-binding protein | *ntrA* |
| WP_209103587.1 | nitrate ABC transporter permease | *ntrB* |
| WP_209103588.1 | ABC transporter ATP-binding protein | *tauB* |
| WP_209103589.1 | globin domain-containing protein | *nirB* |
| WP_209103590.1 | FAD-dependent oxidoreductase | *nirA* |
| WP_209103591.1 | NirA family protein | *nirA* |
| WP_209103592.1 | sulfite reductase subunit alpha | *cysJ* |
| WP_209103593.1 | nitrate reductase | *napA* |
| **Fumarate** |  |  |
| WP_209104827.1 | FumA C-terminus/TtdB family hydratase beta subunit | *fumA* |
| WP_209099727.1 | cation:dicarboxylase symporter family transporter | *dctA* |
| WP_209099728.1 | C4-dicarboxylate transporter | *dctA* |
| WP_209102170.1 | dicarboxylate/amino acid:cation symporter | *dctA* |
| WP_209102462.1 | class II fumarate hydratase | *fumC* |
| WP_209104667.1 | class II fumarate hydratase | *fumC* |
| **Formate utilization** | | |
| WP_209103581.1 | formate dehydrogenase subunit gamma | *fdsG* |
| WP_209103585.1 | formate dehydrogenase subunit delta | *fdsD* |
| WP_209104610.1 | formate dehydrogenase subunit gamma | *fdsG* |
| WP_209103583.1 | formate dehydrogenase subunit alpha | *fdhF* |
| WP_209104609.1 | formate dehydrogenase subunit beta | *fdxH* |
| WP_209102051.1 | oxalate/formate MFS antiporter | *oxlT* |
| WP_247658656.1 | oxalate/formate MFS antiporter | *oxlT* |
| WP_209103584.1 | formate dehydrogenase accessory sulfurtransferase | *fdhD* |
| WP_209104608.1 | formate dehydrogenase-N subunit alpha | *fdnG* |
| WP_209104611.1 | formate dehydrogenase accessory protein | *fdhE* |
| **Lactate utilization** | | |
| WP_209100620.1 | lactate utilization protein | *lutB* |
| WP_209100631.1 | LUD domain-containing protein |  |
| WP_209105089.1 | FAD-binding protein | *dldh* |
| WP_209099266.1 | FMN-dependent L-lactate dehydrogenase | *lldD* |
| WP_209099267.1 | FCD domain-containing protein | *lldR* |

**Table S11.** Genes coding for the central carbon metabolism pathway present in the L1I39^T^ genome

| **Accession no.** | **Gene name** | **Gene** |
| --- | --- | --- |
| **Entner–Doudoroff pathway** | | |
| [WP_209098452.1](https://www.ncbi.nlm.nih.gov/protein/WP_209098452.1?report=genbank&log$=protalign&blast_rank=1&RID=CD0M9T4F013) | Phosphopyruvate hydratase | *eno* |
| [WP_209099261.1](https://www.ncbi.nlm.nih.gov/protein/WP_209099261.1?report=genbank&log$=protalign&blast_rank=1&RID=CD4Z97T601R) | L-rhamnonate dehydratase | *gad* |
| [WP_209099515.1](https://www.ncbi.nlm.nih.gov/protein/WP_209099515.1?report=genbank&log$=protalign&blast_rank=1&RID=CD0MM05F013) | [ArsJ-associated glyceraldehyde-3-phosphate dehydrogenase](https://blast.ncbi.nlm.nih.gov/Blast.cgi#alnHdr_WP_209099515) | *gapdh* |
| [WP_209099584.1](https://www.ncbi.nlm.nih.gov/protein/WP_209099584.1?report=genbank&log$=protalign&blast_rank=1&RID=CD5RD1UM013) | Gluconolactonase/LRE family protein | *gnl* |
| [WP_209099814.1](https://www.ncbi.nlm.nih.gov/protein/WP_209099814.1?report=genbank&log$=protalign&blast_rank=1&RID=CD6B0YD5016) | Phosphogluconate dehydratase | *edd* |
| [WP_209100383.1](https://www.ncbi.nlm.nih.gov/protein/WP_209100383.1?report=genbank&log$=protalign&blast_rank=1&RID=CD5EYH69016) | UDP-glucose/GDP-mannose dehydrogenase family protein | *gdh* |
| [WP_209100672.1](https://www.ncbi.nlm.nih.gov/protein/WP_209100672.1?report=genbank&log$=protalign&blast_rank=1&RID=CD72D1K601R) | Bifunctional 4-hydroxy-2-oxoglutarate aldolase/2-dehydro-3-deoxy-phosphogluconate aldolase | *kdpg aldolase* |
| [WP_209100924.1](https://www.ncbi.nlm.nih.gov/protein/WP_209100924.1?report=genbank&log$=protalign&blast_rank=1&RID=CD0NA4EB016) | Pyruvate kinase | *pyk* |
| [WP_209101427.1](https://www.ncbi.nlm.nih.gov/protein/WP_209101427.1?report=genbank&log$=protalign&blast_rank=1&RID=CD0NP17N013) | Phosphoglycerate mutase family | *pgm* |
| [WP_209102043.1](https://www.ncbi.nlm.nih.gov/protein/WP_209102043.1?report=genbank&log$=protalign&blast_rank=1&RID=CD7TET6K01R) | Glucose-6-phosphate dehydrogenase | *g6pd* |
| [WP_209102045.1](https://www.ncbi.nlm.nih.gov/protein/WP_209102045.1?report=genbank&log$=protalign&blast_rank=1&RID=CD8C6EZ6013) | 6-phosphogluconolactonase | *6pgd* |
| [WP_209102118.1](https://www.ncbi.nlm.nih.gov/protein/WP_209102118.1?report=genbank&log$=protalign&blast_rank=1&RID=CD139YSA016) | Pyruvate kinase | *pyk* |
| [WP_209102914.1](https://www.ncbi.nlm.nih.gov/protein/WP_209102914.1?report=genbank&log$=protalign&blast_rank=1&RID=CD13UFPV013) | Phosphoglycerate kinase | *ppgk* |
| [WP_209102915.1](https://www.ncbi.nlm.nih.gov/protein/WP_209102915.1?report=genbank&log$=protalign&blast_rank=1&RID=CD142SXR013) | Type I glyceraldehyde-3-phosphate dehydrogenase | *gapdh* |
| [WP_209105145.1](https://www.ncbi.nlm.nih.gov/protein/WP_209105145.1?report=genbank&log$=protalign&blast_rank=1&RID=CD8SCVNW013) | Glycerate kinase | *glyK* |
| [WP_209104450.1](https://www.ncbi.nlm.nih.gov/protein/WP_209104450.1?report=genbank&log$=protalign&blast_rank=1&RID=CD14AV7X013) | Glucokinase | *gck* |
| [WP_209104912.1](https://www.ncbi.nlm.nih.gov/protein/WP_209104912.1?report=genbank&log$=protalign&blast_rank=1&RID=CD14JDM9016) | Phosphoglycerate mutase family protein | *pgm* |
| [WP_209105007.1](https://www.ncbi.nlm.nih.gov/protein/WP_209105007.1?report=genbank&log$=protalign&blast_rank=1&RID=CD14TJ1B016) | 2,3-bisphosphoglycerate-independent phosphoglycerate mutase | *pgmI* |
| **Glycolysis** |  |  |
| [WP_209098452.1](https://www.ncbi.nlm.nih.gov/protein/WP_209098452.1?report=genbank&log$=protalign&blast_rank=1&RID=CD0M9T4F013) | Phosphopyruvate hydratase/ enolase | *eno* |
| [WP_209099515.1](https://www.ncbi.nlm.nih.gov/protein/WP_209099515.1?report=genbank&log$=protalign&blast_rank=1&RID=CD0MM05F013) | [ArsJ-associated glyceraldehyde-3-phosphate dehydrogenase](https://blast.ncbi.nlm.nih.gov/Blast.cgi#alnHdr_WP_209099515) | *gapdh* |
| [WP_209105434.1](https://www.ncbi.nlm.nih.gov/protein/WP_209105434.1?report=genbank&log$=protalign&blast_rank=1&RID=CD0MXEKJ013) | Triosephosphate isomerase | *tim/tpiA* |
| [WP_209100924.1](https://www.ncbi.nlm.nih.gov/protein/WP_209100924.1?report=genbank&log$=protalign&blast_rank=1&RID=CD0NA4EB016) | Pyruvate kinase | *pyk* |
| [WP_209101427.1](https://www.ncbi.nlm.nih.gov/protein/WP_209101427.1?report=genbank&log$=protalign&blast_rank=1&RID=CD0NP17N013) | Phosphoglycerate mutase family | *pgm* |
| [WP_209102047.1](https://www.ncbi.nlm.nih.gov/protein/WP_209102047.1?report=genbank&log$=protalign&blast_rank=1&RID=CD16ASKT013) | Glucose-6-phosphate isomerase | *gpi* |
| [WP_209102118.1](https://www.ncbi.nlm.nih.gov/protein/WP_209102118.1?report=genbank&log$=protalign&blast_rank=1&RID=CD139YSA016) | Pyruvate kinase | *pyk* |
| [WP_209102913.1](https://www.ncbi.nlm.nih.gov/protein/WP_209102913.1?report=genbank&log$=protalign&blast_rank=1&RID=CD13K6J0013) | Fructose-bisphosphate aldolase class I | *fba* |
| [WP_209102914.1](https://www.ncbi.nlm.nih.gov/protein/WP_209102914.1?report=genbank&log$=protalign&blast_rank=1&RID=CD13UFPV013) | Phosphoglycerate kinase | *pgk* |
| [WP_209102915.1](https://www.ncbi.nlm.nih.gov/protein/WP_209102915.1?report=genbank&log$=protalign&blast_rank=1&RID=CD142SXR013) | Type I glyceraldehyde-3-phosphate dehydrogenase | *gapdh* |
| [WP_209104450.1](https://www.ncbi.nlm.nih.gov/protein/WP_209104450.1?report=genbank&log$=protalign&blast_rank=1&RID=CD14AV7X013) | Glucokinase | *gck* |
| [WP_209104912.1](https://www.ncbi.nlm.nih.gov/protein/WP_209104912.1?report=genbank&log$=protalign&blast_rank=1&RID=CD14JDM9016) | Phosphoglycerate mutase family protein | *pgm* |
| [WP_209105007.1](https://www.ncbi.nlm.nih.gov/protein/WP_209105007.1?report=genbank&log$=protalign&blast_rank=1&RID=CD14TJ1B016) | 2,3-bisphosphoglycerate-independent phosphoglycerate mutase | *pgmI* |
| **Gluconeogenesis** | | |
| [WP_209098452.1](https://www.ncbi.nlm.nih.gov/protein/WP_209098452.1?report=genbank&log$=protalign&blast_rank=1&RID=CD0M9T4F013) | Phosphopyruvate hydratase | *eno* |
| [WP_209099515.1](https://www.ncbi.nlm.nih.gov/protein/WP_209099515.1?report=genbank&log$=protalign&blast_rank=1&RID=CD0MM05F013) | [ArsJ-associated glyceraldehyde-3-phosphate dehydrogenase](https://blast.ncbi.nlm.nih.gov/Blast.cgi#alnHdr_WP_209099515) | *gapdh* |
| [WP_209100286.1](https://www.ncbi.nlm.nih.gov/protein/WP_209100286.1?report=genbank&log$=protalign&blast_rank=1&RID=CD4JXJHM01R) | Class II fructose-bisphosphatase | *glpX* |
| [WP_209105434.1](https://www.ncbi.nlm.nih.gov/protein/WP_209105434.1?report=genbank&log$=protalign&blast_rank=1&RID=CD0MXEKJ013) | Triosephosphate isomerase | *tim/tpiA* |
| [WP_209101427.1](https://www.ncbi.nlm.nih.gov/protein/WP_209101427.1?report=genbank&log$=protalign&blast_rank=1&RID=CD0NP17N013) | Phosphoglycerate mutase family | *pgm* |
| [WP_209102047.1](https://www.ncbi.nlm.nih.gov/protein/WP_209102047.1?report=genbank&log$=protalign&blast_rank=1&RID=CD16ASKT013) | Glucose-6-phosphate isomerase | *gpi* |
| [WP_209102709.1](https://www.ncbi.nlm.nih.gov/protein/WP_209102709.1?report=genbank&log$=protalign&blast_rank=1&RID=CD4KSF1201R) | Class 1 fructose-bisphosphatase | *glpX* |
| [WP_209102913.1](https://www.ncbi.nlm.nih.gov/protein/WP_209102913.1?report=genbank&log$=protalign&blast_rank=1&RID=CD13K6J0013) | Fructose-bisphosphate aldolase class I | *fba* |
| [WP_209102914.1](https://www.ncbi.nlm.nih.gov/protein/WP_209102914.1?report=genbank&log$=protalign&blast_rank=1&RID=CD13UFPV013) | Phosphoglycerate kinase | *pgk* |
| [WP_209102915.1](https://www.ncbi.nlm.nih.gov/protein/WP_209102915.1?report=genbank&log$=protalign&blast_rank=1&RID=CD142SXR013) | Type I glyceraldehyde-3-phosphate dehydrogenase | *gapdh* |
| [WP_209103447.1](https://www.ncbi.nlm.nih.gov/protein/WP_209103447.1?report=genbank&log$=protalign&blast_rank=1&RID=CD4KZE7H01R) | Phosphoenolpyruvate carboxykinase | *pck* |
| [WP_209104325.1](https://www.ncbi.nlm.nih.gov/protein/WP_209104325.1?report=genbank&log$=protalign&blast_rank=1&RID=CD4YD5V8013) | phosphoenolpyruvate carboxylase | *pepc* |
| [WP_209104912.1](https://www.ncbi.nlm.nih.gov/protein/WP_209104912.1?report=genbank&log$=protalign&blast_rank=1&RID=CD14JDM9016) | Phosphoglycerate mutase family protein | *pgm* |
| **Pentose phosphate pathway** | | |
| [WP_209098900.1](https://www.ncbi.nlm.nih.gov/protein/WP_209098900.1?report=genbank&log$=protalign&blast_rank=1&RID=CDEF80FV013) | Ribose-5-phosphate isomerase | *rpiA* |
| [WP_209099584.1](https://www.ncbi.nlm.nih.gov/protein/WP_209099584.1?report=genbank&log$=protalign&blast_rank=1&RID=CDEFFRMJ013) | SMP-30/gluconolactonase/LRE family protein | *gnl* |
| [WP_209102043.1](https://www.ncbi.nlm.nih.gov/protein/WP_209102043.1?report=genbank&log$=protalign&blast_rank=1&RID=CDEFMX9X016) | Glucose-6-phosphate dehydrogenase | *g6pd* |
| [WP_209102045.1](https://www.ncbi.nlm.nih.gov/protein/WP_209102045.1?report=genbank&log$=protalign&blast_rank=1&RID=CDEFUXXK016) | 6-phosphogluconolactonase | *6pgd* |
| [WP_209102100.1](https://www.ncbi.nlm.nih.gov/protein/WP_209102100.1?report=genbank&log$=protalign&blast_rank=1&RID=CDEFZPWX016) | Transaldolase | *tal* |
| [WP_209105519.1](https://www.ncbi.nlm.nih.gov/protein/WP_209105519.1?report=genbank&log$=protalign&blast_rank=1&RID=CDEG77MK016) | Ribulose-phosphate 3-epimerase | *rpe* |
| WP_247657819.1 | Transketolase | *tkt* |
| [WP_209103828.1](https://www.ncbi.nlm.nih.gov/protein/WP_209103828.1?report=genbank&log$=protalign&blast_rank=1&RID=CDES6WSR013) | Transketolase | *tkt* |
| [WP_209103829.1](https://www.ncbi.nlm.nih.gov/protein/WP_209103829.1?report=genbank&log$=protalign&blast_rank=1&RID=CDESBN22016) | Transketolase family protein | *tkt* |
| [WP_209105248.1](https://www.ncbi.nlm.nih.gov/protein/WP_209105248.1?report=genbank&log$=protalign&blast_rank=1&RID=CDFHXUZT01R) | Transketolase | *tkt* |
| [WP_209104122.1](https://www.ncbi.nlm.nih.gov/protein/WP_209104122.1?report=genbank&log$=protalign&blast_rank=1&RID=CDESRJS1016) | SMP-30/gluconolactonase/LRE family protein | *gnl* |
| [WP_209105042.1](https://www.ncbi.nlm.nih.gov/protein/WP_209105042.1?report=genbank&log$=protalign&blast_rank=1&RID=CDESXY71016) | Ribose-phosphate pyrophosphokinase | *prps* |
| **TCA cycle pathway** | | |
| [WP_209098274.1](https://www.ncbi.nlm.nih.gov/protein/WP_209098274.1?report=genbank&log$=protalign&blast_rank=1&RID=CDCTD15K016) | NADP-dependent isocitrate dehydrogenase | *icd* |
| [WP_209099677.1](https://www.ncbi.nlm.nih.gov/protein/WP_209099677.1?report=genbank&log$=protalign&blast_rank=1&RID=CDD43FJ401R) | dihydrolipoyl dehydrogenase | *pdh* |
| [WP_209102271.1](https://www.ncbi.nlm.nih.gov/protein/WP_209102271.1?report=genbank&log$=protalign&blast_rank=1&RID=CDD491GM01R) | Ldh family oxidoreductase | *mdh* |
| [WP_209102460.1](https://www.ncbi.nlm.nih.gov/protein/WP_209102460.1?report=genbank&log$=protalign&blast_rank=1&RID=CDD7GBKB013) | malate dehydrogenase | *mdh* |
| [WP_209102462.1](https://www.ncbi.nlm.nih.gov/protein/WP_209102462.1?report=genbank&log$=protalign&blast_rank=1&RID=CDD7TTVW013) | class II fumarate hydratase | *fumC* |
| [WP_209103984.1](https://www.ncbi.nlm.nih.gov/protein/WP_209103984.1?report=genbank&log$=protalign&blast_rank=1&RID=CDD7YFUB016) | malate dehydrogenase | *mdh* |
| [WP_209105050.1](https://www.ncbi.nlm.nih.gov/protein/WP_209105050.1?report=genbank&log$=protalign&blast_rank=1&RID=CDDKEG55013) | dihydrolipoyl dehydrogenase | *dld* |
| [WP_209105052.1](https://www.ncbi.nlm.nih.gov/protein/WP_209105052.1?report=genbank&log$=protalign&blast_rank=1&RID=CDDKP9VH013) | 2-oxoglutarate dehydrogenase complex dihydrolipoyllysine-residue succinyltransferase | *dlst* |
| [WP_209105054.1](https://www.ncbi.nlm.nih.gov/protein/WP_209105054.1?report=genbank&log$=protalign&blast_rank=1&RID=CDDKYFPR016) | 2-oxoglutarate dehydrogenase E1 component | *sucA* |
| [WP_209101760.1](https://www.ncbi.nlm.nih.gov/protein/WP_209101760.1?report=genbank&log$=protalign&blast_rank=1&RID=CDDSPSFK013) | succinate dehydrogenase iron-sulfur subunit | *sdhB/ frdB* |
| [WP_209101762.1](https://www.ncbi.nlm.nih.gov/protein/WP_209101762.1?report=genbank&log$=protalign&blast_rank=1&RID=CDDSZVWR013) | succinate dehydrogenase flavoprotein subunit | *sdhA* |
| [WP_247657747.1](https://www.ncbi.nlm.nih.gov/protein/WP_247657747.1?report=genbank&log$=protalign&blast_rank=1&RID=CDDT5F32013) | succinate dehydrogenase, hydrophobic membrane anchor protein | *sdh* |
| [WP_209101764.1](https://www.ncbi.nlm.nih.gov/protein/WP_209101764.1?report=genbank&log$=protalign&blast_rank=1&RID=CDDTFJWK016) | succinate dehydrogenase, cytochrome b556 subunit | *sdhC/frdC* |
| [WP_209104180.1](https://www.ncbi.nlm.nih.gov/protein/WP_209104180.1?report=genbank&log$=protalign&blast_rank=1&RID=CDDTRD2T013) | aconitate hydratase | *acnA* |
| [WP_209102337.1](https://www.ncbi.nlm.nih.gov/protein/WP_209102337.1?report=genbank&log$=protalign&blast_rank=1&RID=CDDU5BT6016) | citryl-CoA lyase | *gltA* |
| [WP_209102557.1](https://www.ncbi.nlm.nih.gov/protein/WP_209102557.1?report=genbank&log$=protalign&blast_rank=1&RID=CDDUGDVM013) | citrate synthase | *gltA* |
| [WP_209104827.1](https://www.ncbi.nlm.nih.gov/protein/WP_209104827.1?report=genbank&log$=protalign&blast_rank=1&RID=CDDUU7WX016) | FumA C-terminus/TtdB family hydratase beta subunit | *fumA* |
| [WP_209104667.1](https://www.ncbi.nlm.nih.gov/protein/WP_209104667.1?report=genbank&log$=protalign&blast_rank=1&RID=CDCUCHWN013) | class II fumarate hydratase | *fumC* |
| **Glyoxylate shunt pathway** | | |
| WP_209104093.1 | aldolase/citrate lyase family protein | *aclY* |
| WP_209104884.1 | aldolase/citrate lyase family protein | *aclY* |
| WP_209102030.1 | aldolase/citrate lyase family protein | *aclY* |
| WP_209099993.1 | malate synthase G | *glcB* |

**Table S12.** Assimilation of different carbon substrates by L1I39^T^ using BIOLOG Gen III microplate tests and API 20NE strip

| **Sugars** | **Growth** | **Relevant metabolic genes** | **Organic acids** | **Growth** | **Relevant metabolic genes** | **Aminoacids** | **Growth** | **Relevant metabolic genes** |
| --- | --- | --- | --- | --- | --- | --- | --- | --- |
| D-fucose | - | - | D-galacturonic acid | + | - | L-glutamic acid | + | NAD-specific glutamate dehydrogenase |
| L-fucose | - | - | methylpyruvate | + | - | L-pyroglutamic acid | + | - |
| D-raffinose | - | - | L-lactic acid | + | L-Lactate dehydrogenase | L-alanine | w | - |
| a-D-glucose | - | UDP-glucose dehydrogenase | citric acid | + | Citrate lyase | L-aspartic acid | w | Aspartate dehydrogenase |
| a-D-lactose | - | - | D-malic acid | + | Malate dehydrogenase | L-arginine | - | Arginine decarboxylase |
| D-mannose | - | GDP-mannose 4,6-dehydratase | propionic acid | + | 2-hydroxy-3-oxopropionate reductase | D-aspartic acid | - | Aspartate dehydrogenase homolog |
| D-maltose | - | maltose alpha-D-glucosyltransferase | L-malic acid | + | Malate dehydrogenase | L-histidine | - | - |
| D-melibiose | - | - | acetic acid | + | - | D-serine | - | - |
| D-fructose | - | Fructokinase | L-galactonic acid lactone | + | - | L-serine | - | L-serine dehydratase |
| D-trehalose | - | - | bromo-succinic acid | + | - |  |  |  |
| D-galactose | - | - | b-hydroxy-D,L-butyric acid | + | D-beta-hydroxybutyrate dehydrogenase |  |  |  |
| D-cellobiose | - | - | Formic acid | - | Formate dehydrogenase |  |  |  |
| gentiobiose | - | - | D-gluconic acid | - | Gluconate dehydratase |  |  |  |
| sucrose | - | - | D-glucuronic acid | - | - |  |  |  |
| D-turanose | - | - | acetoacetic acid | - | Acetoacetate decarboxylase |  |  |  |
| L-rhamnose | - | L-rhamnose-1-dehydrogenase | mucic acid | - | - |  |  |  |
| stachyose | - | - | quinic acid | - | - |  |  |  |
| arabinose | + | Arabinose 5-phosphate isomerase | N-acetyl neuraminic acid | - | - |  |  |  |
| **Sugar alcohols** | | | trisodium citrate | + | - |  |  |  |
| D-sorbitol | - | Sorbitol dehydrogenase | capric acid | + | - |  |  |  |
| D-mannitol | - | - | adipic acid | + | - |  |  |  |
| D-arabitol | - | - | phenylacetic acid | + | Phenylacetic acid degradation protein PaaD, thioesterase |  |  |  |

**Table S13.** The major genome features, including regulators, transporters, brackish adaptation, and other related functions, differentiate between 1, L1I39^T^ and its nearest phylogenetic neighbors: 2, *A. cavernae* Sn-9-2^T^; 3, *A. spiritensis* DSM 9035^T^.

| **Genome features** | **1** | **2** | **3** |
| --- | --- | --- | --- |
| Transposases | 3 | 3 | 29 |
| Regulatory genes | 309 | 254 | 290 |
| Sigma subunits | 20 | 13 | 17 |
| **Transport genes** | 759 | 581 | 692 |
| ABC | 365 | 260 | 294 |
| TRAP | 93 | 68 | 128 |
| MFS | 46 | 37 | 40 |
| Branched-chain aminoacid | 63 | 46 | 47 |
| **Brackish adaptation** |  |  |  |
| Sodium extrusion | 9 | 2 | 7 |
| Sodium uptake | 11 | 8 | 7 |
| **Plant interaction and related functions** | | | |
| ACC deaminase | 2 | 0 | 0 |
| Hydrogenase cluster | 19 | 0 | 0 |
| Type 6 secretion system | 2 clusters | 1 cluster | 1 cluster |

**Table S14.** List of aromatic degradation pathway-related genes encoded in the genome of L1I39^T^

| **Accession no.** | **Gene name** | **Gene** |
| --- | --- | --- |
| **Catechol (3-oxoadipate) pathway** | | |
| WP_209105256.1 | 3-oxoacid CoA-transferase subunit A | *catI* |
| WP_209103888.1 | 3-oxoacid CoA-transferase subunit B | *catJ* |
| WP_209105290.1 | 3-oxoacid CoA-transferase subunit A | *catI* |
| WP_209104218.1 | 3-oxoacid CoA-transferase subunit B | *catJ* |
| WP_209103902.1 | muconate/chloromuconate family cycloisomerase | *catB* |
| WP_209103901.1 | catechol 1,2-dioxygenase | *catA* |
| WP_209104216.1 | muconolactone Delta-isomerase | *catC* |
| WP_209104213.1 | 3-oxoadipyl-CoA thiolase | *catF* |
| WP_209103896.1 | aromatic acid/H+ symport family MFS transporter |  |
| **Phenylacetate (Paa) pathway** | | |
| WP_209102878.1 | phenylacetate--CoA ligase | *paaF* |
| WP_209104331.1 | 3-hydroxybutyryl-CoA dehydrogenase | *paaC* |
| WP_209102876.1 | 2-(1,2-epoxy-1,2-dihydrophenyl)acetyl-CoA isomerase | *paaG* |
| WP_209102874.1 | 3-oxoadipyl-CoA thiolase | *paaJ* |
| WP_209104662.1 | phenylacetate-CoA oxygenase/reductase subunit | *paaK* |
| WP_209105336.1 | phenylacetate-CoA oxygenase subunit | *paaJ* |
| WP_209104663.1 | phenylacetate-CoA oxygenase subunit | *paaC* |
| WP_209104664.1 | 1,2-phenylacetyl-CoA epoxidase subunit B | *paaB* |
| WP_209104665.1 | 1,2-phenylacetyl-CoA epoxidase subunit A | *paaA* |
| WP_209102877.1 | hydroxyphenylacetyl-CoA thioesterase | *paaI* |
| WP_209102875.1 | phenylacetic acid degradation protein | *paaN* |
| WP_209102873.1 | phenylacetic acid degradation bifunctional protein | *paaZ* |
| **4-hydroxyphenylacetate pathway** | | |
| WP_209099304.1 | 4-hydroxy-2-oxoheptanedioate aldolase | *hpcH* |
| WP_209099306.1 | 2-oxo-hepta-3-ene-1,7-dioic acid hydratase | *hpcG* |
| WP_209099307.1 | fumarylacetoacetate hydrolase family protein | *Fah* |
| WP_209099308.1 | 3,4-dihydroxyphenylacetate 2,3-dioxygenase | *hpaD* |
| WP_209099309.1 | 5-carboxymethyl-2-hydroxymuconate semialdehyde dehydrogenase | *hpaE* |
| WP_209099310.1 | 5-carboxymethyl-2-hydroxymuconate Delta-isomerase | *pcD* |
| WP_209099313.1 | Pyoverdin chromophore biosynthetic protein | *pvcC* |
| WP_209099311.1 | flavin reductase |  |

**Table S15**. List of genes coding for vitamin biosynthesis identified in the genome of L1I39^T^

| **Accession no.** | **Gene name** | **Gene** |
| --- | --- | --- |
| **Cobalamin (B12)** | | |
| WP_209105338.1 | Adenosylcobinamide-phosphate synthase | *cbiB* |
| WP_209105339.1 | Precorrin-6A synthase (deacetylating) | *cobF* |
| WP_209105340.1 | Cobyrinate a,c-diamide synthase | *cobB* |
| WP_209104742.1 | Precorrin-4 C(11)-methyltransferase | *cobM* |
| WP_209104744.1 | Precorrin-6y C5,15-methyltransferase (decarboxylating) subunit | *cbiE* |
| WP_209104746.1 | Cobalt-precorrin-6A reductase | *cbiJ* |
| WP_209104747.1 | Precorrin-3B C(17)-methyltransferase | *cobJ* |
| WP_209104749.1 | Precorrin-2 C(20)-methyltransferase | *cobI* |
| WP_209104751.1 | Precorrin-8X methylmutase | *cbiC* |
| WP_247658299.1 | Precorrin-3B synthase | *cobG* |
| WP_209104752.1 | Cobaltochelatase subunit | *cobN* |
| WP_209104754.1 | Cobalamin biosynthesis protein | *cobW* |
| WP_209104755.1 | DUF1636 family protein |  |
| WP_209104758.1 | Energy-coupling factor ABC transporter permease | *cbtC* |
| WP_209104759.1 | cob(I)yrinic acid a,c-diamide adenosyltransferase | *cobO* |
| WP_209104760.1 | Cobyric acid synthase | *cobQ* |
| WP_209104761.1 | EAL domain-containing protein |  |
| WP_209104762.1 | Threonine-phosphate decarboxylase | *cobD* |
| WP_209103279.1 | Cobaltochelatase subunit | *cobS* |
| WP_209103281.1 | Cobaltochelatase subunit | *cobT* |
| WP_209102984.1 | Cobalt ECF transporter T component | *cbiQ* |
| WP_247657830.1 | Cobalamin biosynthesis protein | *cbiM* |
| WP_209102985.1 | Cobalt transporter | *cbiM* |
| WP_209100005.1 | Adenosylcobinamide-GDP ribazoletransferase | *cobS* |
| **Biotin (H)** | | |
| WP_209099854.1 | DUF853 family protein (ATPase component BioM of energizing module of biotin ECF transporter) | *bioM* |
| WP_247657695.1 | GntR family transcriptional | *bioR* |
| WP_209099854.1 | SAM-dependent methyltransferase | *bioC* |
| WP_209103807.1 | GntR family transcriptional regulator | *bioR* |
| WP_209104023.1 | Biotin synthase | *bioB* |
| WP_209104024.1 | 8-amino-7-oxononanoate synthase | *bioF* |
| WP_209104025.1 | Dethiobiotin synthase | *bioD* |
| WP_209105271.1 | Adenosylmethionine--8-amino-7-oxononanoate transaminase | *bioA* |
| WP_209104026.1 | Beta-ketoacyl-ACP synthase III (Biotin synthesis protein) | *bioZ* |
| WP_209104904.1 | GntR family transcriptional regulator | *bioR* |
| WP_209104905.1 | Biotin transporter | *bioY* |
| WP_209103983.1 | Cell division protein | *zapE* |
| **Thiamine (B1)** | | |
| WP_209098819.1 | Glycine oxidase | *thiO* |
| WP_209098821.1 | Sulfur carrier protein | *thiS* |
| WP_209098823.1 | Thiazole synthase | *thiG* |
| WP_209098824.1 | Thiamine phosphate synthase | *thiE* |
| WP_209098826.1 | Thiamine pyrophosphate-binding protein | *thiB* |
| WP_209100588.1 | Thiamine phosphate synthase | *thiE* |
| WP_209102912.1 | Thiamine phosphate synthase | *thiE* |
| WP_209098596.1 | Thiamine-phosphate kinase | *thiL* |
| WP_209099983.1 | ABC transporter substrate-binding protein | ABC |
| WP_209099985.1 | ABC transporter ATP-binding protein | ABC |
| WP_209099987.1 | ABC transporter permease | ABC |
| WP_247657838.1 | ABC transporter substrate-binding protein | ABC |
| WP_209105157.1 | ABC transporter ATP-binding protein | ABC |
| WP_247657840.1 | ABC transporter permease | ABC |
| WP_209103636.1 | ABC transporter substrate-binding protein | ABC |
| WP_209103637.1 | ABC transporter permease | ABC |
| WP_209103638.1 | ABC transporter ATP-binding protein | ABC |
| WP_209103641.1 | ABC transporter substrate-binding protein | ABC |
| WP_209104677.1 | ABC transporter substrate-binding protein | ABC |
| WP_209104678.1 | ABC transporter ATP-binding protein | ABC |
| WP_209104679.1 | ABC transporter permease | ABC |
| WP_209101299.1 | Putative hydroxymethylpyrimidine transporter | *cytX* |
| WP_209102656.1 | ABC transporter substrate-binding protein | ABC |
| WP_209102658.1 | ABC transporter permease | ABC |
| WP_209098506.1 | Hydroxymethylpyrimidine phosphate synthase | *thiC* |
| WP_209105320.1 | Hydroxymethylpyrimidine phosphate kinase | *thiD* |
| **Riboflavin (B2)** | | |
| WP_209103301.1 | 3,4-dihydroxy-2-butanone-4-phosphate synthase | *ribA* |
| WP_247657602.1 | Diaminohydroxyphosphoribosylaminopyrimidine deaminase  / 5-amino-6-(5-phosphoribosylamino) uracil reductase | *ribB* |
| WP_209099064.1 | GTP cyclohydrolase II | *ribE* |
| WP_209098590.1 | RibD family protein | *ribD* |
| WP_209098592.1 | 6,7-dimethyl-8-ribityllumazine synthase | *ribH* |
| WP_209104251.1 | Riboflavin biosynthesis protein | *ribF* |
| WP_209098588.1 | Ribonucleotide reductase transcriptional regulator | *nrdR* |
| WP_209098594.1 | Transcription termination protein | *nusB* |
| **Folic acid (B9)** | | |
| WP_209099849.1 | 2-amino-4-hydroxy-6-hydroxymethyldihydropteridine diphosphokinase | *folK* |
| WP_247658566.1 | Dihydropteroate synthase | *folA* |
| WP_247658183.1 | Dihydrofolate reductase | *folC* |
| WP_209101235.1 | Bifunctional folylpolyglutamate synthase/dihydrofolate synthase | *folB* |
| WP_209102707.1 | Dihydroneopterin aldolase | *folP* |
| WP_209104699.1 | GTP cyclohydrolase I | *folE* |
| WP_209100159.1 | Bifunctional methylenetetrahydrofolate dehydrogenase/methenyltetrahydrofolate cyclohydrolase | *folD* |
| **Pyridoxine (B6)** | | |
| WP_209103294.1 | 1-deoxy-D-xylulose-5-phosphate synthase | *dxpS* |
| WP_209103678.1 | 4-hydroxythreonine-4-phosphate dehydrogenase | *pdxA* |
| WP_209104100.1 | 4-hydroxythreonine-4-phosphate dehydrogenase | *pdxA* |
| WP_209104127.1 | GntR family transcriptional regulator | *gntR* |
| WP_209104127.1 | Pyridoxal kinase | *pdxY* |
| WP_209102936.1 | Pyrimidine 5'-nucleotidase | pdxP |
| WP_209098500.1 | Pyridoxamine 5'-phosphate oxidase | *pdxK* |
| WP_209099873.1 | Pyridoxine 5'-phosphate synthase | *pdxJ* |
| **Pantothenate (B5)** | | |
| WP_209103993.1 | 2-dehydropantoate 2-reductase | *panE* |
| WP_209104355.1 | Dephospho-CoA kinase | *coaE* |
| WP_209104505.1 | Ketol-acid reductoisomerase | *ilvC* |
| WP_209104547.1 | Pantoate--beta-alanine ligase | *panC* |
| WP_209104788.1 | 2-dehydropantoate 2-reductase | *panE* |
| WP_209100114.1 | Phosphopantetheine adenylyltransferase | *coaD* |
| WP_209100847.1 | Pantothenate kinase | *panK* |
| WP_209101217.1 | Phosphopantothenoylcysteine decarboxylase / Phosphopantothenoylcysteine synthetase | *coaBC* |
| WP_209101466.1 | 3-methyl-2-oxobutanoate hydroxymethyltransferase | *panB* |
| WP_209099725.1 | Branched-chain amino acid aminotransferase | *ilvE* |
| **Lipoate** | | |
| WP_209099673.1 | Pyruvate dehydrogenase complex dihydrolipoamide acetyltransferase |  |
| WP_209099677.1 | Dihydrolipoyl dehydrogenase |  |
| WP_209099681.1 | Lipoyl synthase |  |
| WP_209100323.1 | Lipoyl(octanoyl) transferase | *lipB* |

**Table S16.** Sulfur assimilation genes encoded in the genome of L1I39^T^

| **Accession no.** | **Gene name** | **Gene** |
| --- | --- | --- |
| WP_209104478.1 | Sulfate adenylyltransferase subunit | *cysD* |
| WP_209104479.1 | Sulfate adenylyltransferase subunit | *cysN* |
| WP_209104480.1 | 3'(2'),5'-bisphosphate nucleotidase | *cysQ* |
| WP_209104481.1 | Sulfite exporter TauE/SafE family protein | *tauE* |
| WP_209099104.1 | ABC transporter substrate-binding protein | *tauA* |
| WP_247657610.1 | ABC transporter substrate-binding protein | *tauA* |
| WP_247657612.1 | ABC transporter permease | *tauC* |
| WP_209099206.1 | ABC transporter substrate-binding protein | *tauA* |
| WP_209099208.1 | ABC transporter permease | *tauC* |
| WP_209099210.1 | ATP-binding cassette domain-containing protein | *tauB* |
| WP_209099222.1 | ABC transporter ATP-binding protein | *tauB* |
| WP_209099224.1 | ABC transporter substrate-binding protein | *tauA* |
| WP_209099334.1 | MFS transporter | *tauC* |
| WP_209099336.1 | ABC transporter substrate-binding protein | *tauA* |
| WP_209099338.1 | ABC transporter ATP-binding protein | *tauB* |
| WP_209099340.1 | ABC transporter permease | *tauC* |
| WP_209099543.1 | ABC transporter permease subunit | *tauC* |
| WP_209099545.1 | ABC transporter ATP-binding protein | *tauB* |
| WP_209099547.1 | ABC transporter substrate-binding protein | *tauA* |
| WP_209100485.1 | ABC transporter substrate-binding protein | *tauA* |
| WP_209100486.1 | ABC transporter ATP-binding protein | *tauB* |
| WP_247658640.1 | Sulfate ABC transporter substrate-binding protein | *tauA* |
| WP_209102216.1 | ABC transporter substrate-binding protein | *tauA* |
| WP_209102218.1 | ABC transporter permease | *tauC* |
| WP_209102660.1 | ABC transporter ATP-binding protein | *tauB* |
| WP_209102833.1 | ABC transporter substrate-binding protein | *tauA* |
| WP_209102835.1 | ABC transporter ATP-binding protein | *tauB* |
| WP_209102948.1 | Alpha-ketoglutarate-dependent dioxygenase | *alkB* |
| WP_247657900.1 | ABC transporter substrate-binding protein | *tauA* |
| WP_209103178.1 | ABC transporter ATP-binding protein | *tauB* |
| WP_209103179.1 | ABC transporter permease | *tauC* |
| WP_209103180.1 | ABC transporter permease | *tauC* |
| WP_209103244.1 | Fumarate reductase/succinate dehydrogenase flavoprotein subunit | *frdA* |
| WP_209103346.1 | ABC transporter ATP-binding protein | *tauB* |
| WP_209103347.1 | ABC transporter permease | *tauC* |
| WP_209103707.1 | Sulfate ABC transporter substrate-binding protein | *cysP* |
| WP_209103708.1 | Sulfate ABC transporter permease subunit | *cysT* |
| WP_247658417.1 | Sulfate ABC transporter permease subunit | *cysW* |
| WP_209103709.1 | Sulfate ABC transporter ATP-binding protein | *cysA* |
| WP_209103801.1 | ABC transporter permease | *tauC* |
| WP_247658059.1 | Aliphatic sulfonate ABC transporter substrate-binding protein | *tauA* |
| WP_247658420.1 | ATP-binding cassette domain of the nitrate and sulfonate transporters | *tauB* |
| WP_247658061.1 | ABC transporter permease | *tauC* |
| WP_209103805.1 | ABC transporter permease | *tauC* |
| WP_209103806.1 | ABC transporter ATP-binding protein | *tauB* |
| WP_209103808.1 | ABC transporter substrate-binding protein | *tauA* |
| WP_209104386.1 | ATP-binding cassette domain of the nitrate and sulfonate transporters | *tauB* |
| WP_209104389.1 | ABC transporter substrate-binding protein | *tauA* |
| WP_209104844.1 | ABC transporter substrate-binding protein | *tauA* |
| WP_209104846.1 | ABC transporter permease | *tauC* |
| WP_209104478.1 | sulfate adenylyltransferase subunit | *cysD* |
| WP_209104479.1 | sulfate adenylyltransferase subunit | *cysN* |
| WP_209104480.1 | 3\\'(2\\'),5\\'-bisphosphate nucleotidase | *cysQ* |
| WP_209104481.1 | sulfite exporter TauE/SafE family protein | *tauE* |

**Table S17.** Phosphate assimilation genes present in the genome of L1I39^T^

| **Accession no.** | **Gene name** | **Gene** |
| --- | --- | --- |
| WP_209100383.1 | UDP-glucose/GDP-mannose dehydrogenase family protein | *gcd* |
| WP_209103715.1 | inorganic diphosphatase | *ppa* |
| WP_209102081.1 | exopolyphosphatase | *ppx* |
| WP_247657758.1 | Ppx/GppA family phosphatase | *ppx* |
| WP_209099772.1 | alpha-D-ribose 1-methylphosphonate 5-phosphate C-P-lyase | *phnJ* |
| WP_209099773.1 | carbon-phosphorus lyase complex subunit | *phnI* |
| WP_209099775.1 | phosphonate C-P lyase system protein | *phnG* |
| WP_209100385.1 | MBL fold metallo-hydrolase | *phnP* |
| WP_247658116.1 | ATP-binding protein/ Histidine kinase | *phoR* |
| WP_209103972.1 | phosphate regulon transcriptional regulator | *phoB* |
| WP_209101796.1 | hypothetical protein | *ugpQ* |
| WP_209103970.1 | phosphate ABC transporter ATP-binding protein | *pstB* |
| WP_209105275.1 | phosphate ABC transporter ATP-binding protein | *pstB* |
| WP_209103968.1 | phosphate ABC transporter permease subunit | *pstC* |
| WP_209103969.1 | phosphate ABC transporter permease | *pstA* |
| WP_209102523.1 | phosphonate ABC transporter ATP-binding protein | *phnC* |
| WP_209102518.1 | phosphonate ABC transporter, permease protein | *phnE* |
| WP_209102520.1 | phosphonate ABC transporter, permease protein | *phnE* |
| WP_209102974.1 | sn-glycerol-3-phosphate ABC transporter ATP-binding protein | *ugpC* |
| WP_209102975.1 | ABC transporter substrate-binding protein | *ugpB* |
| WP_247657828.1 | sugar ABC transporter permease | *ugpA* |
| WP_209102976.1 | carbohydrate ABC transporter permease | *ugpE* |
| WP_209104666.1 | alkylphosphonate utilization protein | *phnA* |
| WP_209099766.1 | phosphonate metabolism protein/1,5-bisphosphokinase | *phnN* |
| WP_209099768.1 | alpha-D-ribose 1-methylphosphonate 5-triphosphate diphosphatase | *phnM* |
| WP_247657641.1 | phosphonate C-P lyase system protein | *phnL* |
| WP_209099770.1 | phosphonate C-P lyase system protein | *phnK* |
| WP_209099772.1 | alpha-D-ribose 1-methylphosphonate 5-phosphate C-P-lyase | *phnJ* |
| WP_209099773.1 | carbon-phosphorus lyase complex subunit | *phnI* |
| WP_209099774.1 | phosphonate C-P lyase system protein | *phnH* |
| WP_209099775.1 | phosphonate C-P lyase system protein | *phnG* |
| WP_247657643.1 | phosphonate metabolism transcriptional regulator | *phnF* |
| WP_209103609.1 | Na/Pi symporter | *nptA* |
| WP_209103971.1 | phosphate signaling complex protein | *phoU* |
| WP_209103967.1 | phosphate ABC transporter substrate-binding protein | *pstS* |
| WP_209104338.1 | inorganic phosphate transporter family protein | *pitA* |
| WP_209100387.1 | Na/Pi cotransporter family protein | *nptA* |

**Table S18.** Genes coding for complete nitrogenase systems in the genome of L1I39^T^

| **Accession no.** | **Gene name** | **Gene** |
| --- | --- | --- |
| **Nitrogenase** | | |
| WP_209105224.1 | nitrogenase iron protein | *nifH* |
| WP_209103567.1 | nitrogenase molybdenum-iron protein alpha chain | *nifD* |
| WP_209103568.1 | nitrogenase molybdenum-iron protein subunit beta | *nifK* |
| WP_209103376.1 | putative nitrogen fixation protein | *nifT/fixU* |
| **FeMo-co biosynthesis** | | |
| WP_209103375.1 | nitrogen fixation protein | *nifZ* |
| WP_209103363.1 | nitrogenase stabilizing/protective protein | *nifW* |
| WP_209103366.1 | cysteine desulfurase | *nifS* |
| WP_209103367.1 | Fe-S cluster assembly protein | *nifU* |
| **Stabilization of nitrogenase** | | |
| WP_209103368.1 | iron-sulfur cluster assembly accessory protein | *HesB* |
| **Electron carriers** |  |  |
| WP_209098194.1 | 2Fe-2S iron-sulfur cluster-binding protein | *fdx* |
| WP_209098727.1 | (2Fe-2S) ferredoxin domain-containing protein | *fdx* |
| WP_209099325.1 | 2Fe-2S iron-sulfur cluster-binding protein | *fdx* |
| WP_209100507.1 | 2Fe-2S iron-sulfur cluster binding domain-containing protein | *fdx* |
| WP_209103373.1 | 4Fe-4S dicluster domain-containing protein | *fdx* |
| WP_209103573.1 | ferredoxin III, nif-specific | *fdxB* |
| WP_209103362.1 | electron transfer flavoprotein subunit beta/FixA family protein | *FixA* |
| WP_209104329.1 | electron transfer flavoprotein subunit beta/FixA family protein | *FixA* |
| WP_209103361.1 | electron transfer flavoprotein subunit beta/FixA family protein | *FixB* |
| WP_209101161.1 | TIGR03862 family flavoprotein | *FixC* |
| WP_209102120.1 | electron transfer flavoprotein-ubiquinone oxidoreductase | *FixC* |
| WP_209103359.1 | ferredoxin family protein | *FixX* |
| WP_209104491.1 | ferredoxin--NADP reductase | *fnr1* |
| WP_209100891.1 | ferredoxin--NADP reductase | *fnr1* |
| WP_209102224.1 | ferredoxin--NADP reductase | *fnr1* |
| WP_209102941.1 | indolepyruvate ferredoxin oxidoreductase family protein | *iorA* |
| WP_024277657.1 | MULTISPECIES: ferredoxin family protein | *PreA* |
| WP_209105063.1 | ferredoxin family protein | *PreA* |
| WP_209102222.1 | ferredoxin family protein | *PreA* |
| WP_209101762.1 | succinate dehydrogenase flavoprotein subunit | *sdhA* |
| WP_209103244.1 | fumarate reductase/succinate dehydrogenase flavoprotein subunit | *sdhA* |
| WP_209104492.1 | sulfite reductase flavoprotein subunit alpha | *CysJ* |
| WP_209103360.1 | FAD-dependent oxidoreductase |  |
| WP_209104330.1 | FAD-binding protein/ electron transfer flavoprotein subunit alpha |  |
| WP_209105138.1 | flavoprotein |  |
| **Sigma 54 RNA polymerase and its associated proteins** | | |
| WP_209099588.1 | sigma-54-dependent Fis family transcriptional regulator | *rpoF* |
| WP_209101225.1 | RNA polymerase factor sigma-54 | *rpoN* |
| WP_209103948.1 | PTS IIA-like nitrogen regulatory protein | *ptsN* |
| **Nif transcription regulators** | | |
| WP_209105202.1 | nif-specific transcriptional activator | *nifA* |
| **Two component regulators of N-fixation and assimilation** | | |
| WP_209105372.1 | nitrogen regulation protein NR(II) | *ntrB* |
| WP_209098308.1 | nitrogen regulation protein NR(I) | *ntrC* |
| WP_209098328.1 | sigma-54 dependent transcriptional regulator | *ntrY* |
| WP_209098330.1 | Trk system potassium transporter | *ntrX* |
| **Related to P_II_** |  |  |
| WP_209100254.1 | P-II family nitrogen regulator | *glnB* |
| WP_209101364.1 | P-II family nitrogen regulator |  |
| **Ammonium transporters** | | |
| WP_209103043.1 | ammonium transporter | *amtB* |
| WP_209099705.1 | ammonium transporter | *amtB* |
| WP_209101366.1 | ammonium transporter | *amtB* |
| **Nitrate assimilation and transport** | | |
| WP_209103593.1 | Nitrate reductase | *narX* |
| WP_209103489.1 | Nitrate reductase subunit alpha | *narG* |
| WP_209103490.1 | Nitrate reductase subunit beta | *narH* |
| WP_209103491.1 | Nitrate reductase molybdenum cofactor assembly chaperone | *narJ* |
| WP_209103492.1 | Respiratory nitrate reductase subunit gamma | *narI* |
| WP_209103590.1 | FAD-dependent oxidoreductase/Nitrite reductase |  |
| WP_209103591.1 | NirA family protein/ nitrite reductase | *nirA* |
| WP_209103587.1 | nitrate ABC transporter permease | *nrtB* |
| WP_209104032.1 | nitrate ABC transporter permease | *nrtB* |
| **Ammonium assimilation** | | |
| WP_209103995.1 | glutamate synthase subunit beta | *gltD* |
| WP_209103996.1 | glutamate synthase large subunit | *gltB* |
| WP_209104059.1 | sodium/glutamate symporter | *gltS* |
| WP_209103689.1 | glutamine synthetase family protein | *glnA* |
| WP_209100768.1 | glutamine synthetase family protein | *glnA* |
| WP_209103047.1 | FMN-binding glutamate synthase family protein | *glt* |
| WP_209102872.1 | FMN-binding glutamate synthase family protein | *glt* |
| WP_209099709.1 | FMN-binding glutamate synthase family protein | *glt* |
| WP_209101240.1 | glutamine synthetase beta-grasp domain-containing protein |  |

**Table S19**. Genes coding for urea hydrolysis in L1I39^T^ genome

| **Accession no.** | **Gene name** | **Gene** |
| --- | --- | --- |
| **Cluster 1** |  |  |
| WP_209103033.1 | LysR family transcriptional regulator | *lysR* |
| WP_209103034.1 | urease subunit gamma | *ureA* |
| WP_209103035.1 | urease subunit beta | *ureB* |
| WP_209103036.1 | urease subunit alpha | *ureC* |
| WP_209103039.1 | urease accessory protein | *ureE* |
| WP_247657852.1 | urease accessory protein | *ureF* |
| WP_209103040.1 | urease accessory protein | *ureG* |
| WP_209103041.1 | urease accessory protein | *ureD* |
| WP_209103042.1 | urea transporter | *yut* |
| WP_209103043.1 | ammonium transporter | *amtB* |
| **Cluster 2** |  |  |
| WP_209104413.1 | urea carboxylase | *uca* |
| WP_209104414.1 | urea carboxylase-associated family protein |  |
| WP_209104415.1 | urea carboxylase-associated family protein |  |
| WP_209104418.1 | putative urea ABC transporter substrate-binding protein | *urtA* |
| **Cluster 3** |  |  |
| WP_209104580.1 | urease accessory protein | *ureG* |
| WP_209105329.1 | urease accessory protein | *ureF* |
| WP_209104581.1 | urease accessory protein | *ureE* |
| WP_209104582.1 | urease subunit alpha | *ureC* |
| WP_209104585.1 | urease subunit gamma | *ureA* |
| WP_247658288.1 | urease accessory protein | *ureD* |
| WP_209104586.1 | urea ABC transporter ATP-binding subunit | *urtE* |
| WP_209104587.1 | urea ABC transporter ATP-binding protein | *urtD* |
| WP_209104588.1 | urea ABC transporter permease subunit | *urtC* |
| WP_209105331.1 | urea ABC transporter permease subunit | *urtB* |
| WP_247658457.1 | urea ABC transporter substrate-binding protein | *urtA* |

**Table S20.** List of iron acquisition-related gene clusters present in the genome of L1I39^T^

| **NCBI gene ID** | **Gene name** | **Genes** |
| --- | --- | --- |
| **Cluster-1** | |  |
| WP_209101957.1 | ATP-binding cassette domain-containing protein |  |
| WP_209101958.1 | Cyclic peptide export ABC transporter |  |
| WP_209101960.1 | Fe(3+)-hydroxamate ABC transporter permease | *fhuB* |
| WP_209101962.1 | Iron-siderophore ABC transporter substrate-binding protein |  |
| WP_209101963.1 | TonB-dependent siderophore receptor |  |
| WP_209101965.1 | FecR domain-containing protein |  |
| WP_209101966.1 | Sigma-70 family RNA polymerase sigma factor |  |
| **Cluster-2** | |  |
| WP_209103834.1 | Helix-turn-helix transcriptional regulator |  |
| WP_247658068.1 | RNA polymerase sigma factor |  |
| WP_247658422.1 | FecR family protein |  |
| WP_209103836.1 | TonB-dependent siderophore receptor |  |
| WP_209103837.1 | ABC transporter substrate-binding protein |  |
| WP_247658074.1 | Fe(3+)-hydroxamate ABC transporter permease | *fhuB* |
| WP_209103838.1 | Siderophore-iron reductase | *fhuF0* |
| WP_209103839.1 | Cyclic peptide export ABC transporter |  |
| WP_247658077.1 | ATP-binding cassette domain-containing protein |  |
| WP_209103840.1 | Haloacid dehalogenase type II |  |
| WP_209103841.1 | DUF692 family protein |  |
| WP_209103842.1 | AraC family transcriptional regulator |  |
| WP_247658080.1 | TonB-dependent siderophore receptor |  |
| WP_209103844.1 | ATP-binding cassette domain-containing protein |  |
| WP_209103845.1 | ABC transporter substrate-binding protein |  |
| WP_209105252.1 | Fe(3+)-hydroxamate ABC transporter permease | *fhuB* |
| WP_209103846.1 | Siderophore-interacting protein |  |
| **Cluster-3** | |  |
| WP_209103025.1 | TonB-dependent receptor |  |
| WP_209103026.1 | AraC family transcriptional regulator |  |
| WP_209103027.1 | ABC transporter ATP-binding protein |  |
| WP_209103028.1 | Iron ABC transporter permease |  |
| WP_209103029.1 | Siderophore-interacting protein |  |
| WP_209103030.1 | ABC transporter substrate-binding protein |  |
| WP_247657848.1 | TonB-dependent siderophore receptor |  |
| WP_247657850.1 | TetR/AcrR family transcriptional regulator |  |
| **Cluster-4** | |  |
| WP_209104850.1 | AraC family transcriptional regulator |  |
| WP_209104851.1 | TonB-dependent siderophore receptor |  |
| WP_209104852.1 | Alpha/beta hydrolase-fold protein |  |
| WP_209104853.1 | ABC transporter substrate-binding protein |  |
| WP_209104854.1 | Iron ABC transporter permease |  |
| WP_209104855.1 | ABC transporter ATP-binding protein |  |
| WP_209104856.1 | Siderophore-interacting protein |  |
| **Cluster-5** | |  |
| WP_209098752.1 | Ferrochelatase | *hemF* |
| WP_209098754.1 | Antibiotic biosynthesis monooxygenase |  |
| WP_209098756.1 | TonB-dependent hemoglobin/transferrin/lactoferrin family receptor |  |
| WP_209098758.1 | ABC transporter substrate-binding protein |  |
| WP_247657586.1 | Iron ABC transporter permease |  |
| WP_247657588.1 | Heme ABC transporter ATP-binding protein |  |
| **Cluster-6** | |  |
| WP_209100197.1 | HAMP domain-containing histidine kinase |  |
| WP_209100199.1 | Response regulator transcription factor |  |
| WP_209100201.1 | TonB-dependent receptor plug domain-containing protein |  |
| WP_209100203.1 | Hypothetical protein |  |
| WP_209100207.1 | Hemin uptake protein | *hemP* |
| **Cluster-7** | |  |
| WP_209102625.1 | TonB-dependent receptor |  |
| WP_209102627.1 | ABC transporter substrate-binding protein |  |
| WP_209102629.1 | Iron ABC transporter permease |  |
| WP_209102631.1 | ABC transporter ATP-binding protein |  |
| **Cluster-8** | |  |
| WP_209102256.1 | PepSY domain-containing protein |  |
| WP_209102258.1 | TonB-dependent siderophore receptor |  |
| WP_247657768.1 | FecR domain-containing protein |  |
| WP_247657770.1 | RNA polymerase sigma factor |  |
| **Cluster-9** | |  |
| WP_209100899.1 | TonB-dependent siderophore receptor |  |
| WP_209100900.1 | Fe^2+-^dependent dioxygenase |  |
| **Cluster-10** | |  |
| WP_209100358.1 | Iron ABC transporter permease |  |
| WP_209100360.1 | Fe(3+) ABC transporter substrate-binding protein |  |
| NZ_CP072392.1 | bacterioferritin | *bfr* |
| **Cluster-11** | |  |
| WP_247657713.1 | Siderophore-interacting protein |  |
| WP_247658596.1 | Transcriptional repressor/ Fur family ferric uptake regulator |  |
| **Other related genes** | |  |
| WP_209103208.1 | ferritin-like domain-containing protein |  |
| WP_209099386.1 | ferritin-like domain-containing protein |  |
| WP_209105070.1 | ferritin |  |

**Table S21.** List of genes related to hydrogenase cluster genes present in the L1I39^T^ genome.

| **Accession no.** | **Gene name** | **Gene** |
| --- | --- | --- |
| WP_209103735.1 | ATP-binding protein | *hupT* |
| WP_209103736.1 | HupU protein | *hupU* |
| WP_209103737.1 | nickel-dependent hydrogenase large subunit | *hupV* |
| WP_209103738.1 | carbamoyltransferase | *hypF* |
| WP_247658050.1 | hydrogenase small subunit | *hupS* |
| WP_209103739.1 | nickel-dependent hydrogenase large subunit | *hupL* |
| WP_209103740.1 | Ni/Fe-hydrogenase, b-type cytochrome subunit | *hupC* |
| WP_209103741.1 | HyaD/HybD family hydrogenase maturation endopeptidase | *hupD* |
| WP_209103742.1 | HypC/HybG/HupF family hydrogenase formation chaperone | *hupF* |
| WP_209103743.1 | hydrogenase | *hupG* |
| WP_209103744.1 | hydrogenase expression/formation protein | *hupH* |
| WP_209103745.1 | [NiFe]-hydrogenase assembly chaperone | *hupJ* |
| WP_209103746.1 | nickel-dependent hydrogenase large subunit | *hupK* |
| WP_209103747.1 | hydrogenase maturation nickel metallochaperone | *hypA* |
| WP_209103748.1 | hydrogenase nickel incorporation protein | *hypB* |
| WP_209103749.1 | sigma-54 dependent transcriptional regulator | *hupR* |
| WP_209103750.1 | HypC/HybG/HupF family hydrogenase formation chaperone | *hypC* |
| WP_209103751.1 | hydrogenase formation protein | *hypD* |
| WP_209103752.1 | hydrogenase expression/formation protein | *hypE* |

**Table S22.** Genes coding for acetoin and 2,3-butanediol production in L1I39^T^ genome

| **Gene ID** | **Gene product** |
| --- | --- |
| WP_209104506.1 | acetolactate synthase small subunit |
| WP_209104507.1 | acetolactate synthase 3 large subunit |
| WP_209104772.1 | 2,3-butanediol dehydrogenase |
| WP_209104773.1 | carboxymuconolactone decarboxylase family protein |
| WP_209104774.1 | glucose 1-dehydrogenase |
| WP_209104775.1 | acetoin dehydrogenase dihydrolipoyllysine-residue acetyltransferase subunit |
| WP_209104776.1 | alpha-ketoacid dehydrogenase subunit beta |
| WP_247658481.1 | thiamine pyrophosphate-dependent dehydrogenase E1 component subunit alpha |
| WP_209104778.1 | NAD(+)/NADH kinase |
| WP_209104779.1 | sigma-54-dependent Fis family transcriptional regulator |

**Table S23.** List of T1SS genes encoded in the genome of L1I39^T^

| **Gene ID** | **Gene product** |  |
| --- | --- | --- |
| WP_209102899.1 | GNAT family N-acetyltransferase/ Putative hemolysin | hlyA |
| WP_209105011.1 | ATP-binding cassette domain-containing protein/ | hlyB |
| WP_209098860.1 | NHLP family bacteriocin export ABC transporter peptidase/permease/ATPase subunit | hlyB |
| WP_209104326.1 | type I secretion system permease/ATPase | hlyB |
| WP_209103305.1 | hemolysin family protein | hlyC |
| WP_209104307.1 | hemolysin family protein | hlyC |
| WP_209103133.1 | HlyD family efflux transporter periplasmic adaptor subunit | hlyD |
| WP_209105009.1 | HlyD family type I secretion periplasmic adaptor subunit | hlyD |
| WP_209104327.1 | HlyD family type I secretion periplasmic adaptor subunit | hlyD |
| WP_209104396.1 | HlyD family efflux transporter periplasmic adaptor subunit | hlyD |
| WP_209103928.1 | efflux RND transporter periplasmic adaptor subunit | hlyD |
| WP_209099663.1 | TolC family protein | lapE |
| WP_209098248.1 | TolC family outer membrane protein | tolC |
| WP_209102899.1 | GNAT family N-acetyltransferase/ Putative hemolysin | hlyA |
| WP_209105011.1 | ATP-binding cassette domain-containing protein | hlyB |
| WP_209098860.1 | NHLP family bacteriocin export ABC transporter peptidase/permease/ATPase subunit | hlyB |

**Table S24.** List of T4SS genes encoded in the genome of L1I39^T^

| **Accession no.** | **Gene name** | **Gene** | **Vir homologues** |
| --- | --- | --- | --- |
| WP_209101863.1 | S26 family signal peptidase | *traF* |  |
| WP_209101865.1 | lytic transglycosylase domain-containing protein |  |  |
| WP_209101869.1 | relaxase/mobilization nuclease domain-containing protein | *traD* | *virD2* |
| WP_209101872.1 | conjugal transfer protein | *traG* | *virD4* |
| WP_209101874.1 | CopG family transcriptional regulator | *copG* |  |
| WP_209101876.1 | PIN domain-containing protein |  |  |
| WP_209101878.1 | SAM-dependent DNA methyltransferase |  |  |
| WP_209101880.1 | MerR family transcriptional regulator | *merR* |  |
| WP_209101882.1 | P-type conjugative transfer ATPase | *trbB* | *virB1* |
| WP_209101883.1 | TrbC/VirB2 family protein | *trbC* | *virB2* |
| WP_209101884.1 | VirB3 family type IV secretion system protein | *virB3* | *virB3* |
| WP_209101885.1 | conjugal transfer protein | *trbE* | *virB4* |
| WP_209101886.1 | P-type conjugative transfer protein | *trbJ* | *virB5* |
| WP_209101887.1 | putative entry exclusion protein | *trbK* |  |
| WP_209101888.1 | P-type conjugative transfer protein | *trbL* | *virB6* |
| WP_209101890.1 | conjugal transfer protein | *trbF* | *virB8* |
| WP_209101892.1 | P-type conjugative transfer protein | *trbG* | *virB9* |
| WP_209101895.1 | TrbI/VirB10 family protein | *trbI* | *virB10* |
| WP_209101897.1 | DUF2274 domain-containing protein |  |  |
| WP_247658293.1 | Ti-type conjugative transfer relaxase | *traA* |  |
| WP_209104724.1 | conjugal transfer protein | *traD* |  |
| WP_209104725.1 | conjugal transfer protein | *traD* |  |
| WP_209104726.1 | M48 family metallopeptidase |  |  |

**Table S25.** List of T6SS-related genes present in the genome of L1I39^T^

| **Accession no.** | **Gene name** | **Gene** |
| --- | --- | --- |
| **T6SS-1** | | |
| WP_209098510.1 | type VI secretion system baseplate subunit | *impH/ tssG* |
| WP_209098512.1 | type VI secretion system baseplate subunit | *impG/ tssF* |
| WP_209098514.1 | type VI secretion system baseplate subunit | *impF/ tssE* |
| WP_209098516.1 | hypothetical protein | *impE/ tagJ* |
| WP_209098518.1 | type VI secretion system contractile sheath large subunit | *impD/ tssC* |
| WP_209098520.1 | type VI secretion system contractile sheath large subunit | *impC/ tssC* |
| WP_209098522.1 | type VI secretion system contractile sheath small subunit | *impB/ tssB* |
| WP_209098524.1 | type VI secretion system protein | *impA/ tssA* |
| WP_209098526.1 | type VI secretion system membrane subunit | *icmF/ tssM* |
| WP_209098528.1 | type IVB secretion system protein | *impK/ tssL* |
| WP_209098530.1 | type VI secretion system baseplate subunit | *impJ/ tssK* |
| WP_209098531.1 | FHA domain-containing protein | *impI/ tagG* |
| WP_209098534.1 | hypothetical protein |  |
| WP_209098535.1 | type VI secretion system tip protein | *vgrG* |
| WP_209098537.1 | type VI secretion system tube protein | *hcp* |
| WP_209098540.1 | methyltransferase domain-containing protein |  |
| WP_209098541.1 | PAAR domain-containing protein |  |
| WP_209098542.1 | type VI secretion system ATPase | *clpB/ tssH* |
| WP_209098543.1 | type VI secretion system lipoprotein | *vasD/ tssJ* |
| WP_209098544.1 | MinD/ParA family protein |  |
| WP_209098545.1 | hypothetical protein |  |
| WP_209098558.1 | serine/threonine-protein phosphatase | *pppA* |
| WP_209098559.1 | thymidylate synthase |  |
| WP_209098560.1 | ferric reductase-like transmembrane domain-containing protein |  |
| WP_209098561.1 | serine/threonine protein kinase | *ppkA* |
| **T6SS-2** | | |
| WP_209103499.1 | type VI secretion system-associated protein | *impM/ tagF* |
| WP_209105218.1 | type VI secretion system membrane subunit | *icmF/ tssM* |
| WP_209103500.1 | type IVB secretion system protein |  |
| WP_209103501.1 | type VI secretion system baseplate subunit | *impJ/ tssK* |
| WP_209103502.1 | type VI secretion system-associated FHA domain protein | *impI/ tagG* |
| WP_209103503.1 | PAAR domain-containing protein |  |
| WP_209103504.1 | hypothetical protein |  |
| WP_209103505.1 | type VI secretion system tip protein | *vgrG* |
| WP_209103506.1 | type VI secretion system ImpA family N-terminal domain-containing protein | *impA/ tssA* |
| WP_209103507.1 | type VI secretion system contractile sheath small subunit | *impB/ tssB* |
| WP_209103508.1 | type VI secretion system contractile sheath large subunit | *impC/ tssC* |
| WP_209103509.1 | type VI secretion system tube protein Hcp | *impD/ tssC* |
| WP_209103510.1 | type VI secretion system baseplate subunit | *impF/ tssE* |
| WP_209103511.1 | type VI secretion system baseplate subunit | *impG/ tssF* |
| WP_209103512.1 | type VI secretion system baseplate subunit | *impH/ tssG* |
| WP_209103513.1 | type VI secretion system ATPase | *clpB/ tssH* |
| WP_209103516.1 | tetratricopeptide repeat protein |  |
| WP_209103517.1 | invasion associated locus B family protein |  |
| WP_209103518.1 | hypothetical protein |  |
| WP_209103519.1 | serine protease |  |
| WP_209103520.1 | hypothetical protein |  |
| WP_209103521.1 | hypothetical protein |  |
| WP_209103523.1 | serine/threonine protein kinase | *ppkA* |
| WP_209103524.1 | serine/threonine-protein phosphatase | *pppA* |

**Table S26.** List of effector proteins identified in L1I39^T^ genome

| **Accession no.** | **Gene name** | **Gene** |
| --- | --- | --- |
| WP_247657904.1 | tetratricopeptide repeat protein | *tpr* |
| WP_209103516.1 | tetratricopeptide repeat protein | *tpr* |
| WP_209103685.1 | tetratricopeptide repeat protein | *tpr* |
| WP_247658099.1 | tetratricopeptide repeat protein | *tpr* |
| WP_209104908.1 | tetratricopeptide repeat protein | *tpr* |
| WP_209098709.1 | tetratricopeptide repeat protein | *tpr* |
| WP_209099653.1 | tetratricopeptide repeat protein | *tpr* |
| WP_209100650.1 | tetratricopeptide repeat protein | *tpr* |
| WP_209100916.1 | tetratricopeptide repeat protein | *tpr* |
| WP_209102911.1 | sel1 repeat family protein | *Sel1* |
| WP_209102924.1 | sel1 repeat family protein | *Sel1* |
| WP_247658462.1 | sel1 repeat family protein | *Sel1* |
| WP_247657563.1 | sel1 repeat family protein | *Sel1* |
| WP_209101939.1 | SEL1-like repeat protein | *Sel1* |
| WP_209103127.1 | WD40 repeat domain-containing protein | *wd40* |
| WP_209103243.1 | HEAT repeat domain-containing protein | *heat* |
| WP_247658131.1 | Tol-Pal system beta propeller repeat protein | *tolB* |
| WP_247658305.1 | cysteine rich repeat-containing protein |  |
| WP_209099117.1 | FG-GAP-like repeat-containing protein | VCBS |

**Table S27.** List of brackish adaptation genes encoded in the genome of L1I39^T^

| **Accession no.** | **Gene name** | **Gene** |
| --- | --- | --- |
| **Sodium extrusion** | | |
| WP_209099991.1 | Na^+^/H^+^ antiporter |  |
| WP_209103466.1 | Na^+^/H^+^ antiporter |  |
| WP_209102781.1 | Na^+^/H^+^ antiporter subunit C |  |
| WP_209102783.1 | Na^+^/H^+^ antiporter subunit E |  |
| WP_209102787.1 | Na^+^/H^+^ antiporter subunit G |  |
| WP_209102779.1 | monovalent cation/H^+^ antiporter subunit A |  |
| WP_209105524.1 | monovalent cation/H^+^ antiporter subunit D |  |
| WP_209103823.1 | sodium:proton antiporter/ NhaP | *nhaP* |
| WP_209100682.1 | sodium:proton antiporter |  |
| **Sodium uptake** |  |  |
| WP_209098682.1 | bile acid:sodium symporter family protein |  |
| WP_209103385.1 | sodium:alanine symporter family protein |  |
| WP_209104059.1 | sodium/glutamate symporter |  |
| **Inorganic compatible solute uptake and extrusion** | |  |
| *Potassium* | | |
| WP_209102785.1 | K^+^/H^+^ antiporter subunit F |  |
| WP_209098330.1 | Trk system potassium transporter TrkA | *trkA* |
| WP_209098332.1 | TrkH family potassium uptake protein | *trkH* |
| WP_209103978.1 | TrkH family potassium uptake protein | *trkH* |
| WP_209104132.1 | potassium-transporting ATPase subunit | *kdpA* |
| WP_209104136.1 | potassium-transporting ATPase subunit | *kdpB* |
| WP_209104138.1 | K(+)-transporting ATPase subunit C | *kdpC* |
| WP_209103438.1 | Kef family K(+) transporter | *ybaL* |
| WP_209098636.1 | monovalent cation:proton antiporter-2 (CPA2) family protein | *kefB* |
| WP_209104763.1 | cation:proton antiporter | *kefC* |
| WP_209104184.1 | monovalent cation:proton antiporter-2 (CPA2) family protein | *kefC* |
| WP_209100721.1 | DUF3772 domain-containing protein Mechanosensitive channel | *mscK* |
| WP_209104140.1 | sensor histidine kinase | *kdpD* |
| WP_209103964.1 | KUP/HAK/KT family potassium transporter | *kup* |
| *Magnesium* | | |
| WP_209098497.1 | magnesium transporter CorA family protein | *corA* |
| WP_209100329.1 | magnesium transporter | *mgtE* |
| WP_209103119.1 | magnesium transporter | *mgtE* |
| *Others* |  |  |
| WP_209104534.1 | ionic transporter/ Calcium/proton antiporter | *chaA* |
| WP_209104283.1 | large conductance mechanosensitive channel protein | *mscL* |
| WP_247658317.1 | cyclic nucleotide-gated ion channel | *cng* |
| **Organic compatible solute biosynthesis and other transporters** | | |
| Glutamine, glutamate and proline | | |
| WP_209100768.1 | glutamine synthetase family protein | *glnA* |
| WP_209101240.1 | glutamine synthetase beta-grasp domain-containing protein | *glnS* |
| WP_209099709.1 | FMN-binding glutamate synthase family protein | *gltB* |
| WP_209104431.1 | NAD-glutamate dehydrogenase | *gdh* |
| WP_209099115.1 | glutamate 5-kinase | *proB* |
| WP_209099119.1 | glutamate-5-semialdehyde dehydrogenase | *proA* |
| WP_209105035.1 | pyrroline-5-carboxylate reductase | *proC* |
| WP_209103561.1 | trifunctional transcriptional regulator/proline dehydrogenase/L-glutamate gamma-semialdehyde dehydrogenase | *putA* |
| WP_209104738.1 | choline BCCT transporter | *betT* |
| *Trehalose* | | |
| WP_209102843.1 | alpha-trehalose-phosphate synthase (UDP-forming) | *otsA* |
| WP_209102850.1 | trehalose-phosphatase | *otsB* |
| *In complete N-acetylglutaminylglutamine amide (NAGGN) syhthesis* | | |
| WP_209102345.1 | N-acetylglutaminylglutamine amidotransferase/ Asparagine synthetase | *asnO* |
| WP_209102346.1 | class II glutamine amidotransferase |  |
|  |  |  |
